# Range and niche expansion through multiple interspecific hybridization: a genotyping by sequencing analysis of *Cherleria* (Caryophyllaceae)

**DOI:** 10.1186/s12862-020-01721-5

**Published:** 2021-03-10

**Authors:** Abigail J. Moore, Jennifer A. Messick, Joachim W. Kadereit

**Affiliations:** 1 Department of Microbiology and Plant Biology and Oklahoma Biological Survey, University of Oklahoma, 770 Van Vleet Oval, Norman, OK 73019 USA; 2 Department of Biology, University of Central Oklahoma, Howell Hall, Room 220, Edmond, OK 73034 USA; 3 Fachbereich Biologie, Institut Für Organismische Und Molekulare Evolutionsbiologie, Johannes Gutenberg-Universität Mainz, Anselm-Franz-von-Bentzel-Weg 9a, 55099 Mainz, Germany

**Keywords:** Phylogenomic, Radseq, *Minuartia*, Alps, European high mountains, Edaphic evolution, Calcicole, Calcifuge, Serpentine

## Abstract

**Background:**

*Cherleria* (Caryophyllaceae) is a circumboreal genus that also occurs in the high mountains of the northern hemisphere. In this study, we focus on a clade that diversified in the European High Mountains, which was identified using nuclear ribosomal (nrDNA) sequence data in a previous study. With the nrDNA data, all but one species was monophyletic, with little sequence variation within most species. Here, we use genotyping by sequencing (GBS) data to determine whether the nrDNA data showed the full picture of the evolution in the genomes of these species.

**Results:**

The overall relationships found with the GBS data were congruent with those from the nrDNA study. Most of the species were still monophyletic and many of the same subclades were recovered, including a clade of three narrow endemic species from Greece and a clade of largely calcifuge species. The GBS data provided additional resolution within the two species with the best sampling, *C. langii* and *C. laricifolia*, with structure that was congruent with geography. In addition, the GBS data showed significant hybridization between several species, including species whose ranges did not currently overlap.

**Conclusions:**

The hybridization led us to hypothesize that lineages came in contact on the Balkan Peninsula after they diverged, even when those lineages are no longer present on the Balkan Peninsula. Hybridization may also have helped lineages expand their niches to colonize new substrates and different areas. Not only do genome-wide data provide increased phylogenetic resolution of difficult nodes, they also give evidence for a more complex evolutionary history than what can be depicted by a simple, branching phylogeny.

**Supplementary information:**

**Supplementary information** accompanies this paper at 10.1186/s12862-020-01721-5.

## Background

The importance of hybridization in the generation of biological diversity is becoming increasingly clear [1, 10, 32]. Hybrid swarms or hybrid species, homoploid or polyploid, are the best-studied examples of hybridization [10, 33, 69]. However, introgression, the presence of a much smaller fraction of the genome of one species in another species, is also of great evolutionary significance [6, 11, 35, 68]. The generation of massive amounts of DNA sequence data through next generation sequencing has led to the recognition of introgression on a scale that was not previously suspected (e.g., [23, 31]).

While contemporary interspecific hybridization is relatively common in plants [49, 89], past hybridization sometimes took place when patterns of geographical distribution were very different from those of today. Species distribution ranges have been highly dynamic in response to past changes of climate. These patterns have been documented in detail, particularly for the most recent geological past, using various sources of evidence ([8, 20, 37, 73, 75, 76]. Climate-induced range shifts and climate-induced disturbance of the environment would have promoted the origin of hybrids between previously geographically or ecologically isolated taxa as well as their establishment in previously uninhabited areas [7, 11, 49, 79].

The European Alps are a model system for examining how a mountain biota has responded to climate change [20, 36, 72, 83]. The Alps were extensively glaciated during the Last Glacial Maximum (LGM) and previous glacial periods. The organisms living in the Alps survived these glaciations in refugia ([5, 53, 71, 72, 88], often implying substantial shifts of geographical range into lowland areas.

It has long been recognized that the flora of the Alps is intimately linked to that of neighboring mountain ranges, especially the Pyrenees, Apennines, Carpathians, Dinarids, and Balkans [2, 19, 25, 42, 70]. Due to their linkage, these mountain ranges together are considered the European Alpine System (EAS [59, 60]). Although glaciation was only local in many of the mountain ranges outside the Alps, substantial shifts in the elevation of the various habitat zones in response to Quaternary climatic oscillations occurred in all ranges. Thus, species exchange between mountain ranges would have been facilitated during glacial periods, but not during interglacial periods.

**Fig. 1:**
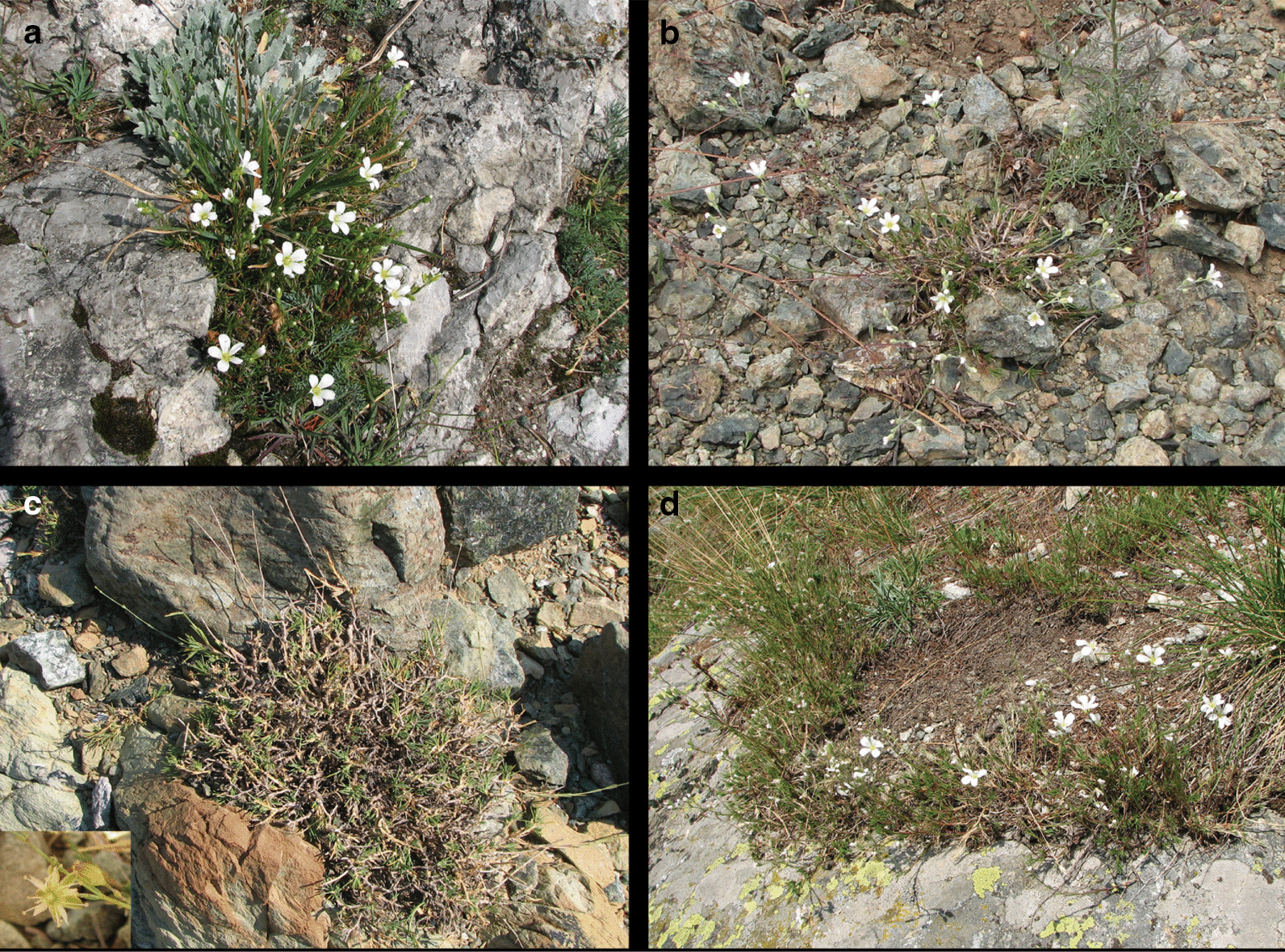
Representative photos of *Cherleria* species included in this study. *Cherleria langii* (Moore 1092, LN61), Dürre Wand, Niederösterreich, Austria (**a**); *Cherleria laricifolia* subsp. *ophiolitica* (Moore 1025, LO51), Miniera di Gambatesa, Liguria, Italy (**b**); *Cherleria dirphya* (Moore et al. 1513, DI230), Evvia, Greece (**c**); *Cherleria laricifolia* subsp. *laricifolia* (Moore et al. 1299, LL98), Vallone di Lourousa, Cuneo, Italy (**d**)

**Fig. 2:**
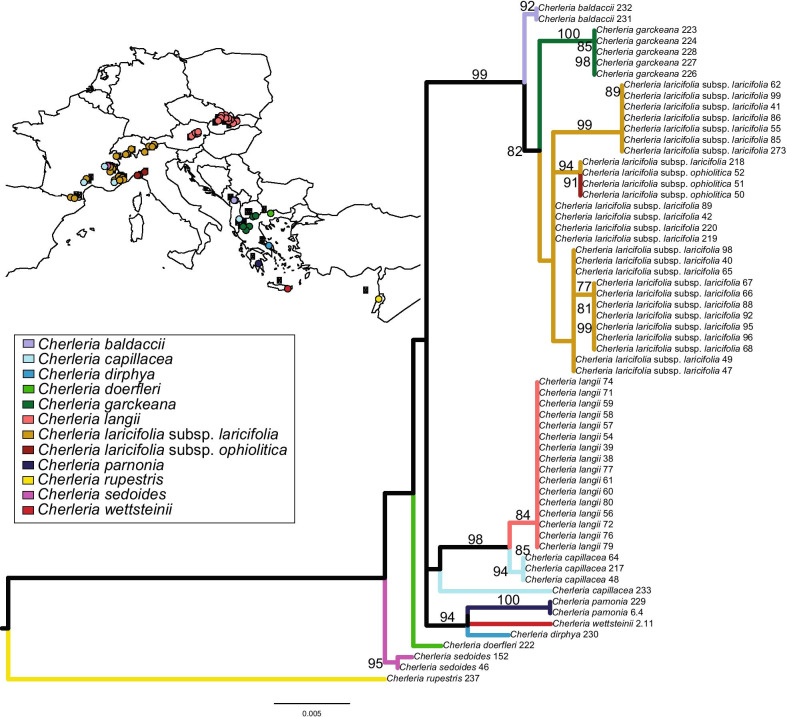
Phylogeny from the nuclear ribosomal DNA data set and map showing the sampling. Maximum likelihood phylogeny from RAxML, using nuclear ribosomal Internal Transcribed Spacer and External Transcribed Spacer data from the populations sampled in this study. Bootstrap values from 500 bootstrap replicates are above the branches; only values above 70% are shown. Inset shows the distribution of the sampled populations of each species. The colors of the points and the branches represent the different taxa

Given the dynamic range history of plant taxa in the EAS, we here examine the role of hybridization in the evolution of a subclade of *Cherleria* L. (formerly *Minuartia* L., Caryophyllaceae; [21, 54]) endemic to the EAS (Fig. 1). *Cherleria* as a whole contains 19 species and has a circumboreal distribution, with incursions into the high mountains of Eurasia and North America. It is most diverse in the EAS, with a clade of 11 species centered in the Alps and the Balkan Peninsula (Clade C [54]) based on nuclear ribosomal DNA, nrDNA, sequence data, Fig. 2). An analysis of biogeography and evolution of substrate preference showed that the group appeared to have initially diversified on the Balkan Peninsula, with multiple separate colonizations of the northern and western mountain ranges of the EAS [55]. More detailed work on two species of the group provided evidence for hybridization. A study of *C. sedoides* L. [57, 84] showed high chloroplast DNA (cpDNA) haplotype and nrDNA ribotype diversity throughout its wide range (including the Alps, the Balkan Peninsula, the Pyrenees, and Scotland). It also showed that the most common cpDNA haplotype clade most likely originated through hybridization of *C. sedoides* with an extinct species of the genus [57]. A study of *C. laricifolia* (L.) Iamonico using AFLPs and chloroplast haplotypes also showed high diversity throughout its range and evidence of gene flow between populations growing on serpentine and populations growing on other soils during the origin of the serpentine endemic ssp. *ophiolitica* (Pignatti) Iamonico [56].

Although the nrDNA phylogeny was relatively well-resolved and most species were recovered as monophyletic, we hypothesize that the actual evolutionary history of these species will be shown to be more complex and more instances of hybridization will be uncovered, when more of the genome is examined. In this study, we re-examine the EAS clade of *Cherleria* in both a phylogenetic and a phylogeographic context using genotyping by sequencing (GBS) data. In particular, we want to test the following hypotheses: (1) The nrDNA data, as used by Moore and Kadereit [55], are not entirely representative of the rest of the genome but represent an outlying clean signal. (2) Hybridization has been important in the evolution of the group. (3) Evidence of hybridization can provide information about the past distribution of these species, which could not have been inferred by other evidence.

## Results

### Sequencing

Of the plants we sequenced, 273 of the 290 were sequenced successfully. These plants had between 100,095 and 8,967,450 reads (mean ± standard deviation of 1,588,247 ± 1,281,318), of which 32,862 to 622,739 were unique (191,523 ± 125,227; Additional file 1). It is possible that some of these repeated reads were due to PCR duplicates, but as the sequenced fragments each start and end at a restriction site, instead of being randomly sheared, we did not have a way to distinguish PCR duplicates from independent sequences from the same locus. In the final analysis, 267 different loci were used in the Individual dataset (consisting of all sampled individuals), 352 were in the Population dataset (consisting of the single best individual from each population), and 380 were in the Taxon dataset (consisting of one composite individual from the single best population per taxon, Table 1 for composition of the Taxon dataset, Table 2 for characteristics of the individual datasets), when all species were analyzed together. When *C. laricifolia* was analyzed by itself (only at the Individual level), 465 loci were used, and when *C. langii* (G. Reuss) A.J. Moore & Dillenb. was analyzed by itself (also only at the Individual level), 404 loci were used (Table 2).

**Table 1:** Voucher and locality information

Species	Code	Locality	Collection	N	T
*baldaccii*	BA231	Albania, Tropojë	Moore et al. 1522	5	
*baldaccii*	BA232	Albania, Tropojë	Moore et al. 1523	5	*
*capillacea*	CA217	France, Languedoc-Roussillon, Hérault	Moore & Klein 1437	5	*
*capillacea*	CA233	Albania, Tropojë	Moore & Welch 1524	5	*
*capillacea*	CA48	France, Rhône-Alpes, Isère	Moore et al. 1193	5	
*capillacea*	CA53	Italy, Friuli-Venezia, Giulia, Pordenone	Moore 1044	0	
*capillacea*	CA64	France, Provence-Alpes-Côte d'Azur, Alpes de Haute Provence	Moore et al. 1241	5	
*dirphya*	DI230	Greece, Sterea Ellada, Evvia	Moore et al. 1513	5	*
*doerfleri*	DO222	Greece, Anatoliki Makedonia kai Thraki: Drama	Moore 1482	2	*
*garckeana*	GA223	Greece, Kentriki Makedonia, Kilkis	Moore 1493	5	
*garckeana*	GA224	Greece, Kentriki Makedonia, Pella	Moore 1500	4	
*garckeana*	GA226	Greece, Dytiki Makedonia, Grevena	Moore 1507	5	
*garckeana*	GA227	Greece, Epeiros, Ioannina	Moore 1508	5	
*garckeana*	GA228	Greece, Epeiros, Ioannina	Moore 1510	5	*
*langii*	LN38	Slovakia, Žilina, Krivánska Malá Fatra Mts	Moore 1084	5	
*langii*	LN39	Austria, Steiermark	Moore & Aoki 1117	5	
*langii*	LN54	Austria, Niederösterreich	Moore 1065	5	
*langii*	LN56	Slovakia, Košice, Slovenský raj Mts	Moore 1074	5	
*langii*	LN57	Slovakia, Banská Bystrica region, Muránska planina Mts	Moore & Blanár 1073	5	
*langii*	LN58	Slovakia, Žilina, near Liptovská Lúžna	Moore & Turis 1079	5	
*langii*	LN59	Slovakia, Žilina, Krivánska Malá Fatra Mts	Moore 1083	4	
*langii*	LN60	Slovakia, Trečin, Strážovské vrchy Mts	Moore et al. 1086	4	
*langii*	LN61	Austria, Niederösterreich	Moore 1092	5	
*langii*	LN71	Slovakia, Žilina, Nízke Tatry Mts	Moore & Turis 1080	5	
*langii*	LN72	Slovakia, Žilina, Chočské vrchy Mts	Moore & Turis 1081	3	
*langii*	LN74	Slovakia, Košice, Slovenský raj Mts	Moore & Dražil 1077	5	
*langii*	LN76	Slovakia, Trečin, Strážovské vrchy Mts	Moore & Smatanová 1088	4	
*langii*	LN77	Slovakia, Žilina, Strážovské vrchy Mts	Moore & Smatanová 1089	2	*
*langii*	LN79	Slovakia, Trečin, Strážovské vrchy Mts	Moore & Smatanová 1091	5	
*langii*	LN80	Austria, Niederösterreich	Moore 1112	5	
*laricifolia* subsp. *laricifolia*	LL40	Italy, Südtirol, Bozen	Moore & Aoki 1134	5	
*laricifolia* subsp. *laricifolia*	LL41	Italy, Südtirol, Bozen	Moore & Aoki 1155	5	
*laricifolia* subsp. *laricifolia*	LL42	Switzerland, Graubünden	Kadereit s.n	5	
*laricifolia* subsp. *laricifolia*	LL47	France, Rhône-Alpes, Isère	Moore et al. 1202	5	
*laricifolia* subsp. *laricifolia*	LL49	France, Rhône-Alpes, Isère	Moore et al. 1210	5	*
*laricifolia* subsp. *laricifolia*	LL55	Switzerland, Wallis	Moore et al. 1178	5	
*laricifolia* subsp. *laricifolia*	LL62	Austria, Tirol	Moore & Aoki 1119	4	
*laricifolia* subsp. *laricifolia*	LL65	France, Provence-Alpes-Côte d'Azur, Alpes de Haute Provence	Moore et al. 1245	5	
*laricifolia* subsp. *laricifolia*	LL66	France, Provence-Alpes-Côte d'Azur, Alpes Maritimes	Moore & Ichter 1252	4	
*laricifolia* subsp. *laricifolia*	LL67	France, Provence-Alpes-Côte d'Azur, Alpes Maritimes	Moore & Ichter 1268	5	
*laricifolia* subsp. *laricifolia*	LL68	Italy, Piemonte, Cuneo	Moore et al. 1291	5	
*laricifolia* subsp. *laricifolia*	LL85	Italy, Südtirol, Bozen	Moore & Aoki 1152	4	
*laricifolia* subsp. *laricifolia*	LL86	Switzerland, Wallis	Moore et al. 1187	4	
*laricifolia* subsp. *laricifolia*	LL88	France, Provence-Alpes-Côte d'Azur, Hautes Alpes	Moore et al. 1232	4	
*laricifolia* subsp. *laricifolia*	LL89	France, Provence-Alpes-Côte d'Azur, Hautes Alpes	Moore et al. 1234	5	
*laricifolia* subsp. *laricifolia*	LL92	France, Provence-Alpes-Côte d'Azur, Alpes Maritimes	Moore & Ichter 1260	4	
*laricifolia* subsp. *laricifolia*	LL95	France, Provence-Alpes-Côte d'Azur, Alpes Maritimes	Moore & Ichter 1278	2	
*laricifolia* subsp. *laricifolia*	LL96	France, Provence-Alpes-Côte d'Azur, Alpes Maritimes	Moore 1288	5	
*laricifolia* subsp. *laricifolia*	LL98	Italy, Piemonte, Cuneo	Moore et al. 1299	5	
*laricifolia* subsp. *laricifolia*	LL99	Switzerland, Tessin	Moore et al. 1300	5	
*laricifolia* subsp. *laricifolia*	LL218	France, Languedoc-Roussillon, Lozère	Moore & Klein 1449	5	
*laricifolia* subsp. *laricifolia*	LL219	France, Languedoc-Roussillon, Lozère	Moore & Klein 1450	3	
*laricifolia* subsp. *laricifolia*	LL220	France, Languedoc-Roussillon, Pyrénées-Orientales	Moore & Klein 1455	5	
*laricifolia* subsp. *laricifolia*	LL273	France, Rhône-Alpes, Haute Savoie	Moore & Dillenberger 1551	5	
*laricifolia* subsp. *ophiolitica*	LO50	Italy, Liguria, Genova	Moore 1031	5	*
*laricifolia* subsp. *ophiolitica*	LO51	Italy, Liguria, Genova	Moore 1025	5	
*laricifolia* subsp. *ophiolitica*	LO52	Italy, Emilia-Romagna, Parma	Moore 1038	5	
*parnonia*	PA	Greece, Peloponnisos, Arkadia	Kalpoutzakis 2022 (ACA)	4	
*parnonia*	PA229	Greece, Peloponnisos, Arkadia	Moore & Anastopoulos 1512	1	*
*rupestris*	LA237	Lebanon, Beqaa	Tohmé s.n	5	*
*sedoides*	SE152	France, Rhône-Alpes, Isère	Moore et al. 1203	1	*
*sedoides*	SE46	Austria, Tirol	Moore & Aoki 1133	1	
*wettsteinii*	WE	Greece, Crete: Lasithi	Papasotiropoulos & Trigas, DNA 2.11	5	x

Species, population code, sampling locality, voucher information, number of individuals included in the final dataset (N), and presence in the Taxon dataset (T). All vouchers are deposited at MJG, unless otherwise noted

**Table 2:** Characteristics of the datasets

Dataset	# Loci	# Individuals	Total length	Mean length	Prop. missing data	Mean # SNPs/locus
Individual	267	273	8099	30.33	19.6%	5.08
Population	352	65	10,450	29.69	16.9%	3.56
Taxon	380	13	10,109	26.60	24.1%	2.02
*C. laricifolia*	465	123	13,555	29.15	17.2%	1.89
*C. langii*	404	72	12,338	30.54	15.4%	1.46
nrDNA^a^	2	63	1117	558.50	15.9%	52.50

^a^nrDNA dataset has the same sampling as the population dataset

### Tree-based analyses

#### RAxML nrDNA tree

The nrDNA tree for the populations sampled in this study (Fig. 2; see Additional file 4: Fig. S1 for a comparison of the different tree topologies) was congruent with the wider sampling from Moore and Kadereit [55]. Plants were divided into a calcifuge clade (*C. baldaccii* (Halácsy) A.J. Moore & Dillenb., *C. garckeana* (Asch. & Sint. ex Boiss.) A.J. Moore & Dillenb., and *C. laricifolia*; 99% bootstrap support, BS), a calcicole clade (*C. langii* and the French populations of *C. capillacea* (All.) A.J. Moore & Dillenb.; 98% BS), and a clade containing the Greek endemics (*C. dirphya* (Trigas & Iatroú) A.J. Moore & Dillenb., *C. parnonia* (Kamari) A.J. Moore & Dillenb., and *C. wettsteinii* (Mattf.) A.J. Moore & Dillenb.; 94% BS). The relationships among these three groups and the samples of Albanian *C. capillacea*, *C. doerfleri* (Hayek) A.J. Moore & Dillenb., and *C. sedoides* were not resolved.

#### RAxML GBS tree

In the RAxML tree of the concatenated GBS data (Population dataset, the dataset composed of the best individual in each population; Additional file 4: Fig. S2), all species for which we had multiple samples were well supported as monophyletic with 100% bootstrap support, except for *C. capillacea*. In *C. capillacea*, the three French populations were monophyletic with 100% bootstrap support, but the Albanian population was separate. The Greek endemic species formed a clade (*C. dirphya*, *C. parnonia*, and *C. wettsteinii*; 98% BS). The three calcifuge species also formed a clade (*C. baldaccii*, *C. garckeana*, and *C. laricifolia*; 97% BS), with *C. garckeana* sister to *C. laricifolia* (97% BS). These calcifuge species formed a clade with the Albanian *C. capillacea* (pop. 223) and the Greek endemic clade (100% BS). Relationships within *C. laricifolia* and *C. langii* were generally not supported, with the exception of the three *C. laricifolia* samples from the Pyrenees/Massif Central (pops. 218, 219, and 220; 99% BS), and a few sister species pairs.

#### SVDQuartets

When SVDQuartets was used to make a species tree from the Population dataset (Additional file 4: Fig. S3), *C. laricifolia* and *C. garckeana* were sister (99.8% BS). The third calcifuge species, *C. baldaccii*, was sister to the Albanian population of *C. capillacea* (pop. 223) with no support. *Cherleria laricifolia* plus *C. garckeana* were sister to *C. baldaccii* plus Albanian *C. capillacea* (83.5% BS). The three Greek endemic species formed a clade (98.5% BS), which were in turn sister to the clade composed of the three calcifuge species plus Albanian *C. capillacea* (99.1% BS), but the relationships among the remaining species were not supported.

When a species-tree analysis was run on the Individual dataset (the dataset composed of all individuals; Additional file 4: Fig. S4), most of the same relationships were supported as in the species tree from the Population dataset, except that all three calcifuge species formed a clade (88.5% BS) and the Albanian population of *C. capillacea* (pop. 223) was sister to this calcifuge clade plus the Greek endemics (99.9% BS for the entire clade and 85.5% BS for the Greek endemics plus the calcifuge clade only).

To evaluate species monophyly, analyses were also run in which populations, instead of species, were the terminal taxa (called population trees). For the population tree from the Population dataset, most species for which multiple populations were sampled were recovered as monophyletic (Additional file 4: Fig. S5): *C. garckeana* (92.9% BS), *C. langii* (100.0% BS), *C. laricifolia* (71.1% BS), *C. parnonia* (95.9% BS), and *C. sedoides* (95.4% BS). There were two exceptions: In *C. capillacea*, the three French populations grouped together (99.6% BS), but were separated from the Albanian population (233). In *C. baldaccii*, population 231 formed a clade with *C. garckeana* and *C. laricifolia* (80.8% BS), while population 232 was the unsupported sister group of Albanian *C. capillacea*. These two clades were part of a larger clade that also included the three Greek endemic species (93.2% BS for the Greek endemics and 99.1% BS for the larger clade). There were two main clades within *C. laricifolia*: one composed of the southern Massif Central and Pyrenees samples (pops. 218, 219, and 220; 94.8% BS) and one of the remaining populations (73.7% BS). None of the other relationships within *C. laricifolia* was supported by bootstrap values greater than 70%.

**Fig. 3:**
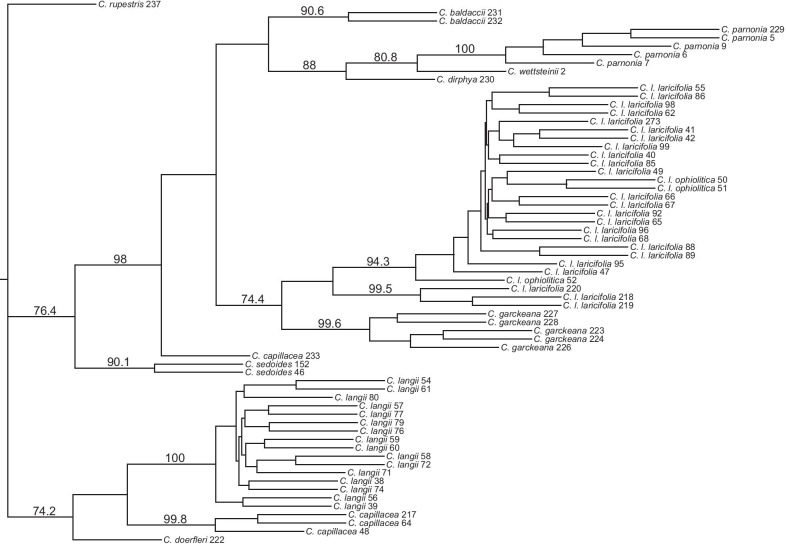
Population tree from SVDQuartets analysis of the Individual dataset. Bootstrap values from 1000 bootstrap replicates are above the branches; only values above 70% are shown

When the Individual dataset was used to construct a population-level tree, all but two species for which multiple individuals were sampled were well supported (Fig. 3). Once again, the Albanian population of *C. capillacea* (pop. 233) was clearly separate from the remaining three populations; instead, it was highly supported as the sister to the calcifuge and Greek endemic plants (98.0% BS). In addition, *C. laricifolia* was divided into two well-supported sister clades whose relationship was not supported. One of the clades was composed of the three populations from the Massif Central and Pyrenees (pops. 218, 219, and 220; 99.5% BS) and the other contained all remaining plants, including all three populations of subsp. *ophiolitica* (94.3% BS). *Cherleria garckeana* (99.6% BS) was again sister to *C. laricifolia*, but with lower support (74.4% BS). In contrast to the analysis of the Population dataset, *C. baldaccii* was monophyletic (90.6% BS), but was the unsupported sister to the Greek endemic clade, instead of to *C. garckeana* and *C. laricifolia*. The Greek endemic clade was monophyletic (88.0% BS), with *C. parnonia* (100.0% BS) sister to *C. wettsteinii*, with 80.8% BS. Both *C. langii* (100.0% BS) and the French populations of *C. capillacea* (99.8%) were well supported as monophyletic.

#### SplitsTree

In the SplitsTree analyses, relationships were generally congruent with those in the SVDQuartets analyses. In the tree from the Taxon dataset (Additional file 4: Fig. S6), *C. baldaccii*, *C. garckeana*, and *C. laricifolia* formed a moderately supported clade (86.4% BS). The two subspecies of *C. laricifolia* were also supported as a group (100% BS). The three Greek endemics (*C. dirphya*, *C. parnonia*, and *C. wettsteinii*) formed a strongly supported group (99.2% BS). These two clades and the Albanian population of *C. capillacea* (233) grouped together (94.5% BS), as did *C. langii* and the French *C. capillacea* (92.5%). There was also some conflict in the data. Within the calcifuge clade, *C. garckeana* grouped alternatively with *C. baldaccii* (79.4%) or with *C. laricifolia* (85.8%), and, within the Greek endemic clade, *C. parnonia* grouped alternatively with *C. wettsteinii* (70.8%) or with *C. dirphya* (96.9%).

In the tree from the Population dataset (Additional file 4: Fig. S7), *C. baldaccii*, *C. garckeana*, *C. langii*, *C. sedoides*, and the French populations of *C. capillacea* were all supported as monophyletic. Although *C. parnonia* was not supported as monophyletic by itself, the clade of all three Greek endemics was (92.3%). The calcifuge species and the Greek endemics together formed a clade with the Albanian *C. capillacea* population (92.9% BS). In addition to being unsupported as monophyletic, *C. laricifolia* was subtended by a very wide branch. Within *C. laricifolia*, the Pyrenean/Massif Central populations grouped together (97.9% BS), while the three populations of subsp. *ophiolitica* were mixed with the populations of Alpine subsp. *laricifolia*. *Cherleria baldaccii* was also on a very wide branch, although it was highly supported as monophyletic (100% BS). However, one of the populations of *C. baldaccii* also grouped with *C. garckeana*, *C. laricifolia*, and one population of *C. parnonia* with high support (92.8%, could not show split on figure).

### Single nucleotide polymorphism (SNP)-based analyses

#### *Adegenet* for all individuals

Only the Individual dataset was analyzed (Table 3). When the plants were divided into two groups (Additional file 4: Fig. S8a), the composition of the groups was the same in all ten runs: The first group contained all individuals of *C. doerfleri*, *C. langii*, and *C. sedoides*, as well as the three non-Albanian populations of *C. capillacea*, and the second contained the remaining plants. Although the composition of the two groups did not vary between runs, only the first group had good support (a-score, 0.863 ± 0.025), while the second group was much more poorly supported (a-score 0.036 ± 0.008).

**Table 3:** Groups recovered in the adegenet analyses of all individuals

	2 Groups	3 Groups	4 Groups	5 Groups	6 Groups	7 Groups
*laricifolia*		10, 0.174 ± 0.018	9, 0.117 ± 0.021	10, 0.109 ± 0.012	7, 0.105 ± 0.016	3, 0.137 ± 0.025
PMC^a^ *laricifolia*					2, 0.913 ± 0.021	7, 0.918 ± 0.012
Non-PMC *laricifolia*					2, 0.163 ± 0.017	6, 0.155 ± 0.014
*langii*		3, 0.859 ± 0.021	10, 0.836 ± 0.035	10, 0.820 ± 0.033	9, 0.800 ± 0.105	9, 0.765 ± 0.084
Non-Albanian *capillacea*			7, 0.932 ± 0.035	10, 0.927 ± 0.019	7, 0.926 ± 0.037	7, 0.927 ± 0.028
*langii*, non-Albanian *capillacea*		7, 0.728 ± 0.031				
*baldaccii*				1, 0.918	6, 0.897 ± 0.016	8, 0.918 ± 0.030
*garckeana*				3, 0.949 ± 0.017	9, 0.941 ± 0.024	9, 0.933 ± 0.028
*baldaccii*, *garckeana*			3, 0.934 ± 0.145	6, 0.951 ± 0.018	1, 0.921	
3 Greek endemics					1, 0.950	3, 0.926 ± 0.017
3 Greek endemics, Albanian *capillacea*					1, 0.979	3, 0.933 ± 0.005
*doerfleri*, *langii, sedoides*, non-Albanian *capillacea*	10, 0.863 ± 0.025					
remaining plants	10, 0.036 ± 0.008					
remaining plants (with non-Albanian *capillacea*)		3, 0.790 ± 0.019				
remaining plants (without non-Albanian *capillacea*)		7, 0.886 ± 0.022				

Only groups recovered from more than one total run are shown. Each cell contains the number of runs (from 1 to 10) in which that group was recovered, followed by the mean and standard deviation of the a-score for that group

^a^PMC is Pyrenees/Massif Central

When the plants were divided into three groups (Additional file 4: Fig. S8b), one group always consisted of *C. laricifolia*, but had low support (0.174 ± 0.018). The second group consisted of *C. langii* either by itself or with the French populations of *C. capillacea*. When the plants were divided into four groups (Additional file 4: Fig. S8c), the only group that was present in all runs was *C. langii*. The two other common groups were *C. laricifolia* and the French *C. capillacea*. When the plants were divided into five groups (Additional file 4: Fig. S8d), three groups were always present: *C. langii*, *C. laricifolia*, and the French *C. capillacea*. The remaining group (besides the group consisting of the leftover populations), was either *C. baldaccii*, *C. garckeana*, or the group formed of both *C. baldaccii* and *C. garckeana*. When the plants were divided into six groups (Additional file 4: Fig. S8e), no groups were present in all ten runs. The two most common groups were *C. langii* and *C. garckeana*. *Cherleria laricifolia* sometimes formed a single group and sometimes the three Pyrenees/Massif Central populations were separated from the remaining populations. The other two common groups were *C. baldaccii* and the French populations of *C. capillacea*. When the plants were divided into seven groups (Additional file 4: Fig. S8f), the two most common groups were once again *C. langii* and *C. garckeana*. *Cherleria laricifolia* was more commonly broken up into the Pyrenees/Massif Central populations and the remaining populations than it was recovered as a single group. The other common groups were *C. baldaccii*, the French populations of *C. capillacea*, and the three Greek endemic species (*C. dirphya*, *C. parnonia*, and *C. wettsteinii*), either with or without the Albanian population of *C. capillacea*.

Looking at admixture, there were no individuals that had a posterior probability more than 0.90 of being in multiple groups for the analyses in which the plants were divided into two, three, or four groups. For the remaining analyses, the only runs in which admixed individuals were found were those in which *C. langii* was divided into two groups (once each for the divisions into six and seven groups) or when the non-Pyrenees/Massif Central populations of *C. laricifolia* were divided into two groups (also once each for the divisions into six and seven groups). In these cases, some individuals showed admixture between these groups.

#### *Adegenet* for *Cherleria langii* alone

**Fig. 4:**
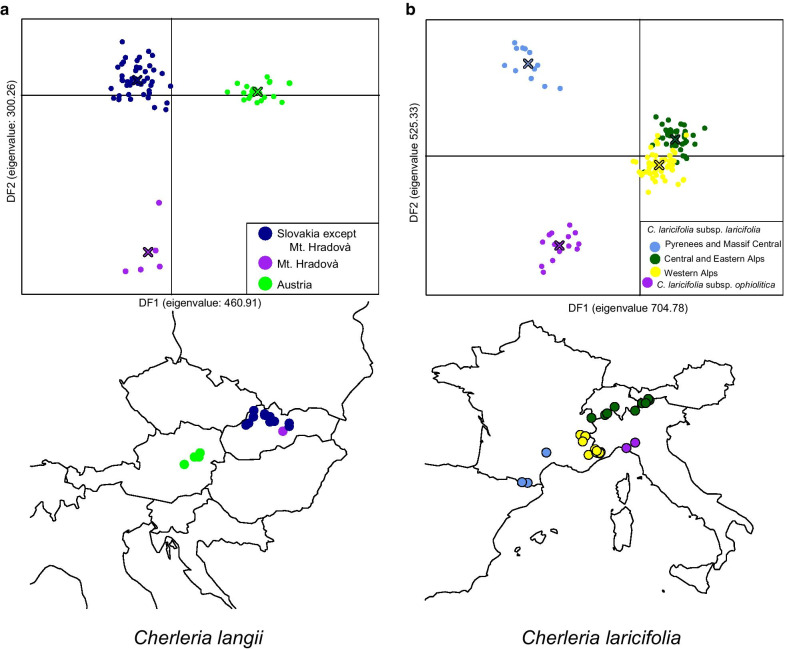
Representative plot from the adegenet analyses of *Cherleria langii* and *C. larcifiolia* alone. For *C. langii*, the plants were divided into three groups, with the distribution of the three groups shown below the plot (**a**). For *C. laricifolia*, the plants were divided into four groups, with the distribution of the four groups shown below the plot (**b**)

When *C. langii* was analyzed alone, the results were quite consistent from run to run (Table 4). When the plants were divided into two groups (Additional file 4: Fig. S9), one group consisted of the populations from Slovakia, while the other consisted of the populations from Austria. When the plants were divided into three groups (Fig. 4a), the Austrian group persisted, while the southernmost Slovakian population from Mt. Hradovà (pop. 57) was separated from the remaining Slovakian populations.

**Table 4:** Groups recovered in the adegenet analyses of *Cherleria langii* alone

	2 Groups	3 Groups
Austrian *langii*	10, 0.792 ± 0.031	10, 0.774 ± 0.038
Slovakian *langii*	10, 0.050 ± 0.012	
Slovakian *langii* without LN75		10, 0.068 ± 0.015
LN75		10, 0.836 ± 0.046

Each cell contains the number of runs (10 in all cases) in which that group was recovered, followed by the mean and standard deviation of the a-score for that group

#### *Adegenet* for *Cherleria laricifolia* alone

When *C. laricifolia* was analyzed alone, the results were also more consistent from run to run than when all taxa were analyzed together (Table 5). In all runs in which the individuals were divided into two groups (Additional file 4: Fig. S10a), the Pyrenees/Massif Central populations were separated from the remaining populations, which were much more poorly supported. When the individuals were divided into three clusters (Additional file 4: Fig. S10b), the Pyrenees/Massif Central populations were also present in every run. In seven cases, the remaining plants were divided into subsp. *ophiolitica* and the rest of subsp. *laricifolia*. In three cases, subsp. *ophiolitica* was combined with most of the French populations of subsp. *laricifolia* into one group, while the Italian, Swiss, and Austrian populations together with the north-eastern most French population (from Chamonix, pop. 273) of subsp. *laricifolia* formed the second group. When the individuals were divided into four clusters (Fig. 4b), the same four groups were always present: the Pyrenees/Massif Central populations of subsp. *laricifolia*, subsp. *ophiolitica*, the French populations of subsp. *laricifolia* except for the population from Chamonix (Western Alps populations), and the Italian, Swiss, and Austrian populations of subsp. *laricifolia* together with the French population from Chamonix (Central Alps populations).

**Table 5:** Groups recovered in the adegenet analyses of *Cherleria laricifolia* alone

	2 Groups	3 Groups	4 Groups
PMC^a^ *laricifolia*	10, 0.946 ± 0.027	10, 0.919 ± 0.034	10, 0.891 ± 0.042
Non-PMC *laricifolia*	10, 0.007 ± 0.003		
subsp. *ophiolitica*		7, 0.929 ± 0.032	10, 0.869 ± 0.035
Non-PMC subsp. *laricifolia*		7, 0.014 ± 0.005	
subsp. *ophiolitica*, French subsp. *laricifolia*		3, 0.590 ± 0.037	
French subsp. *laricifolia*			10, 0.539 ± 0.031
Italian, Swiss, Austrian subsp. *laricifolia* (and Chamonix)		3, 0.213 ± 0.016	10, 0.283 ± 0.023

Each cell contains the number of runs (from 1 to 10) in which that group was recovered, followed by the mean and standard deviation of the a-score for that group

^a^PMC is Pyrenees/Massif Central

#### fineRADstructure

**Fig. 5:**
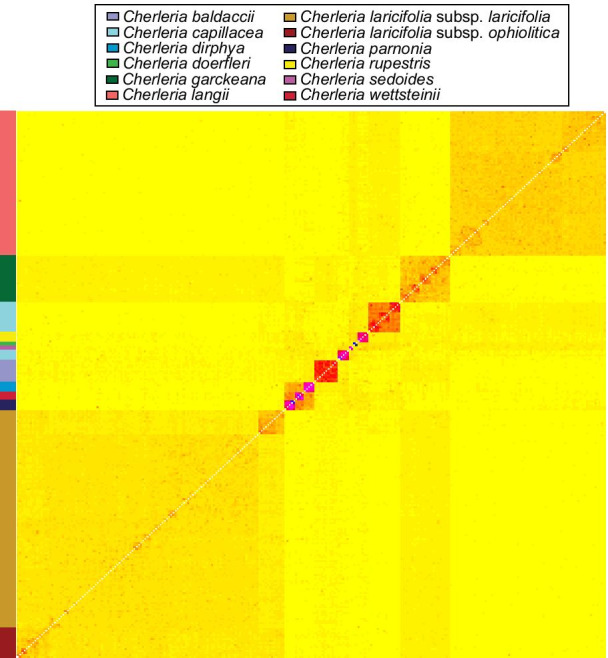
Plot from the fineRADstructure analysis of all individuals. The color bar on the left side of the figure represents the different species. The colors correspond to those in Fig. 1

When all individuals were analyzed together, *fineRADstructure* recovered all species as their own group, with *C. capillacea* being divided into two groups, one of the French populations and one of the Albanian population (Fig. 5). The groups varied in the strength of similarity among their members. The Albanian populations of *C. capillacea*, *C. doerfleri*, *C. rupestris* (Labill.) A.J. Moore & Dillenb., and *C. sedoides*, along with each of the three Greek endemic species each formed a strong group. *Cherleria baldaccii*, the French populations of *C. capillacea*, and the group formed by the three Greek endemic species formed moderately strong groups. *Cherleria garckeana*, *C. langii*, and *C. laricifolia* each formed week groups, with the Pyrenees/Massif Central populations forming a weak group within *C. laricifolia* as a whole.

When *C. laricifolia* was analyzed alone, the Pyrenees/Massif Central populations formed their own group, as they did when all individuals were analyzed together (Additional file 4: Fig. S11). In contrast to the analysis of all species, the individuals of subsp. *ophiolitica* also grouped together. There were several other very weak groups, which corresponded to individual populations.

When *C. langii* was analyzed alone, there were no strong patterns (Additional file 4: Fig. S12). The patterns that existed were largely groupings of the plants from an individual population. The samples were also weekly grouped by geography, with the Austrian samples forming a weak group, which was separated first.

#### D-statistics

The two sets of analyses, those with the groups formed based on the SVDQuartets tree and those with the groups formed based on the RAxML tree, gave congruent results (see Additional file 2). The two species with the most introgression were *C. garckeana* and *C. laricifolia*. They both showed introgression > 5% with each other, French *C. capillacea*, *C. parnonia*, and the common ancestor of *C. parnonia* and *C. wettsteinii*. In addition, *C. laricifolia* had introgression with *C. baldaccii*, and French *C. capillacea* showed introgression with *C. langii* (RAxML only). Although there were 149 total significant tests involving *C. langii* and *C. laricifolia* over both trees, this appeared to be because these were the two species with the highest sampling, as no individual had more than 0.3% of tests significant, and most had < 0.1% of tests significant.

### Species distribution modeling

#### *Cherleria capillacea*

For *Cherleria capillacea*, the following variables were used in the final analysis: Bio12 (mean annual precipitation), the variable with the highest gain in isolation; pH, the variable with the greatest unique contribution, when it was included; Bio15 (precipitation seasonality), the variable with the greatest unique contribution, when pH was not included; Bio5 (maximum temperature of the warmest month), Bio8 (mean temperature of the wettest quarter), and Bio11 (mean temperature of the coldest quarter), all of which had significant contributions or importances; and Bio4 (temperature seasonality), which did not have a significant contribution but was not correlated with other variables.

The values for the area under the receiver operator characteristic (ROC) curve (AUC values) ranged from 0.921 to 0.945. Congruent with the results of the preliminary analyses, Bio12 (mean annual precipitation) had the greatest individual contribution to the model (percent contribution 42.9–49.1). In contrast to the results of the preliminary analyses, Bio11 (mean temperature of the coldest quarter) had the greatest loss when it was left out, in all but one case (percent importance 26.5–45.2). Bio8 (mean temperature of the wettest quarter) had both the next highest gain in isolation (percent contribution 22.6–25.1). When included, pH had a relatively small contribution (percent contribution 6.9–9.1).

**Fig. 6:**
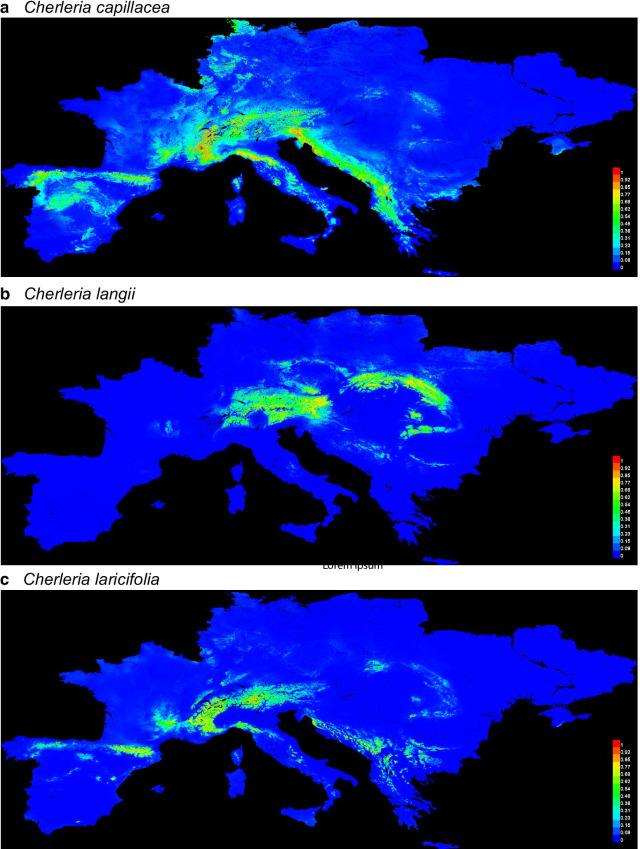
Modeled species distributions for *Cherleria capillacea*, *C. langii*, and *C. laricifolia*. Models were from MaxEnt based on BioClim variables and pH. Maps show the model of the current distribution with the occurrence records used to create the model for *C. capillacea* (**a**), *C. langii* (**b**), and *C. laricifolia* (**c**)

For *C. capillacea*, both of the current climate models (with and without pH included) had a high probability of occurrence in the central and western Alps, northern Apennines, Massif Central, Pyrenees, and all along the western side of the Balkan Peninsula from Croatia to Greece (Figs. 6a, S13d). The model without pH also included a potential area of occurrence in south-western Germany. The models with pH supported an LGM distribution that was more or less equivalent to its current range, as well as areas in north-western France, Poland, and the Ukraine (Figs. S13a–c). However, the occurrence of *C. capillacea* in these areas all had a probability < 0.70. The models without pH included not having any areas of occurrence with a probability > 0.25 (Additional file 5: Fig. S13e–g).

#### *Cherleria langii*

For *Cherleria langii*, the following variables were used in the final analysis: Bio18 (precipitation of the warmest quarter), the variable with the highest gain in isolation; Bio8 (mean temperature of the wettest quarter), the variable with the greatest unique contribution, whether or not pH was included; Bio9 (mean temperature of the driest quarter), Bio12 (mean annual precipitation), Bio15 (precipitation seasonality), and pH, all of which had significant contributions or importances; and Bio2 (mean diurnal temperature range) and Bio4 (temperature seasonality), which did not have significant contributions but were not correlated with other variables.

The AUC values ranged from 0.962 to 0.969. Congruent with the results of the preliminary analyses, Bio18 (precipitation of the warmest quarter) had the greatest individual contribution to the model (percent contribution 65–81.9). In contrast to the results of the preliminary analyses, Bio9 (mean temperature of the wettest quarter) had the greatest unique contribution (percent importance of 63.8–77.1). In these analyses, it also had the greatest loss when it was left out, likely due to the omission of the variables with which it was correlated. When pH was included, it had the next highest gain in isolation (percent contribution 14.7–18.8), while Bio15 (precipitation seasonality) had the second or third highest gain in isolation, depending on whether pH was present or not (percent contribution 8–9.6). In addition to having the highest gain in isolation, Bio18 had the second highest unique contribution in all but one case (percent importance 9.4–16.5, with an outlying value of 3.5), with Bio12 (mean annual precipitation) having the highest unique contribution in that case (percent importance 11.5 for that model and 5.8–12.8 otherwise).

For *C. langii*, the current distribution was approximately the same with or without pH included, and corresponded closely to the current distribution of the eastern Alps and Carpathians (Figs. 6b, S14d). The predicted distribution extended farther west than the current distribution, however, as well as into southern Germany. None of the LGM models showed any areas with a probability of occurrence > 0.10 (Figs. S14a–c, e–g).

#### *Cherleria laricifolia*

For *Cherleria laricifolia*, the following variables were used in the final analysis: Bio10 (mean temperature of the warmest quarter), the variable with the highest gain in isolation; Bio9 (mean temperature of the driest quarter), the variable with the greatest unique contribution, when pH was included; Bio19 (precipitation of the coldest quarter), the variable with the greatest unique contribution, when pH was not included; Bio8 (mean temperature of the wettest quarter), which also had a significant contribution; and Bio2 (mean diurnal temperature range), Bio4 (temperature seasonality), Bio17 (precipitation of the driest quarter), and pH, which did not have significant contributions but were not correlated with other variables.

The AUC values ranged from 0.964 to 0.973. Congruent with the results of the preliminary analyses, Bio10 (mean temperature of the warmest quarter) had the greatest individual contribution to the model (percent contribution 68–69). In these analyses, it also had the greatest loss when it was left out, likely due to the omission of the variables with which it was correlated (percent importance of 45.8–55.8). Bio8 (mean temperature of the wettest quarter) had the next highest gain in isolation (percent contribution 11.6–14.1), while Bio17 (precipitation of the driest quarter) and Bio4 (temperature seasonality) had the second highest unique contribution (Bio17, 4 models, 10.4–16.8; Bio4, 2 models, 6.3–15.8). When included, pH had a relatively small contribution (percent contribution 5.8–6.1).

For *C. laricifolia*, both of the current climate models (with and without pH included) had a range that included its current range in the western and central Alps, the Massif Central, the Pyrenees, and the northern Apennines, with additional occurrence probabilities in the northern part of the Balkan Peninsula, the central Alps, and the Carpathians (Figs. 6c, S15d). Standard deviations between model runs were very low across the entire range. None of the LGM models showed probabilities of occurrence > 0.10 (Figs. S15a–c, e–g).

## Discussion

### Phylogeny of *Cherleria*: comparison of nrDNA and GBS data

With respect to interspecific phylogenetic relationships, the GBS results presented here were largely congruent with the nrDNA results obtained in an earlier analysis [55], despite the fact that the GBS data integrate over a much larger portion of the genome and that each individual locus is much less informative (Table 2). However, the GBS data also show that the evolution of *Cherleria* was more complicated than inferred from nrDNA data, with several instances of gene flow. The possible evolutionary significance of gene flow will be discussed in detail below.

The present study included all members of Clade C (the Alpine/Balkan clade) of our previous analysis based on nrDNA sequence data [55], Fig. 2) except for *C. handelii* Mattf., for which we could not obtain fresh material. In the nrDNA data, all species for which multiple individuals were sampled were resolved as monophyletic, except for *C. capillacea*, in which the Albanian population was separate from the remaining populations. The nrDNA data grouped the plants sampled here into three subclades: a subclade of calcifuge species (*C. baldaccii*, *C. garckeana*, *C. laricifolia*), a subclade of calcicole species (*C. capillacea*, *C. handelii*, *C. langii*), and a subclade containing three narrow endemics from Greece (*C. dirphya*, *C. parnonia*, *C. wettsteinii*). The two remaining species, *C. doerfleri* and *C. sedoides*, had uncertain positions within Clade C. *Cherleria rupestris* (*M. labillardierei* Briq.), the outgroup in this study, was outside of Clade C in the nrDNA tree.

Both tree (Figs. 3, S1, S2, S3, S4, S5) and network (Figs. S6, S7) analyses of the GBS data also showed most species to be monophyletic. The exceptions were *C. capillacea* and *C. baldaccii*. In *C. capillacea*, the Albanian population again grouped separately from the rest. The type locality of *C. capillacea* is the Col de Tende, on the border between France and Italy in the western Alps [3]. Thus, when the species is split, the name *C. capillacea* belongs with the western/northern populations. Therefore, core *C. capillacea*, represented by three French populations in the present study, but also including samples from Italy and Bosnia-Herzegovina in the nrDNA analyses [55], will be referred to henceforth as *C. capillacea* s.s. The other group will be referred to as Albanian *C. capillacea*, even though it is possible that the range of that species extends outside of Albania. The boundary between *C. capillacea* s.s. and Albanian *C. capillacea* remains unclear, due to limited sampling. In *C. baldaccii*, the two populations sampled in this study were sometimes not sister to each other (Additional file 4:Fig. S5), but monophyly of the species was also not consistently supported in the nrDNA analysis [55].

In *C. langii* and *C. laricifolia*, intraspecific relationships found here differ somewhat from those found by Moore & Kadereit [55]. In *C. langii*, the present analysis revealed more intraspecific phylogenetic structure than our earlier nrDNA analysis [55]. Although there were no strongly supported groups within this species in the phylogenetic analyses of the GBS data, the plants were divided into Austrian populations (from the Alps) and Slovakian populations (from the Carpathians) in *adegenet* (Figs. 4a, Additional file 4: S9) and *fineRADstructure* (Additional file 4: Additional file 4: Fig. S12). Within *C. laricifolia*, the populations from the Pyrenees and the Massif Central strongly grouped together in SVDQuartets (Figs. 3, Additional file 4: S5), SplitsTree (Additional file 4: Fig. S7), *adegenet* (Figs. 4b, Additional file 4: S10), and *fineRADstructure* (Additional file 4: Fig. S11). Relationships of this group to the remainder of the species differed among analyses. These populations were separated as *C. laricifolia* subsp. *diomedis* (Braun-Blanquet) A.J. Moore & Dillenb., due to the presence of glandular hairs. However, this character is somewhat variable within populations (L. Sáez Goñalons, Universidad Autónoma de Barcelona, personal communications), and this group is often included within subsp. *laricifolia* [34]. Subspecies *ophiolitica* also formed its own group in many of the *adegenet* runs (Figs. 4b, Additional file 4: S10) and in *fineRADstructure* (Additional file 4: Fig. S11). When it was not a separate group, it grouped with the populations of subsp. *laricifolia* from the Maritime Alps and elsewhere in France, which is congruent with its sharing of chloroplast haplotypes with the Maritime Alps populations of subsp. *laricifolia* [56]. The Alpine populations of subsp. *laricifolia* were also sometimes divided into Western Alps and Central Alps groups in the *adegenet* analyses (Fig. 4b), congruent with our previous AFLP results [56].

Of the major clades found with the nrDNA data, the GBS data also resolved a well-supported clade containing the three narrow endemics from Greece. The subclade of calcifuge species was also sometimes recovered, although one or both of the populations of *C. baldaccii* often grouped separately from the rest of the calcifuge clade. The two sampled species from the calcicole subclade, *C. capillacea* and *C. langii*, never grouped together exclusively, in part because Albanian *C. capillacea* was separated from *C. capillacea* s.s. However, even without Albanian *C. capillacea*, the clade formed by *C. langii* and *C. capillacea* s.s. was never strongly supported. In contrast to the nrDNA data, in which the relationships among the subclades of Clade C were not resolved, the GBS data showed that the Greek endemics, the calcifuge species, and Albanian *C. capillacea* grouped together with strong support.

### Evolutionary significance of interspecific gene flow in *Cherleria*

Although the GBS data did not recover fundamentally different relationships from those found with the nrDNA data, several of our analyses point to interspecific hybridization in the evolutionary history of *Cherleria*. *Cherleria laricifolia* in particular (1) had low bootstrap support in SVDQuartets (Fig. 3) and the topology of the SVDQuartets tree differed from that of the RAxML tree of the GBS data (Additional file 4: Fig. S2). (2) The branch subtending *C. laricifolia* in the SplitsTree analyses was quite wide, indicating multiple divergent histories in the data, and the species was not supported in those analyses (Additional file 4: Fig. S7). (3) The group formed by *C. laricifolia* in both *fineRADstructure* (Fig. 5) and *adegenet* (Additional file 4: Fig. S8; Table 3) was always diffuse or poorly supported. (4) Most importantly, direct tests of hybridization using *D*_*FOIL*_ recovered evidence that several different species hybridized with *C. laricifolia*, namely *C. baldaccii*, *C. capillacea* s.s., *C. garckeana*, *C. parnonia*, and the common ancestor of *C. parnonia* and *C. wettsteinii* (Additional file 2). Although the species involved in hybridization with *C. laricifolia* were the same whether the SVDQuartets tree or the RAxML tree was used as the basis for choosing groups, the amount of hybridization inferred for each population of *C. laricifolia* varied in the two sets of analyses. These differences were likely due to the differences in topology between the two trees, with the most dramatic being that the Pyrenees/Massif Central populations were sister to the remainder of *C. laricifolia* in the SVDQuartets trees (Figs. 3, S5) and were nested within *C. laricifolia* in the RAxML tree (Additional file 4: Fig. S2).

In contrast to the situation with *C. laricifolia*, where low support for its monophyly indicated inconsistencies with a strictly branching scenario with no reticulation, *C. garckeana* and *C. langii* formed better supported, tighter groupings in all trees and analyses (Figs. 3, 5, S5, S7), and we would not have suspected hybridization in the absence of explicit tests for it. However, *D*_*FOIL*_ tests also detected hybridization between *C. garckeana* and *C. capillacea* s.s., *C. laricifolia*, *C. parnonia*, and the common ancestor of *C. parnonia* and *C. wettsteinii* and between *C. langii* and both *C. baldaccii* and *C. capillacea* s.s. (Additional file 2). Thus, detectable hybridization appears to take place both in parts of the phylogenetic tree where it could be predicted from features of the tree and in other parts where it could not be predicted, without explicitly testing for it. Assuming only a relatively small percentage of the loci were of hybrid origin, and the remaining loci were congruent in supporting a single topology because the group has otherwise had a relatively long independent history, then hybridization could still be evolutionarily significant, while having minimal impact on the reconstructed species tree. Similar scenarios have been found in South American siskins [12] and *Sceloporus* lizards [45].

Hybridization among closely related species is hypothesized to enlarge the gene pool of potentially adaptive loci and allow groups to undergo rapid morphological or ecological radiation or range expansion [1, 32, 51, 65, 90]. Examples of specific known genes that have been transferred by hybridization include genes for wing pattern in *Heliconius* [61], flower color in the *Diplacus aurantiacus* complex (formerly *Mimulus aurantiacus*; [79], serpentine tolerance in *Arabidopsis arenosa* [9], and winter coat color in various species of hares [31, 41]. In many other cases, hybridization is hypothesized to have allowed species to expand their ranges, although the genes that are responsible have not always been determined (e.g., *Cupressus* [47]; *Populus* [16, 82]).

Although the specific genes and adaptations involved for *Cherleria* are unknown, it is potentially important that of the species included in the current study, *C. garckeana*, *C. laricifolia*, and *C. sedoides* are the only ones with substrate polymorphism. *Cherleria garckeana* is an edaphic generalist and can be found on calcareous, siliceous, and serpentine substrates [43], with serpentine likely ancestral [55]. *Cherleria laricifolia* is largely found on siliceous substrates, except for the serpentine endemic subsp. *ophiolitica*, and is entirely absent from calcareous substrates. *Cherleria sedoides* is also an edaphic generalist. All of these species show evidence of hybridization, with *C. sedoides* previously shown to have undergone hybridization with a species that is now extinct [57]. At least *C. garckeana* and *C. laricifolia* appear to have hybridized with species that are restricted to various different substrates and from which this expansion of edaphic niche might have been obtained. In addition, two of the species with the largest ranges, *C. laricifolia* (this study) and *C. sedoides* [57], showed varying levels of hybridization in different populations or parts of their ranges, potentially indicating that the genes acquired via hybridization were more important in some parts of their ranges than in others. The remaining widespread species, *C. capillacea* s.s., was not sampled well enough in this study to be able to determine the extent of hybridization.

### Hybridization as evidence for past distribution

In addition to the evolutionary implications of hybridization or introgression, past hybridization can shed light on the biogeographic history of a group [44]. In some cases, phylogenetic or phylogeographic reconstruction is congruent with hybridization, and shows that the hybridizing lineages would have been in the same place at the same time ([13, 16, 91]. However, in other cases, the parents of the hybrid do not have overlapping ranges today ([27, 63, 86, 87]. For these species, broader ranges in the past or long-distance dispersal followed by extinction likely led to hybridization. For example, New World tetraploid *Gossypium* is an allopolyploid hybrid between a New World diploid and an Old World diploid that apparently dispersed to the New World and went extinct after the hybridization event [87].

We find both scenarios in *Cherleria*. The range of *C. garckeana* currently overlaps or is adjacent to the ranges of most of the species with which it has hybridized. Although the contemporary ranges of *C. langii* and *C. capillacea* s.s. do not overlap, we detected hybridization between these two species. It is possible that past contact between the eastern-most populations of *C. capillacea* s.s. and the western-most populations of *C. langii* occurred. However, matters are different in *C. laricifolia*. Its range does overlap with that of *C. capillacea*, but the other species with which it has apparently hybridized are restricted to the southern Balkan Peninsula where it does not grow today. We therefore hypothesize that *C. laricifolia* once was more widespread and probably originated on the Balkan Peninsula from whence it colonized the Alps and western Europe. Interestingly, distribution modeling does not give high support for a more widespread southern range of *C. laricifolia* around the Mediterranean. However, it has been shown that selection takes place during migration [15], and, thus, the plants that were able to grow along the Mediterranean were likely genetically and ecologically different from the ones that are now growing in the current range of *C. laricifolia*. A past distribution of *C. laricifolia* in southern areas is also supported by the high genetic diversity and lack of distinctiveness of *C. laricifolia* subsp. *ophiolitica*, which is currently disjunct from the rest of the species in the northern Apennines in Italy [56].

## Conclusions

The nrDNA phylogeny does appear to represent the dominant history of these *Cherleria* species, as it is largely congruent with phylogenies derived from GBS data. However, hybridization was also important in the evolution of the group. Although the specific genes transferred by hybridization are unclear, it appears that there has been extensive hybridization between *C. laricifolia* and various Balkan species, as well as hybridization involving both *C. garckeana* and *C. langii*. This hybridization shows that the ranges of some of these species must have been more extensive at one point, with *C. laricifolia* being found on the Balkan Peninsula and *C. capillacea* and *C. langii* overlapping.

It is generally expected that the use of the increasingly large amounts of data available through phylogenomic methods will eventually yield fully resolved, well-supported phylogenetic trees from even the most recalcitrant groups. However, as we show here, although each individual analysis may recover a relatively well-supported set of relationships, conflict between analyses of the same data set often remains. Instead of considering these conflicts to be noise that distract from understanding the evolution of the group, it is possible to gain new insights into the group, beyond what a single species tree would indicate. As shown here, evidence of past hybridization can both provide clues as to how the group was able to colonize new niches and can serve as an indicator of past distribution ranges.

## Methods

### Sample preparation and sequencing

All species of Clade C [55], corresponding to *Minuartia* section *Spectabiles* (Fenzl) Hayek subsection *Laricifoliae* (Mattf.) McNeill series *Laricifoliae* of McNeill [52] with the addition of *Cherleria sedoides*) were sequenced, with the exception of *C. handelii*, of which no fresh or silica-dried material was available. In addition, *C. rupestris* (*M. labillardierei*)*,* which formed part of a polytomy with Clade A and Clades B + C in Moore and Kadereit’s [55] phylogeny, was included as an outgroup. In total, 286 individuals from 63 populations were sequenced (Fig. 2, Table 1), with five individuals per population, when the populations consisted of at least five individuals. (At all of the sampled localities, the plants occur in groups of relatively few individuals (tens to hundreds) that are isolated from other groups of individuals of the same species (Moore, unpublished observations). Thus, plants from one sampling locality can be assumed to be members of a single, interbreeding population that experiences very low to no gene flow from other such populations.) Sampling was concentrated on *C. laricifolia* (130 individuals from 27 populations) and *C. langii* (80 individuals from 16 populations) with individuals sampled throughout the ranges of these species. Although the remaining species were sampled at a much lower density, an attempt was made to sample from as much as possible of their ranges.

In order to compare the GBS results with the nrDNA results of our previous study [55], RAxML v. 8.2.4 [77] was used to reconstruct the maximum likelihood tree from ITS and ETS sequence data, using the GTRCAT model of sequence evolution with 500 bootstrap replicates. The pruned alignment from Moore and Kadereit [55] was used, with the addition of the remaining populations that were not included in that study. PCR and sequencing were performed as described in Moore and Kadereit [55]. One sequence per population sampled in the current study was included, with the exception of *C. wettsteinii*, for which we had only a single sequence. GenBank numbers are given in Additional file 3. All map figures were made in R [67] using the packages *maps* and *mapdata*.

DNA was extracted from silica-dried leaf material using the Qiagen DNeasy Plant Mini Kit (Qiagen GmbH, Hilden, Germany). Approximately 0.015 g of dried leaf material was ground in a Retsch MM 301 mill (Retsch GmbH, Haan, Germany) and eluted twice in 50 μl AE (elution) buffer.

Libraries were prepared for Genotyping by Sequencing (GBS) following the protocol of Elshire et al. [24], as modified by Dillenberger and Kadereit [22]. Twenty-five different inline barcodes were used (the numbers 3, 5, 6, 8, 9, 11, 12, 14, 17, 19, 25, 30, 35, 36, 37, 38, 39, 41, 57, 60, 61, 69, 78, 79, and 81 from Elshire et al. [24] and combined with different third-read barcodes (TS01, TS04, TS05, TS06, and TS07) for multiplexing. The restriction enzyme *BamH*I was used, as we were collaborating with zoologists, and this was the enzyme that worked best for the two groups of organisms. Primers were obtained from Eurofins Genomics (Ebersberg bei München, Germany) and Sigma-Aldrich (Sigma-Aldrich Chemie GmbH, München, Germany) and all other reagents were obtained from New England Biolabs (New England Biolabs GmbH, Frankfurt am Main, Germany). PCR products were gel-extracted to remove adapters prior to sequencing using the NucleoSpin Gel and PCR Clean-Up kit (Macherey–Nagel GmbH & Co. KG, Düren, Germany). Pooled sets of 25 samples were sequenced on an Illumina HiSeq 2000 (Illumina, Inc., San Diego, California, USA) at the Institut für Organismische und Molekulare Evolutionsbiologie, Johannes-Gutenberg Universität Mainz, Germany. Each set of 25 samples was sequenced on approximately 0.20 lane. NCBI SRA accession numbers for each individual are given in Additional file 3.

### Read processing and grouping into loci

Processing of sequence data was carried out using the RTD pipeline [64] as modified using custom python scripts (found at https://github.com/abigail-Moore/GBS-analysis/), as described by Dillenberger and Kadereit [22]. A full description of the individual scripts is found in the ReadMe file in the git repository. Only 25 individuals, representative of the group’s phylogenetic diversity, were run through the RTD pipeline. The remaining individuals were added to the dataset by performing a BLASTn search [4, 14] against the set of loci found using the RTD pipeline and our custom scripts [22]. This two-step analysis protocol allowed us to add individuals relatively quickly, without having to reanalyze the whole dataset. It also allowed us to perform the required analyses on a desktop computer with 16 GB of RAM.

Initial alignments of each locus were produced containing all individuals. These alignments were then filtered in various ways to produce working alignments. Extremely variable loci (those with a variability more than three interquartile ranges above mean variability) were removed, as they likely represented multiple locations in the genome. Each working alignment was produced from the full alignments by removing individuals that had fewer than 10% of the loci and loci that were present in fewer than 70% of the individuals. There is a tradeoff between sampling breadth (number of taxa) and sampling depth (number of loci), as many loci are only present in certain taxa, due to losses of restriction sites or large indels. Therefore, different working alignments were produced for each subset of taxa, when they were analyzed separately, instead of simply pruning one master working alignment that contained all individuals with sufficient coverage.

For each subset of taxa, three different working alignments were produced: The Individual data set contained sequences from all individuals that worked well. The Population data set contained sequences from the single best individual from each population. The Taxon data set contained sequences from the best individual from each taxon, with the Albanian population of *C. capillacea* separated as its own taxon. In cases in which the best individual did not have a sequence for a particular locus, while another member of the same population did have a sequence for that locus, this sequence was used instead (see Table 1 for the populations sampled in this dataset).

The working alignments were then reformatted for the different analyses. For SNP-based analyses, if a given site had more than two alleles, due to higher ploidy, sequencing errors, or other polymorphisms, two were chosen at random. For sequence-based analyses, one sequence per individual per locus was chosen at random, to allow a concatenated alignment to be made. Preliminary analyses with different randomly chosen alleles or sequences did not show significantly different results, so only one set of analyses is presented here. Bootstrap alignments were made by resampling loci, instead of by resampling sites.

All alignments and trees have been deposited on Dryad [58], https://doi.org/10.5061/dryad.47d7wm397).

### Sequence-based analyses

In order to reconstruct the phylogeny while explicitly taking incomplete lineage sorting (ILS) into account, SVDQuartets [17, 18] was used, as implemented in PAUP* version 4.0a147 [81] (Swofford, continuously updated). Unlike most species-tree reconstruction programs, SVDQuartets models ILS using SNP data instead of requiring well-resolved and completely sampled gene trees. In the analyses, each individual can be a separate tip, or individuals can be grouped by species (or population) to better reconstruct ILS, and species (or population) trees can be reconstructed. Both population (one sequence per terminal) and species (multiple sequences per terminal) trees were reconstructed for the Population dataset, while population and species trees (both with multiple sequences per terminal) were reconstructed for the Individual dataset. For each analysis, a random set of 100,000 quartets was evaluated and 1000 bootstrap replicates were performed.

RAxML was used with the GTRCAT model of sequence evolution in an analysis of Population dataset. Branch support was assessed with 500 bootstrap replicates. The SNP model was not used, as the entire sequences were used for phylogenetic reconstruction, instead of using a dataset composed only of SNPs.

*SplitsTree* version 4.13.1 (built 16 April 2013; [38] was used to construct phylogenetic networks using the Taxon and Population datasets. The NeighborNet algorithm was used, with uncorrected p distances, which were necessary because the large amount of missing data made other distance estimates less reliable. Branch support was assessed with 1000 bootstrap replicates.

### SNP-based analyses

Principal components analysis and discriminant analysis of principal components (DAPC) were performed using *adegenet* [39, 40] in R [67]. For DAPC, in the initial clustering (*find.clusters* function), 50 principal components were used and the maximum number of clusters was set to twice the number of populations. The number of clusters was chosen based on the Bayesian Information Criterion (BIC) values, with ten replicate DAPC analyses run for each number of clusters. For the DAPC analysis of the clustered data (*dapc* function), the analysis was run initially with a large number of principal components, and the optimal number was chosen for the final analysis based on a-score optimization (*optim.a.score* function). All discriminant functions (the number of clusters minus one) were used in the final analysis. Individuals were considered admixed if their posterior probability of being assigned to any one cluster was less than 0.9. In addition, the a-scores of each cluster were calculated (*a.score* function).

For the DAPC analysis of all individuals (the entire Individual dataset), the lowest BIC values were found when the plants were divided into 18 clusters. However, the groupings at that level were too fine to be useful, and the largest drops in BIC values occurred for each increase in the number of clusters from one through seven. For increases between seven and 18 clusters, although the BIC values continued to decrease, the decreases were much smaller. Therefore, analyses were run with the plants divided into between two and seven clusters. The optimal number of principal components was twelve for almost all analyses of the Individual dataset, with 13 being optimal in some replicates.

For the DAPC analysis of *C. laricifolia* alone, the solution with the lowest BIC value was when the plants were divided into four groups. Analyses were run with the plants divided into two, three, and four groups. Using 10 principal components was optimal for most replicates, with nine principal components being optimal for six replicates. However, when only nine principal components were used, many individuals were not accurately classified into any of the clusters, so the results are based on analyses with 10 principal components.

For the DAPC analysis of *C. langii* alone, the solution with the lowest BIC value was when the plants were divided into two groups. Analyses were run with the plants divided into both two and three groups. Eight principal components were optimal in all but one replicate, in which case nine were optimal. The number of principal components used did not change the results.

We used *fineRADstructure* [48], which implements *fineStructure* [46] for RADseq data, as another way of looking at genetic groupings. We had unmapped data, so used the tag haplotype matrix input format. We were not able to correct for linkage disequilibrium using their *sampleLD.R* script, as it did not accept this input format. Default settings were used for *RADpainter*, while *finestructure* was run with 100,000 burnin iterations and 100,000 sample iterations, with sampling every 1000 iterations for clustering and default settings with 10,000 burnin iterations for tree building. Results were visualized using *fineRADstructure.R* using R. In all cases, all individuals from each group were analyzed.

Hybridization was assessed with the exhaustive D-statistic test using the Ex*D*_FOIL_ wrapper scripts [45] for the *D*_FOIL_ scripts [62]. Given the differences in topology between the trees from the SVDQuartets analysis and the RAxML analysis, two different trees were used to construct the five-taxon phylogenies: the SVDQuartets Population tree reconstructed with the Individual dataset and a RAxML tree reconstructed using the Population dataset. Trees were made ultrametric using TreePL [74]. In the RAxML tree, all branch lengths (internal and external) were reconstructed from the data. In the SVDQuartets tree, only the internal branch lengths were reconstructed from the data, with the terminal branches all of equal length. Given the topological differences between the RAxML and SVDQuartets trees and the fact that the terminal branches were all relatively long and approximately equal in length in the RAxML tree, we decided that making the SVDQuartets tree itself ultrametric was more accurate than using its topology as a constraint with branch lengths reconstructed in RAxML. The initial run of Ex*D*_*FOIL*_ was conducted using the *prime* option to find the optimal analysis parameters, which were then used in the final run. *Cherleria rupestris* was used as the outgroup in both TreePL and Ex*D*_FOIL_, and all 205,457 comparisons were tested. Initially, the number of tests that reconstructed introgression between each of the species was used, and species with more than 50 predicted introgression events were examined in detail, to determine the amount of introgression between the various populations.

### Species distribution modeling

Herbarium collections of *Cherleria capillacea*, *C. langii*, and *C. laricifolia* from B, BM, BRU, E, F, K, M, MJG, MSB, NY, W, WU, Z, and ZT were georeferenced. These specimens were either obtained on loan (E, F, M, JSB, W, WU, Z, and ZT) or locality information was recorded during herbarium visits after specimen identity was verified (B, BM, BRU, E, K, MJG, NY, W, WU). All localities visited in the course of our work were also included (vouchers housed in MJG). In a few cases, data were also obtained from the Global Biodiversity Information Facility ([28], before a doi was assigned, citations for individual datasets are as follows: *Cherleria capillacea*: Phanerogamie, http://data.gbif.org/datasets/resource/1506; Herbario de la Universidad de Sevilla, SEV, http://data.gbif.org/datasets/resource/283; Royal Botanic Gardens, Kew, http://data.gbif.org/datasets/resource/629; Biological and palaeontological collection and observation data MNHNL, http://data.gbif.org/datasets/resource/8107; Inventaire national du Patrimoine naturel (INPN), http://data.gbif.org/datasets/resource/2620. *Cherleria laricifolia*: Institut Botanic de Barcelona, BC, http://data.gbif.org/datasets/resource/299; Fundación Biodiversidad, Real Jardín Botánico (CSIC): Anthos. Sistema de Información de las plantas de España, http://data.gbif.org/datasets/resource/9090; Herbarium GJO, http://data.gbif.org/datasets/resource/1484; Herbario de la Universidad de Salamanca: SALA, http://data.gbif.org/datasets/resource/239; SANT herbarium vascular plant collection, http://data.gbif.org/datasets/resource/222) for the cases when only a single species of *Cherleria* grew in that area.

All records lacking coordinates were georeferenced using a combination of maps of the areas in question, Google Earth Pro (versions 7.3.2.5776 and earlier, Google LLC, Mountain View, CA), and OpenStreetMap (www.openstreetmap.org). Only records that could be georeferenced to within 1000 m were used and exact duplicate records were eliminated. In total, we were left with 57 records for *C. capillacea*, 55 records for *C. langii*, and 229 records for *C. laricifolia*. The records from *C. laricifolia* subspp. *laricifolia* and *ophiolitica* were combined because subsp. *ophiolitica* is nested within subsp. *laricifolia* and because there were too few records of subsp. *ophiolitica* to analyze alone. Due to a relatively small number of available data points, all available localities were used, regardless of year. The ages of the localities ranged from 1836 to 2012 (*C. capillacea*), 1844–2011 (*C. langii*), and 1823–2012 (*C. laricifolia*), although some specimens did not have collection dates and could have been older than these ranges. These records were exclusively from the native range of the plants, as they are not known to be introduced anywhere. All records were within the known ranges of the species.

The analyses were carried out over an area including all countries in which the three species of *Cherleria* occur plus Bulgaria, the Czech Republic, Germany, Moldova, and the Ukraine, as these adjacent countries also contained potentially suitable mountain habitat. All islands were excluded with the exception of Corsica, Crete, Mallorca, Sardinia, and Sicily. All data layers were clipped to this extent and the grid size was rescaled to 1 km square (X, Y cell size of 0.0083333333, 0.0083333333), to be compatible with the BioClim dataset. Clipping and rescaling was carried out in ArcMap 10.4.1 (ESRI, Redlands, CA).

For modeling the current distribution, the BioClim variables derived from the WorldClim 2 dataset were used [26], together with a Europe-wide dataset of soil pH [92]. For past climates, we used the bioclimatic variables from all three models that were available for the Last Glacial Maximum (LGM): CCSM4 [29], MIROC-ESM [85], and MPI-ESM-P [30], all calibrated using WorldClim 1.4.

Preliminary analyses for the three different groups were carried out with all of the variables. In addition, an analysis of correlation of the BioClim variables over the study area was carried out in ArcMap. The variables chosen for the final set of analyses were based on two factors: their importance in the preliminary analyses and their lack of correlation with other variables included in the analysis. Only variables with a correlation coefficient of < 0.70 were retained. Different variables were retained for the different species.

MaxEnt version 3.4.1 [66] was used to create all species distribution models (SDMs). Variable importance was measured by jackknifing. The models were run with 10,000 background points, 15 replicates, and 5000 iterations. The replicated run type was subsampled, due to low numbers of points for two of the three species, and all samples were added to the background. The models were developed with 80% of the data and tested with the remaining 20%. Models were evaluated using the area under the receiver operator characteristic (ROC) curve (AUC values). Separate runs were conducted for each of the three species with each of the three LGM models and both with and without pH data, for a total of two different reconstructions for current range and six different reconstructions of LGM range per species.

## Supplementary information

**Additional file 1.** Number of reads per individual. Detailed information on each individual including batch, total number of reads, number of unique reads, and whether they were excluded from the final analysis due to insufficient read depth.

**Additional file 2.** Proportion of exhaustive D-statistic tests showing hybridization between different groups of populations. Tests were based on the topology of the SVD-Quartets (SVDQ, Fig. S5) and RAxML (RAxML, Fig. S1) trees based on the Population dataset. Population codes follow Table 1. Taxon codes are as follows: BA: C. baldaccii, CA: C. capillacea, GA: C. garckeana, LL: C. laricifolia subsp. laricifolia, LN: C. langii, LO: C. laricifolia subsp. ophiolitica, PA: C. parnonia, and WE: C. wettsteinii. No results were shown for C. dirphya, C. doerfleri, C. rupestris, or C. sedoides, as < 5% of these tests were significant in all cases.

**Additional file 3.** Voucher information and sequence accession numbers. Detailed locality information for each collection, GenBank accession numbers for nrDNA ITS and ETS sequences, and NCBI SRA accession numbers for raw GBS data.

**Additional file 4: Fig. S1.** Comparison of the tree topologies from the different analyses. Bootstrap values > 70% are above the branches. In cases where the species were not forced to be monophyletic (S1a, S1b, S1c, S1f), bootstrap values for species are also shown. RAxML analysis of nrDNA ITS and ETS data (S1a, full tree in Fig. 2), population tree from the SVDQuartets analysis of the Individual dataset (S1b, full tree in Fig. 3), RAxML analysis of Population dataset (S1c, full tree in Fig. S2), species tree from SVDQuartets analysis of Population dataset (S1d, full tree in Fig. S3), species tree from SVDQuartets analysis of Individual dataset (S1e, full tree in Fig. S4), and population tree from SVDQuartets analysis of Population dataset (S1f, full tree in Fig. S5). Taxon abbreviations are as follows: BA, Cherleria baldaccii; BA231: population 231 of C. baldaccii; BA232: population 232 of C. baldaccii; CA: French populations of C. capillacea; CA233: Albanian population of C. capillacea; DI: C. dirphya; DO: C. doerfleri; GA: C. garckeana; LN: C. langii; LLO, C. laricifolia; PA: C. parnonia; RU: C. rupestris; SE: C. sedoides; and WE: C. wettsteinii. **Fig. S2.** Maximum likelihood phylogeny from the RAxML analysis of the Population dataset. Bootstrap values from 500 bootstrap replicates are above the branches; only values above 70% are shown. **Fig. S3.** Species tree from the SVDQuartets analysis of the Population dataset. Bootstrap values from 1000 bootstrap replicates are above the branches; only values above 70% are shown. **Fig. S4.** Species tree from the SVDQuartets analysis of the Individual dataset. Bootstrap values from 1000 bootstrap replicates are above the branches; only values above 70% are shown. **Fig. S5.** Population tree from SVDQuartets analysis of the Population dataset. Bootstrap values from 1000 bootstrap replicates are above the branches; only values above 70% are shown. **Fig. S6.** Network from the SplitsTree analysis of the Taxon dataset. Bootstrap values from 1000 bootstrap replicates are shown, together with lines indicating which taxa are involved; only values above 70% are shown. **Fig. S7.** Network from the SplitsTree analysis of the Population dataset. Bootstrap values from 1000 bootstrap replicates are shown, together with lines indicating which populations are involved; only values above 70% are shown. **Fig. S8.** Representative plots from the adegenet analyses of all plants combined. The plants were divided into two clusters (S8a), three clusters (S8b), four clusters (S8c), five clusters (S8d), six clusters (S8e), and seven clusters (S8f). **Fig. S9.** Representative plot from the adegenet analysis of Cherleria langii alone. The plants were divided into two groups. **Fig. S10.** Representative plots from the adegenet analyses of Cherleria laricifolia alone. The plants were divided into two (S10a) and three (S10b) clusters. **Fig. S11.** Plot from the fineRADstructure analysis of Cherleria laricifolia alone. **Fig. S12.** Plot from the fineRADstructure analysis of Cherleria langii alone.

**Additional file 5: Fig. S13.** Modeled species distributions for Cherleria capillacea. Models were from MaxEnt based on BioClim variables, with or without pH included. Models with pH are projected onto the LGM climate reconstructed with the CCSM4 (S13a), MIROC-ESM (S13b), and MPI-ESM-P (S13c) climate models. Models without pH are the current distribution (S13d) and its projection onto LGM climate reconstructed using the CCSM4 (S13e), MIROC-ESM (S13f) and MPI-ESM-P (S13g) climate models. **Fig. S14.** Modeled species distributions for Cherleria langii. Models were from MaxEnt based on BioClim variables, with or without pH included. Models with pH are projected onto the LGM climate reconstructed with the CCSM4 (S14a), MIROC-ESM (S14b), and MPI-ESM-P (S14c) climate models. Models without pH are the current distribution (S14d) and its projection onto LGM climate reconstructed using the CCSM4 (S14e), MIROC-ESM (S14f) and MPI-ESM-P (S14g) climate models. **Fig. S15.** Modeled species distributions for Cherleria laricifolia. Models were from MaxEnt based on BioClim variables, with or without pH included. Models with pH are projected onto the LGM climate reconstructed with the CCSM4 (S15a), MIROC-ESM (S15b) and MPI-ESM-P (S15c) climate models. Models without pH are the current distribution (S15d) and its projection onto LGM climate reconstructed using the CCSM4 (S15e), MIROC-ESM (S15f) and MPI-ESM-P (S15g) climate models.

## Data Availability

All nrDNA sequence datasets are available in GenBank (https://www.ncbi.nlm.nih.gov/genbank/); see Additional file 3 for GenBank accession numbers for each sequence. Raw GBS data are available in the NCBI Sequence Read Archive (PRJNA613049, https://www.ncbi.nlm.nih.gov/sra/PRJNA613049); see Additional file 3 for NCBI SRA accession numbers for each individual. Scripts used to analyze GBS data are deposited on GitHub (https://github.com/abigail-Moore/GBS-analysis/). All additional data files, including sequence alignments, tree files, and R scripts are deposited on Dryad (http://dx.doi.org/10.5061/dryad.47d7wm397, [58].

## Abbreviations

AUC: Area under the receiver operator characteristic curve
cpDNA: Chloroplast DNA
EAS: European Alpine System
GBS: Genotyping by sequencing
ILS: Incomplete lineage sorting
LGM: Last glacial maximum
nrDNA: Nuclear ribosomal DNA
SDM: Species distribution model
SNP: Single nucleotide polymorphism
