# Supplementary material for: Range and niche expansion through multiple interspecific hybridization: a genotyping by sequencing analysis of Cherleria (Caryophyllaceae)
Source: BMC Ecol Evol. 2021 Mar 10;21:40. doi: 10.1186/s12862-020-01721-5 (PMC7945309; doi:10.1186/s12862-020-01721-5)
Supplement: Supplementary file 4 — Additional file 4: Fig. S1. Comparison of the tree topologies from the different analyses. Bootstrap values > 70% are above the branches. In cases where the species were not forced to be monophyletic (S1a, S1b, S1c, S1f), bootstrap values for species are also shown. RAxML analysis of nrDNA ITS and ETS data (S1a, full tree in Fig. 2), population tree from the SVDQuartets analysis of the Individual dataset (S1b, full tree in Fig. 3), RAxML analysis of Population dataset (S1c, full tree in Fig. S2), species tree from SVDQuartets analysis of Population dataset (S1d, full tree in Fig. S3), species tree from SVDQuartets analysis of Individual dataset (S1e, full tree in Fig. S4), and population tree from SVDQuartets analysis of Population dataset (S1f, full tree in Fig. S5). Taxon abbreviations are as follows: BA, Cherleria baldaccii; BA231: population 231 of C. baldaccii; BA232: population 232 of C. baldaccii; CA: French populations of C. capillacea; CA233: Albanian population of C. capillacea; DI: C. dirphya; DO: C. doerfleri; GA: C. garckeana; LN: C. langii; LLO, C. laricifolia; PA: C. parnonia; RU: C. rupestris; SE: C. sedoides; and WE: C. wettsteinii. Fig. S2. Maximum likelihood phylogeny from the RAxML analysis of the Population dataset. Bootstrap values from 500 bootstrap replicates are above the branches; only values above 70% are shown. Fig. S3. Species tree from the SVDQuartets analysis of the Population dataset. Bootstrap values from 1000 bootstrap replicates are above the branches; only values above 70% are shown. Fig. S4. Species tree from the SVDQuartets analysis of the Individual dataset. Bootstrap values from 1000 bootstrap replicates are above the branches; only values above 70% are shown. Fig. S5. Population tree from SVDQuartets analysis of the Population dataset. Bootstrap values from 1000 bootstrap replicates are above the branches; only values above 70% are shown. Fig. S6. Network from the SplitsTree analysis of the Taxon dataset. Bootstrap values fro [file 12862_2020_1721_MOESM4_ESM.pdf]

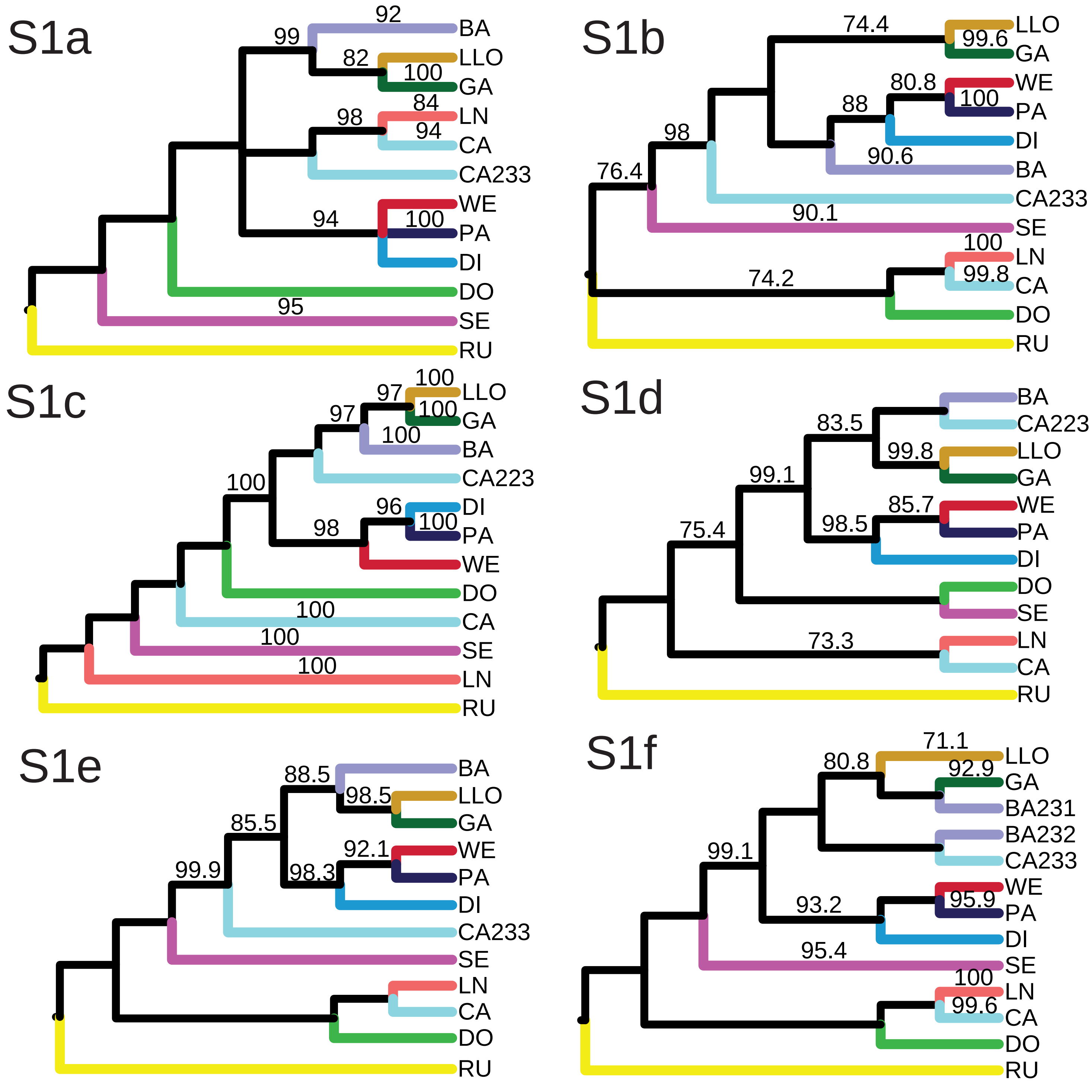

S1a
92
99
BA
82
LLO
100
GA
84
98
LN
94
CA
CA233
WE
94
100
PA
DI
DO
95
SE
RU
S1b
74.4
LLO
99.6
GA
80.8
WE
100
PA
88
DI
98
90.6
BA
76.4
CA233
90.1
SE
100
LN
74.2
99.8
CA
DO
RU
S1c
100
97
LLO
97
100
GA
100
BA
CA223
100
96
DI
98
100
PA
WE
DO
100
CA
100
SE
100
LN
RU
S1d
BA
83.5
CA223
99.8
LLO
99.1
GA
85.7
WE
98.5
PA
75.4
DI
DO
SE
73.3
LN
CA
RU
S1e
BA
88.5
98.5
LLO
85.5
GA
92.1
WE
99.9
98.3
PA
DI
CA233
SE
LN
CA
DO
RU
S1f
71.1
80.8
LLO
92.9
GA
BA231
BA232
99.1
CA233
WE
95.9
93.2
PA
DI
95.4
SE
100
LN
99.6
CA
DO
RU

Fig. S2

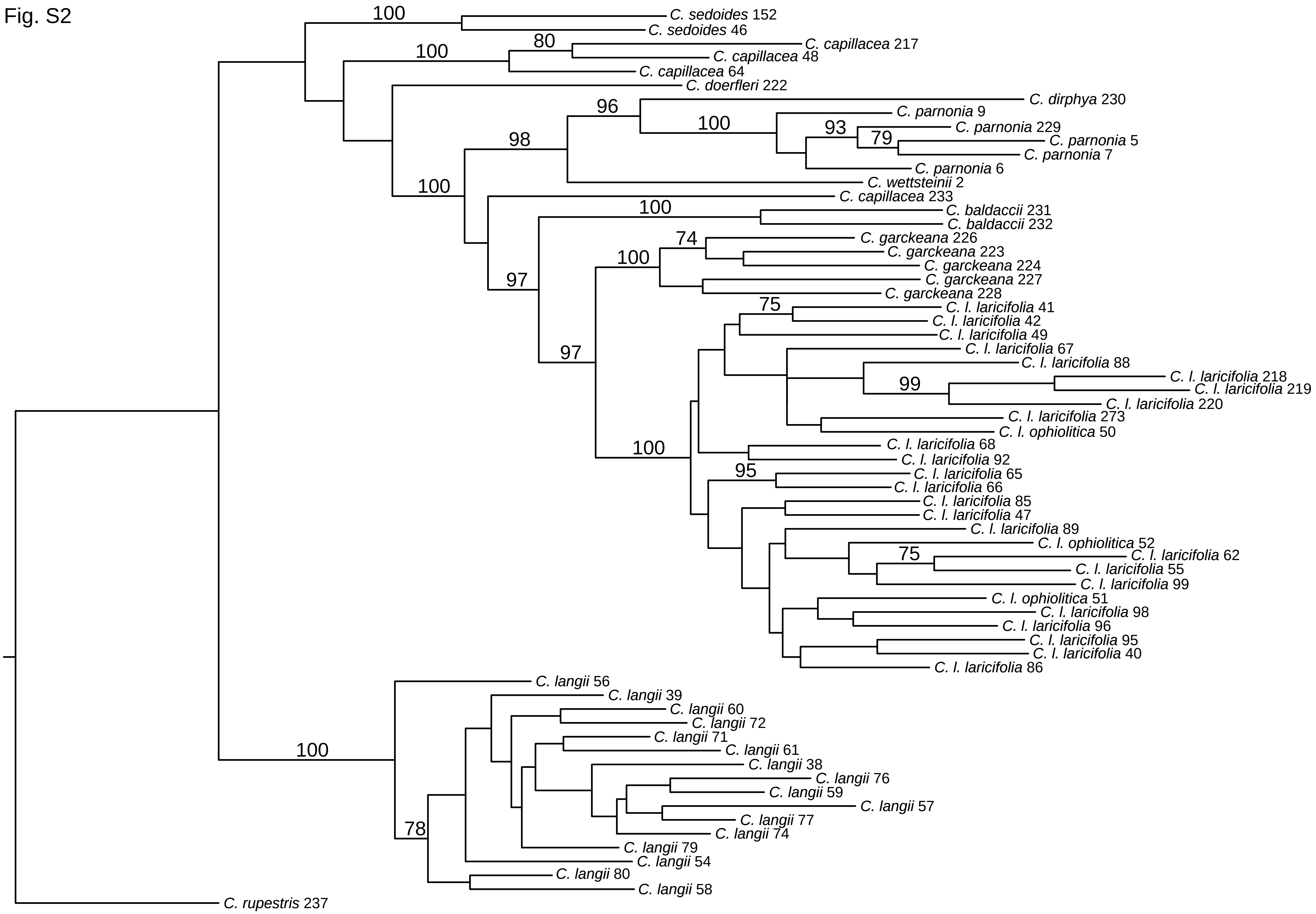

100
C. sedoides 152
C. sedoides 46
80
100
C. capillacea 217
C. capillacea 48
C. capillacea 64
C. doerfleri 222
96
C. dirphya 230
C. parnonia 9
100
93
C. parnonia 229
79
C. parnonia 5
C. parnonia 7
98
C. parnonia 6
C. wettsteinii 2
100
C. capillacea 233
100
C. baldaccii 231
C. baldaccii 232
74
C. garckeana 226
C. garckeana 223
100
C. garckeana 224
C. garckeana 227
97
C. garckeana 228
75
C. l. laricifolia 41
C. l. laricifolia 42
C. l. laricifolia 49
97
C. l. laricifolia 67
C. l. laricifolia 88
C. l. laricifolia 218
99
C. l. laricifolia 219
C. l. laricifolia 220
C. l. laricifolia 273
C. l. ophiolitica 50
C. l. laricifolia 68
100
C. l. laricifolia 92
95
C. l. laricifolia 65
C. l. laricifolia 66
C. l. laricifolia 85
C. l. laricifolia 47
C. l. laricifolia 89
C. l. ophiolitica 52
75
C. l. laricifolia 62
C. l. laricifolia 55
C. l. laricifolia 99
C. l. ophiolitica 51
C. l. laricifolia 98
C. l. laricifolia 96
C. l. laricifolia 95
C. l. laricifolia 40
C. l. laricifolia 86
C. langii 56
C. langii 39
C. langii 60
C. langii 72
C. langii 71
100
C. langii 61
C. langii 38
C. langii 76
C. langii 59
C. langii 57
C. langii 77
78
C. langii 74
C. langii 79
C. langii 54
C. langii 80
C. langii 58
C. rupestris 237

Fig. S4

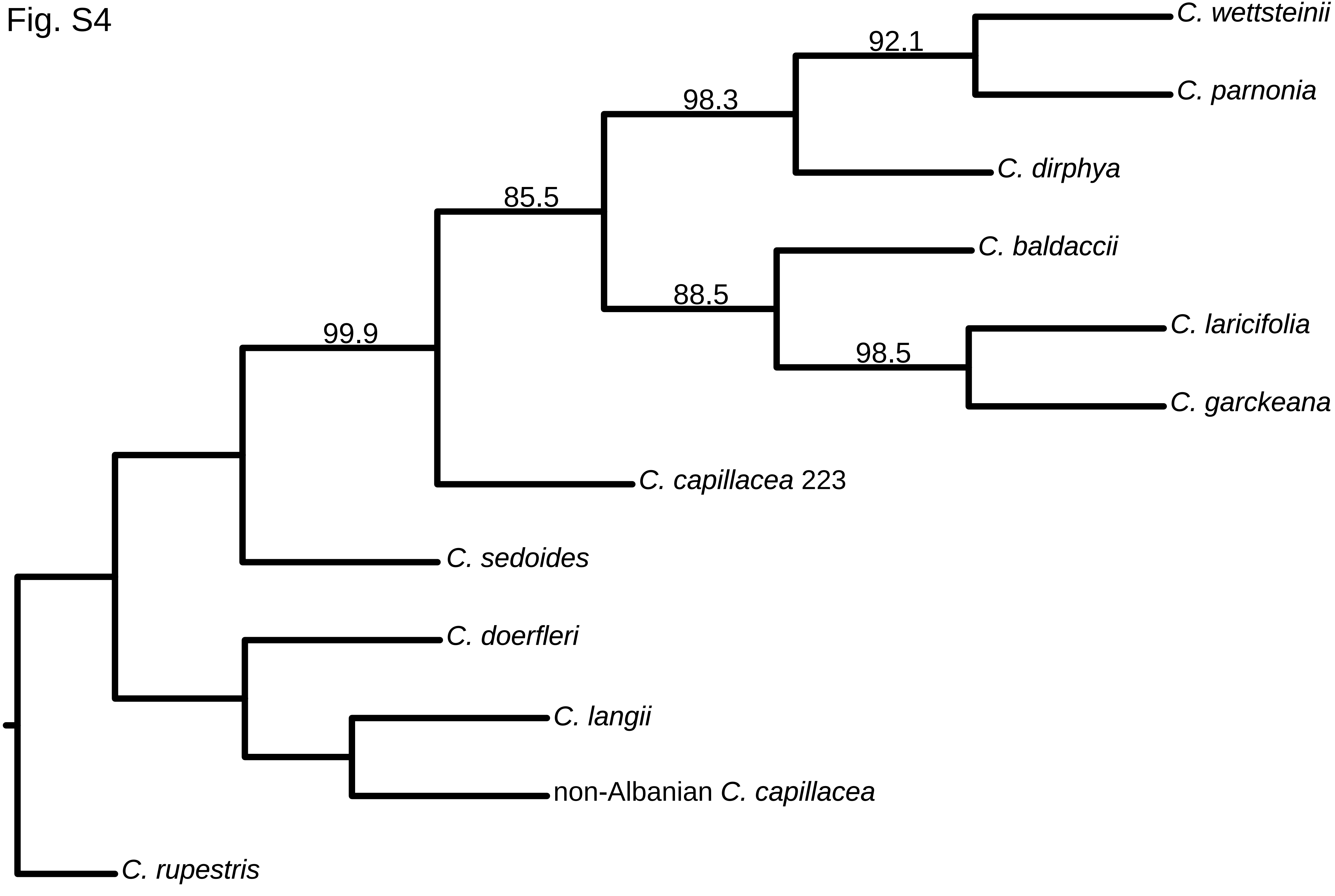
92.1
98.3
85.5
88.5
98.5
99.9
C. wettsteinii
C. parnonia
C. dirphya
C. baldaccii
C. laricifolia
C. garckeana
C. capillacea 223
C. sedoides
C. doerfleri
C. langii
non-Albanian C. capillacea
C. rupestris

Fig. S3

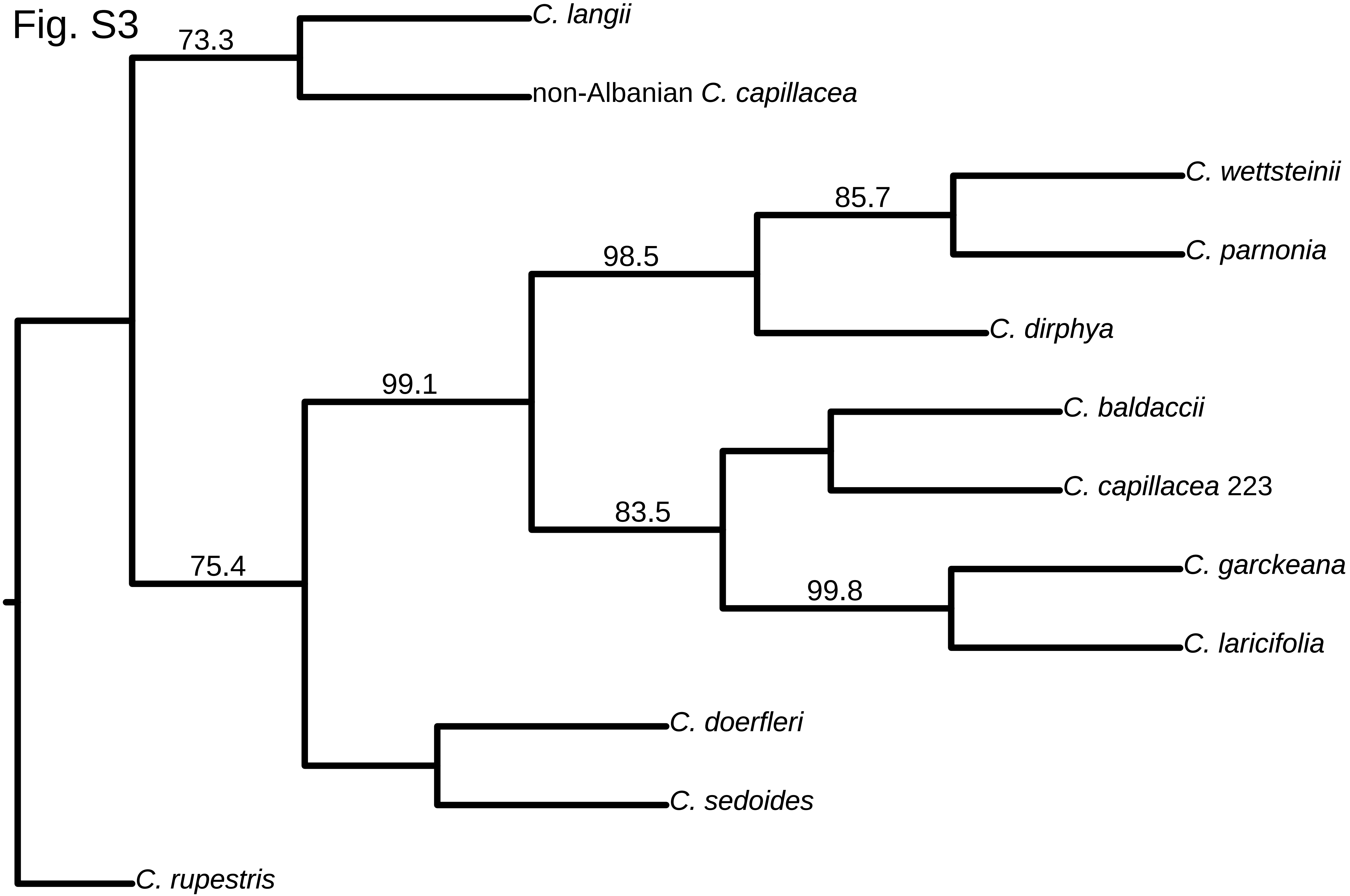

73.3
C. langii
non-Albanian C. capillacea
85.7
C. wettsteinii
C. parnonia
98.5
C. dirphya
99.1
C. baldaccii
C. capillacea 223
83.5
C. garckeana
75.4
99.8
C. laricifolia
C. doerfleri
C. sedoides
C. rupestris

Fig. S5

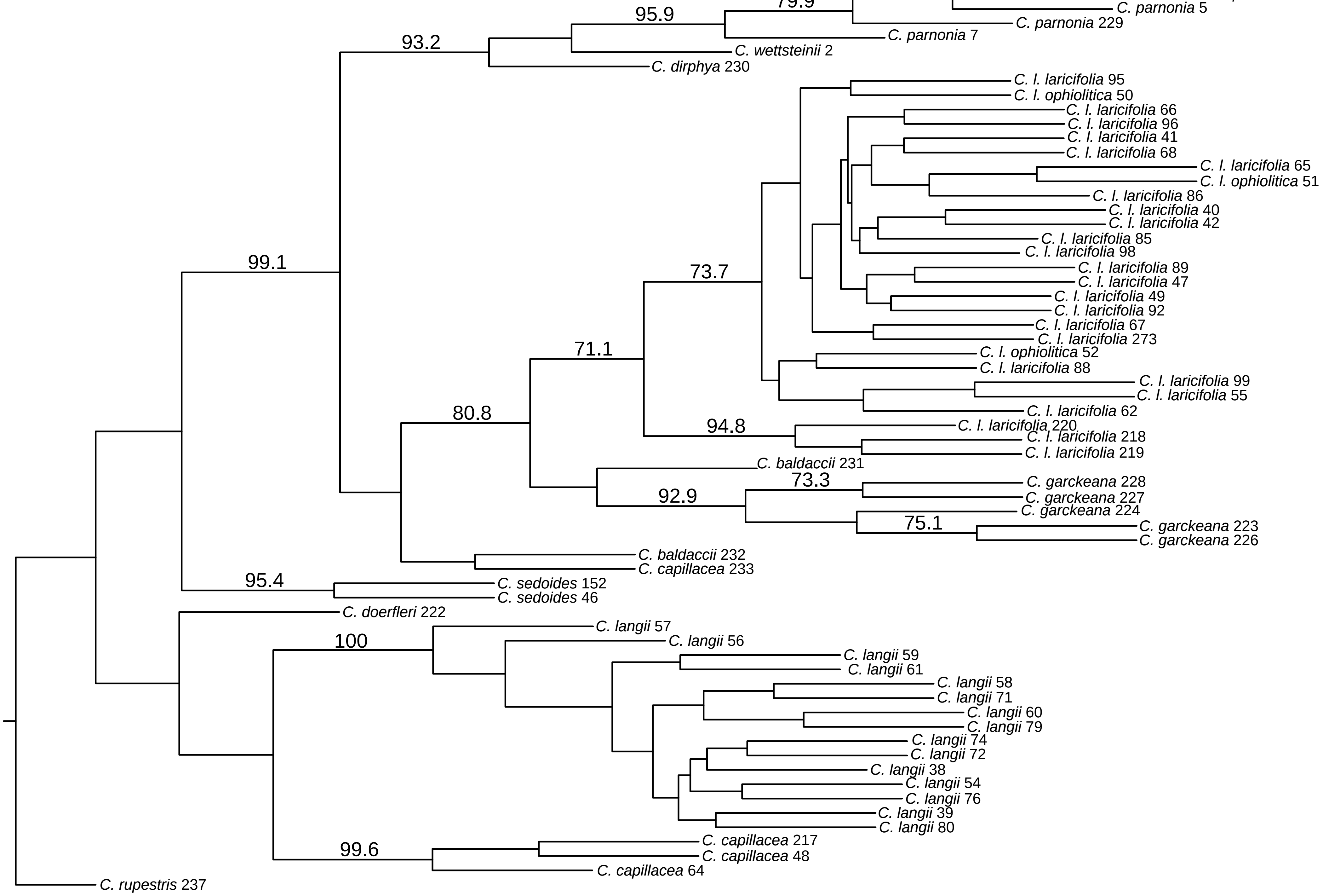

C. parnonia 6
C. parnonia 9
79.9
C. parnonia 5
95.9
C. parnonia 229
C. parnonia 7
93.2
C. wettsteinii 2
C. dirphya 230
C. l. laricifolia 95
C. l. ophiolitica 50
C. l. laricifolia 66
C. l. laricifolia 96
C. l. laricifolia 41
C. l. laricifolia 68
C. l. laricifolia 65
C. l. ophiolitica 51
C. l. laricifolia 86
C. l. laricifolia 40
C. l. laricifolia 42
C. l. laricifolia 85
C. l. laricifolia 98
99.1
C. l. laricifolia 89
73.7
C. l. laricifolia 47
C. l. laricifolia 49
C. l. laricifolia 92
C. l. laricifolia 67
C. l. laricifolia 273
71.1
C. l. ophiolitica 52
C. l. laricifolia 88
C. l. laricifolia 99
C. l. laricifolia 55
80.8
C. l. laricifolia 62
94.8
C. l. laricifolia 220
C. l. laricifolia 218
C. l. laricifolia 219
C. baldaccii 231
73.3
C. garckeana 228
92.9
C. garckeana 227
C. garckeana 224
75.1
C. garckeana 223
C. garckeana 226
C. baldaccii 232
C. capillacea 233
95.4
C. sedoides 152
C. sedoides 46
C. doerfleri 222
C. langii 57
100
C. langii 56
C. langii 59
C. langii 61
C. langii 58
C. langii 71
C. langii 60
C. langii 79
C. langii 74
C. langii 72
C. langii 38
C. langii 54
C. langii 76
C. langii 39
C. langii 80
C. capillacea 217
99.6
C. capillacea 48
C. capillacea 64
C. rupestris 237

Fig. S6

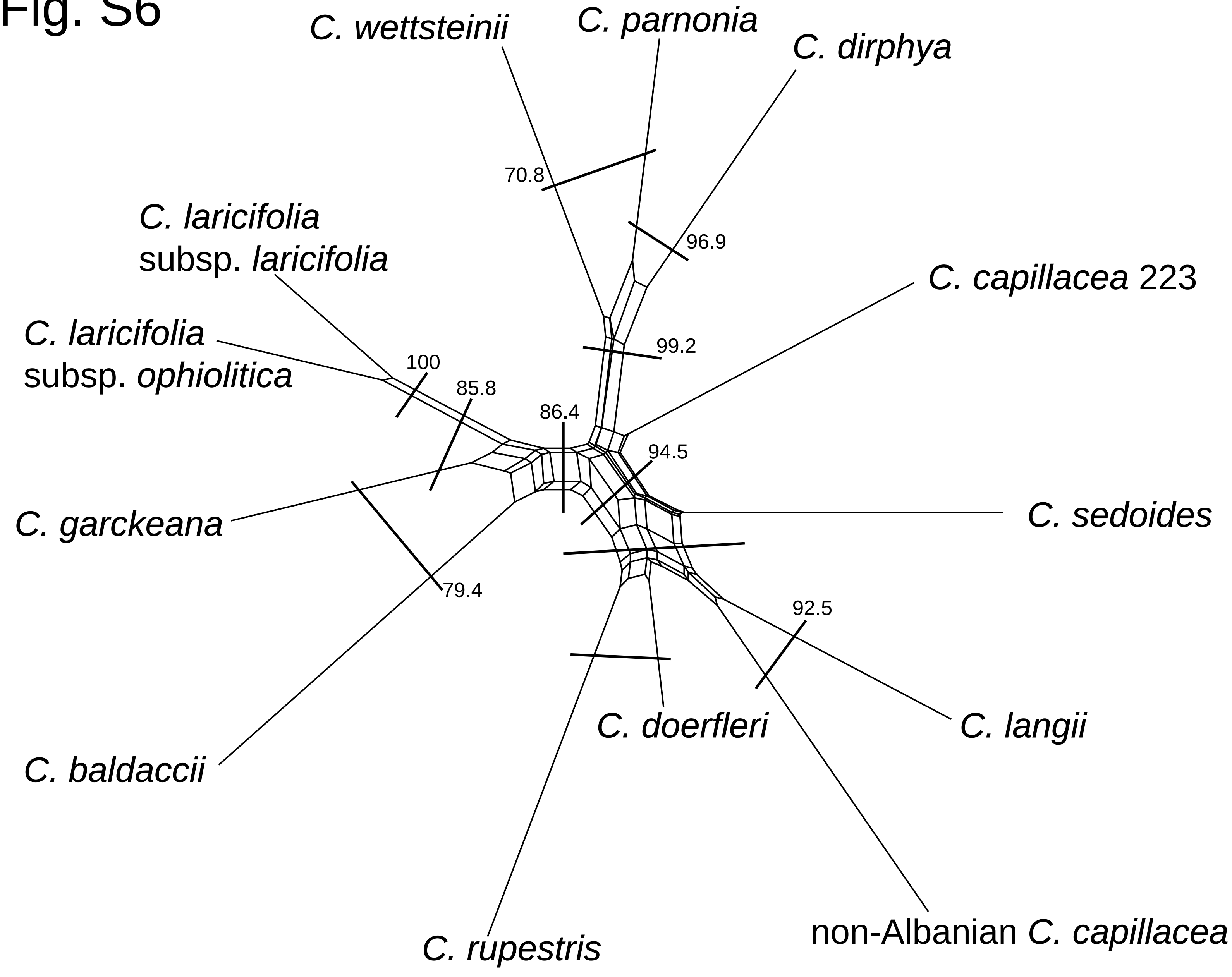
C. wettsteinii
C. parnonia
C. dirphya
70.8
96.9
C. laricifolia
subsp. laricifolia
C. capillacea 223
99.2
C. laricifolia
subsp. ophiolitica
100
85.8
86.4
94.5
C. sedoides
C. garckeana
79.4
92.5
C. doerfleri
C. langii
C. baldaccii
C. rupestris
non-Albanian C. capillacea

Fig. S7

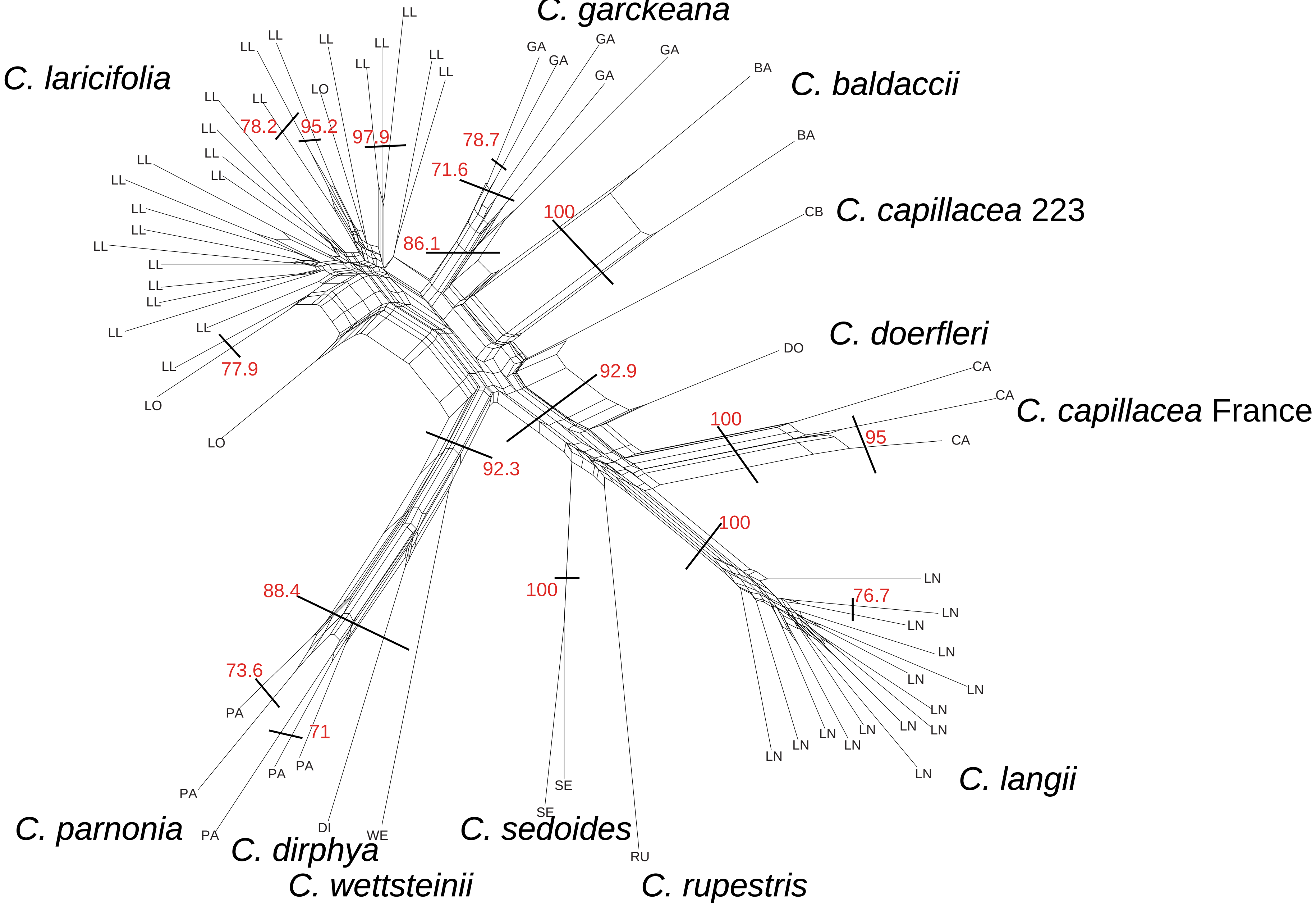
C. garckeana
C. laricifolia
C. baldaccii
C. capillacea 223
C. doerfleri
C. capillacea France
C. langii
C. parnonia
C. dirphya
C. wettsteinii
C. sedoides
C. rupestris
78.2
95.2
97.9
78.7
71.6
86.1
100
77.9
92.9
100
95
92.3
100
100
76.7
88.4
73.6
71
LL
LO
GA
BA
CB
DO
CA
LN
PA
DI
WE
SE
RU

Fig. S8a

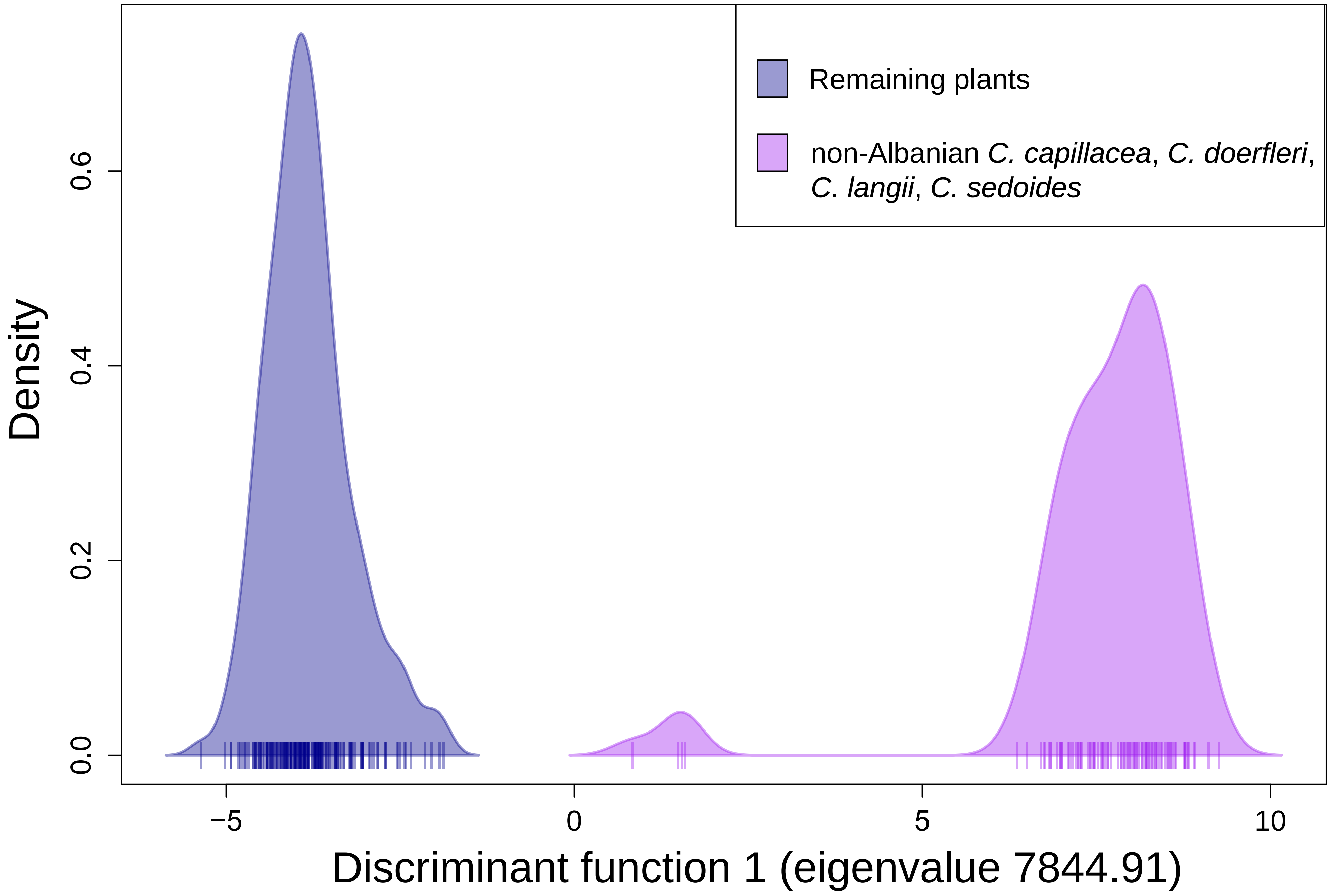
Remaining plants
non-Albanian *C. capillacea*, *C. doerfleri*, *C. langii*, *C. sedoides*
Density
0.0
0.2
0.4
0.6
−5
0
5
10
Discriminant function 1 (eigenvalue 7844.91)

Fig. S8b

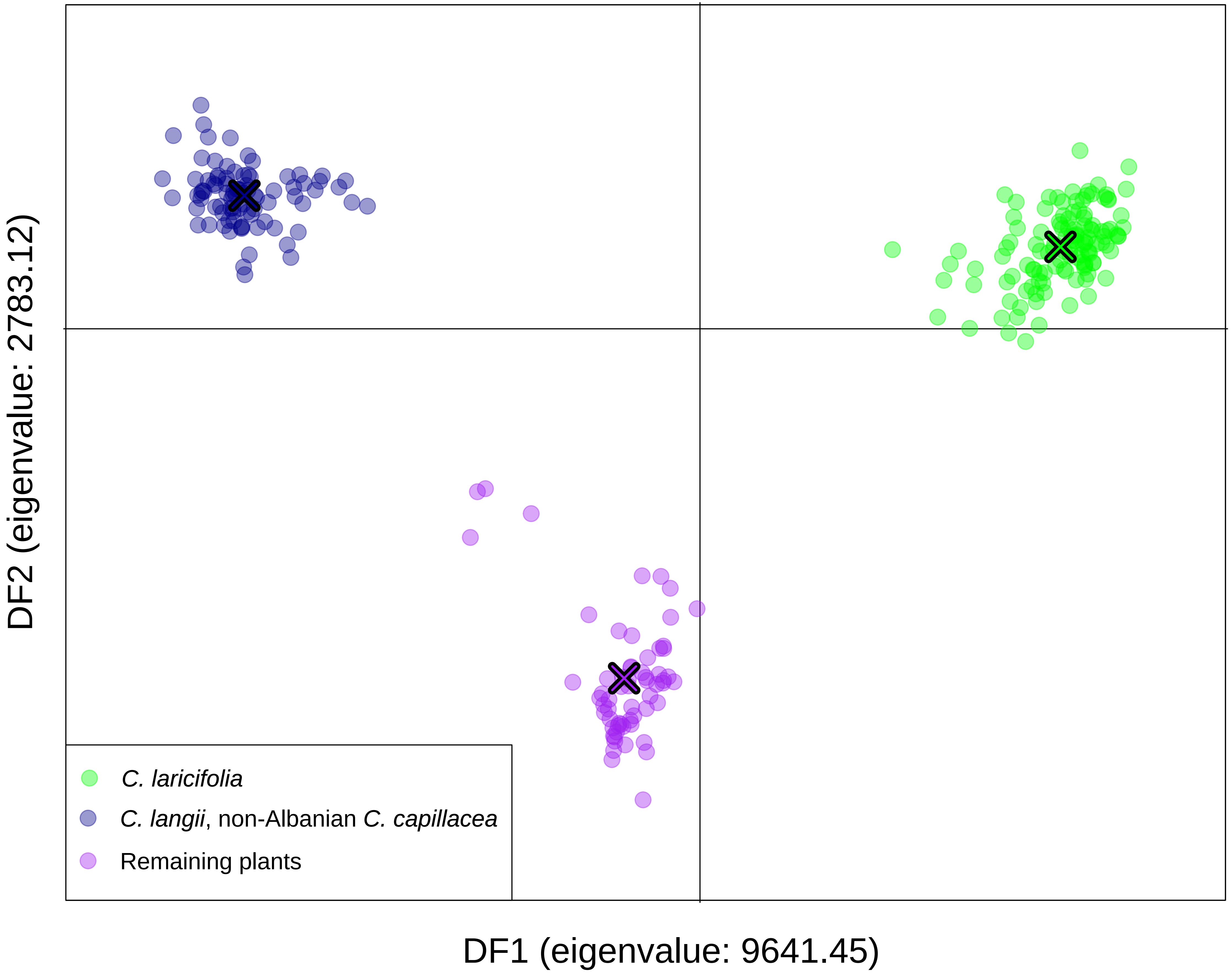
DF2 (eigenvalue: 2783.12)
DF1 (eigenvalue: 9641.45)
C. laricifolia
C. langii, non-Albanian *C. capillacea*
Remaining plants

Fig. S8c

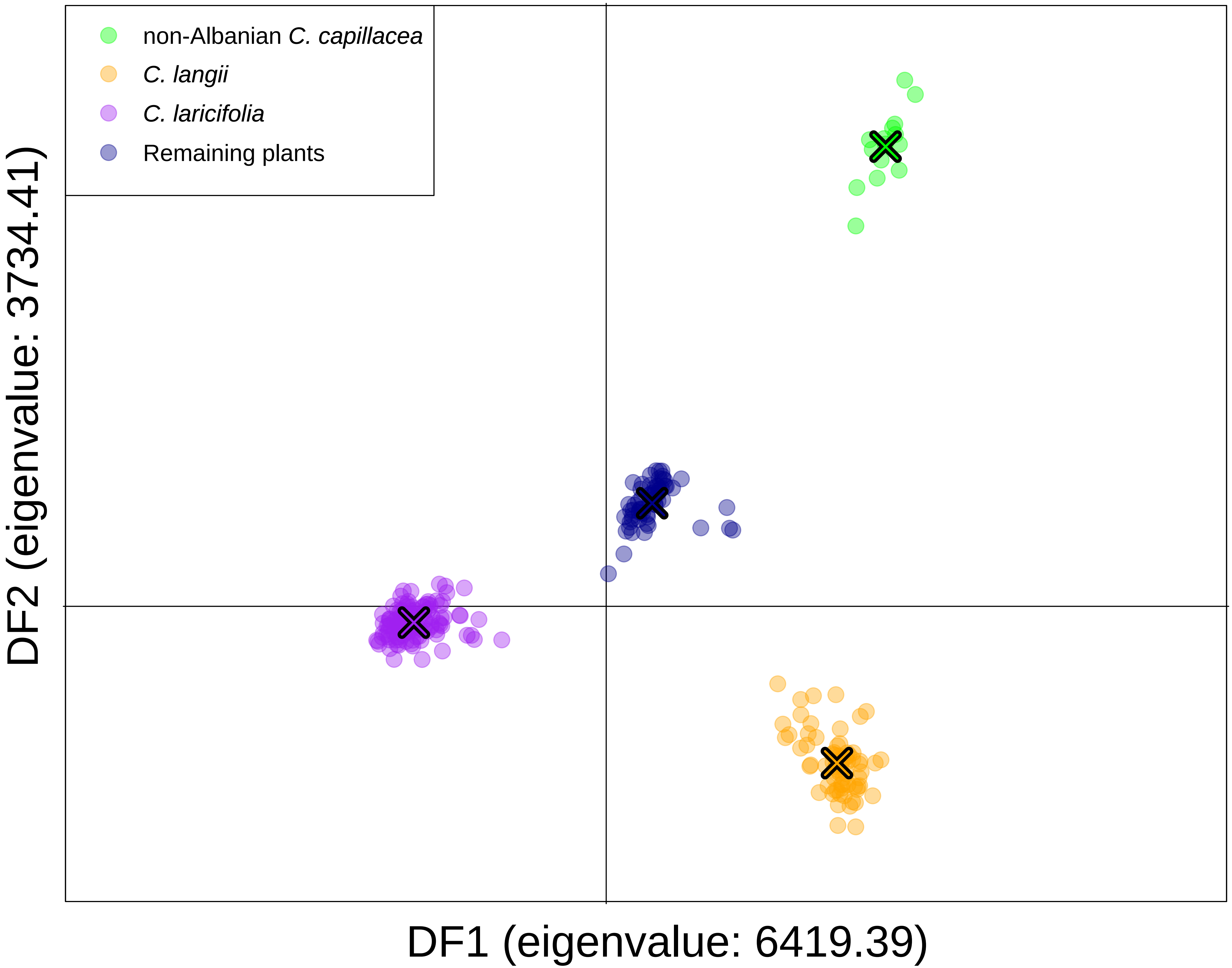
non-Albanian C. capillacea
C. langii
C. laricifolia
Remaining plants
DF1 (eigenvalue: 6419.39)
DF2 (eigenvalue: 3734.41)

Fig. S8d

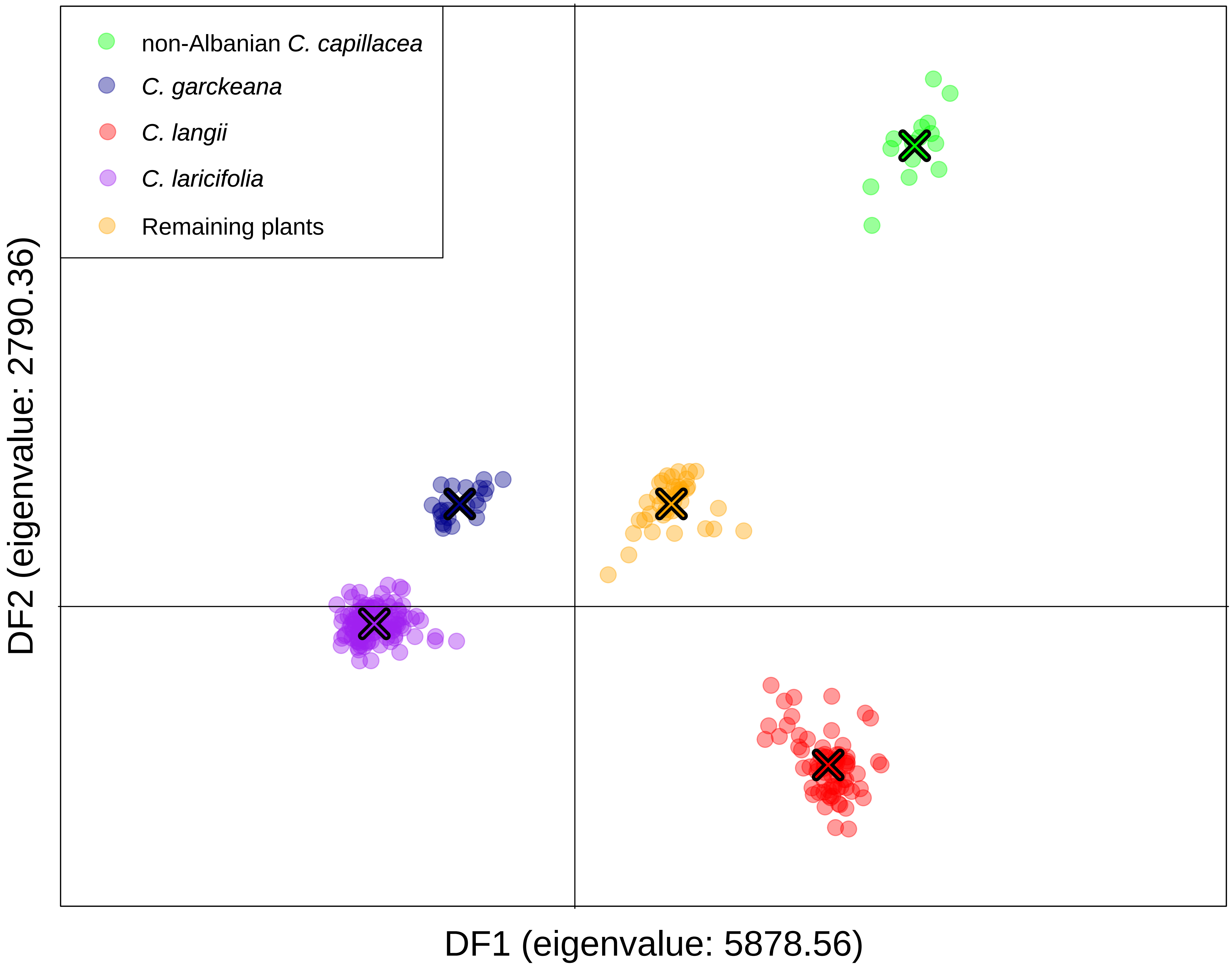

non-Albanian *C. capillacea*
C. garckeana
C. langii
C. laricifolia
Remaining plants
DF2 (eigenvalue: 2790.36)
DF1 (eigenvalue: 5878.56)

Fig. S8e

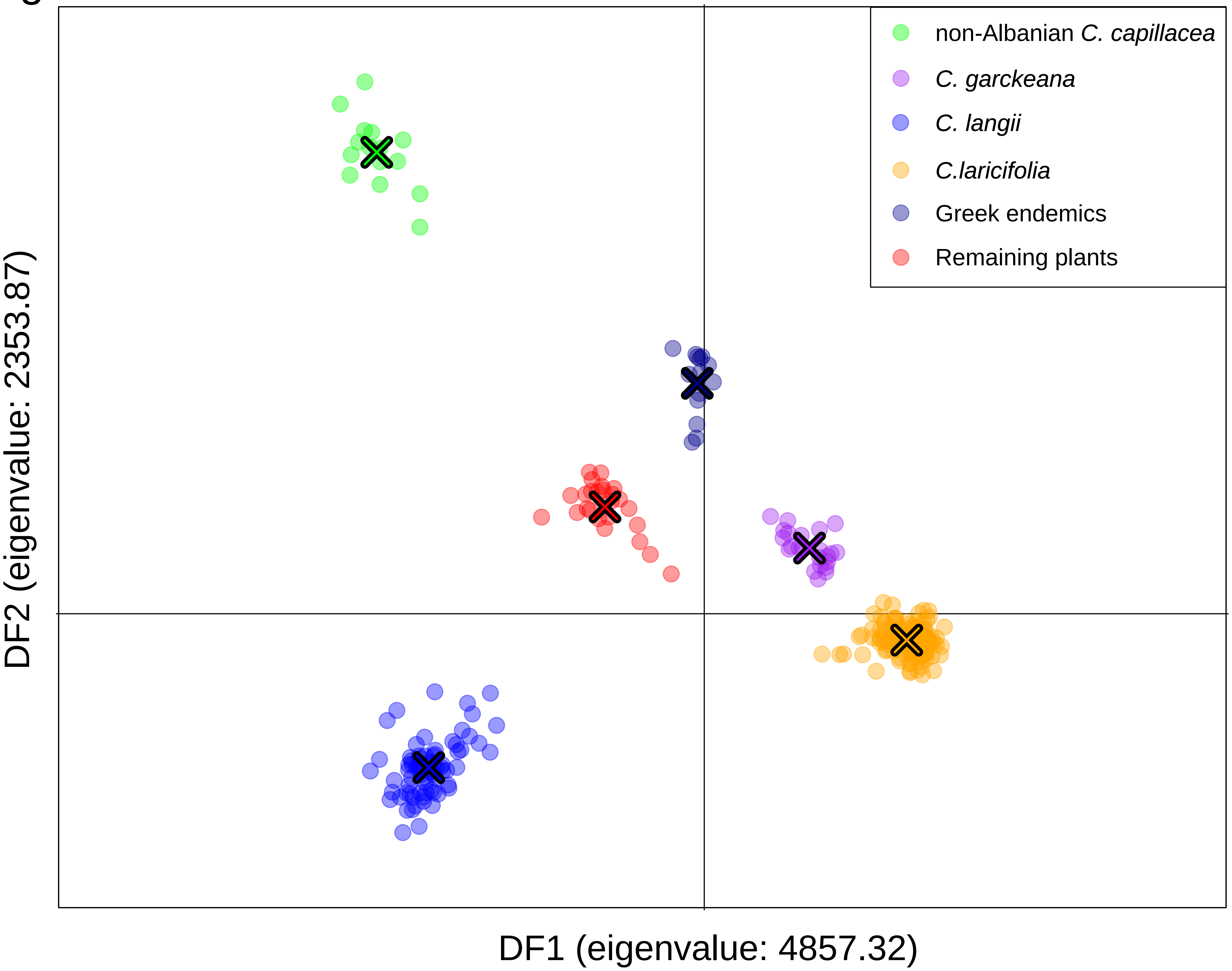
non-Albanian *C. capillacea*
C. garckeana
C. langii
C.laricifolia
Greek endemics
Remaining plants
DF2 (eigenvalue: 2353.87)
DF1 (eigenvalue: 4857.32)

Fig. S8f

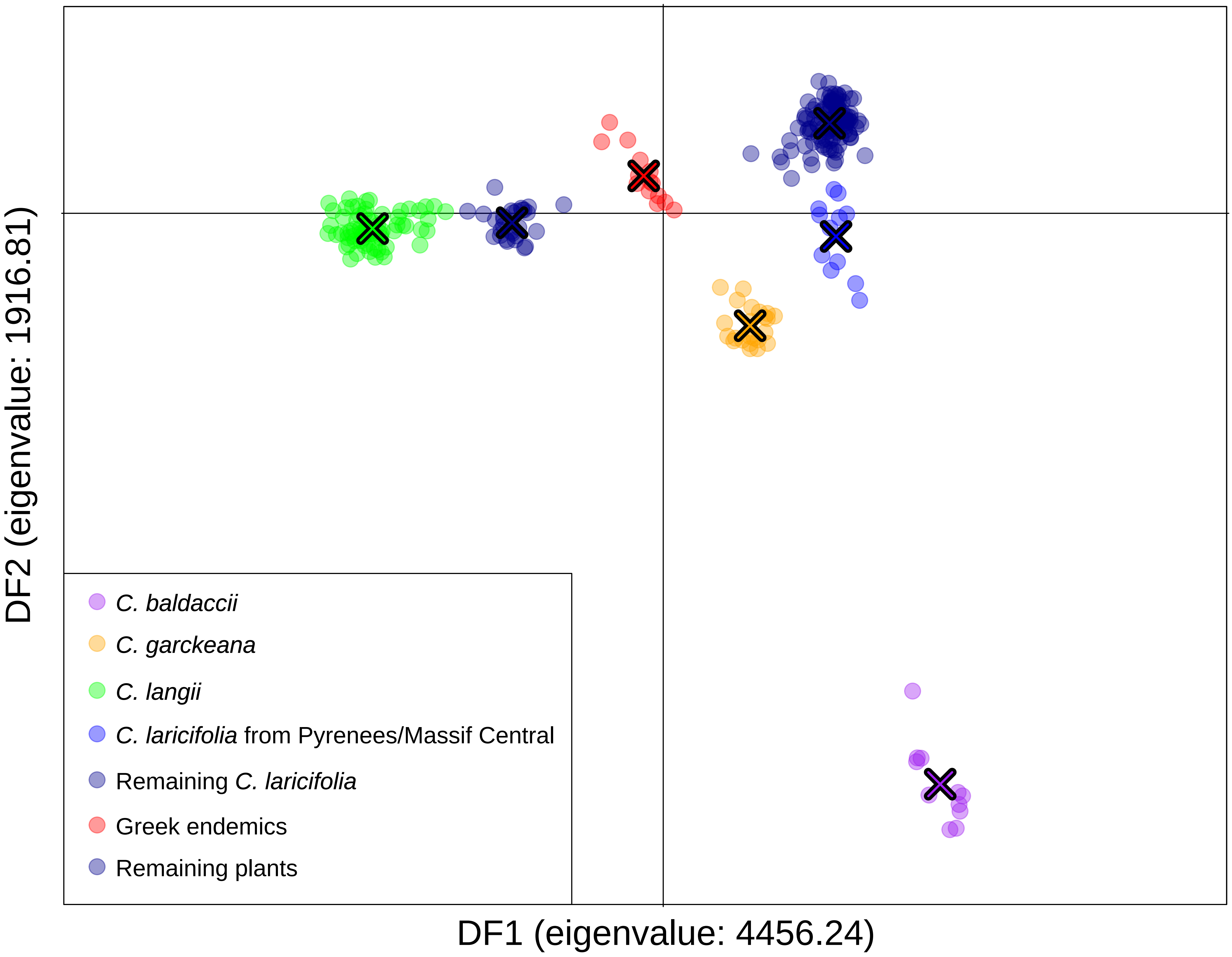

C. baldaccii
C. garckeana
C. langii
C. laricifolia from Pyrenees/Massif Central
Remaining C. laricifolia
Greek endemics
Remaining plants
DF1 (eigenvalue: 4456.24)
DF2 (eigenvalue: 1916.81)

Fig.S9

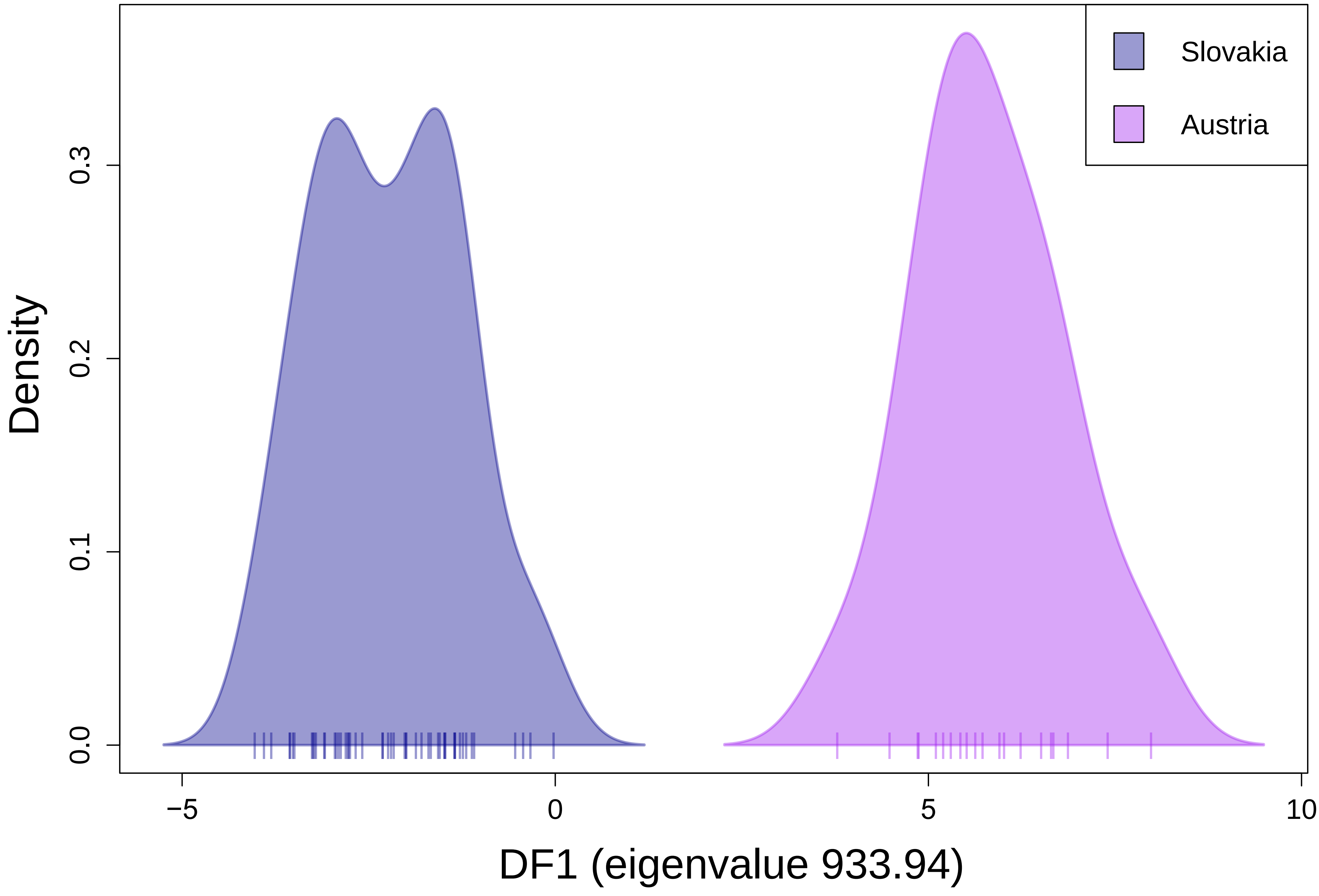

Slovakia
Austria
Density
0.0
0.1
0.2
0.3
−5
0
5
10
DF1 (eigenvalue 933.94)

Fig. S10a

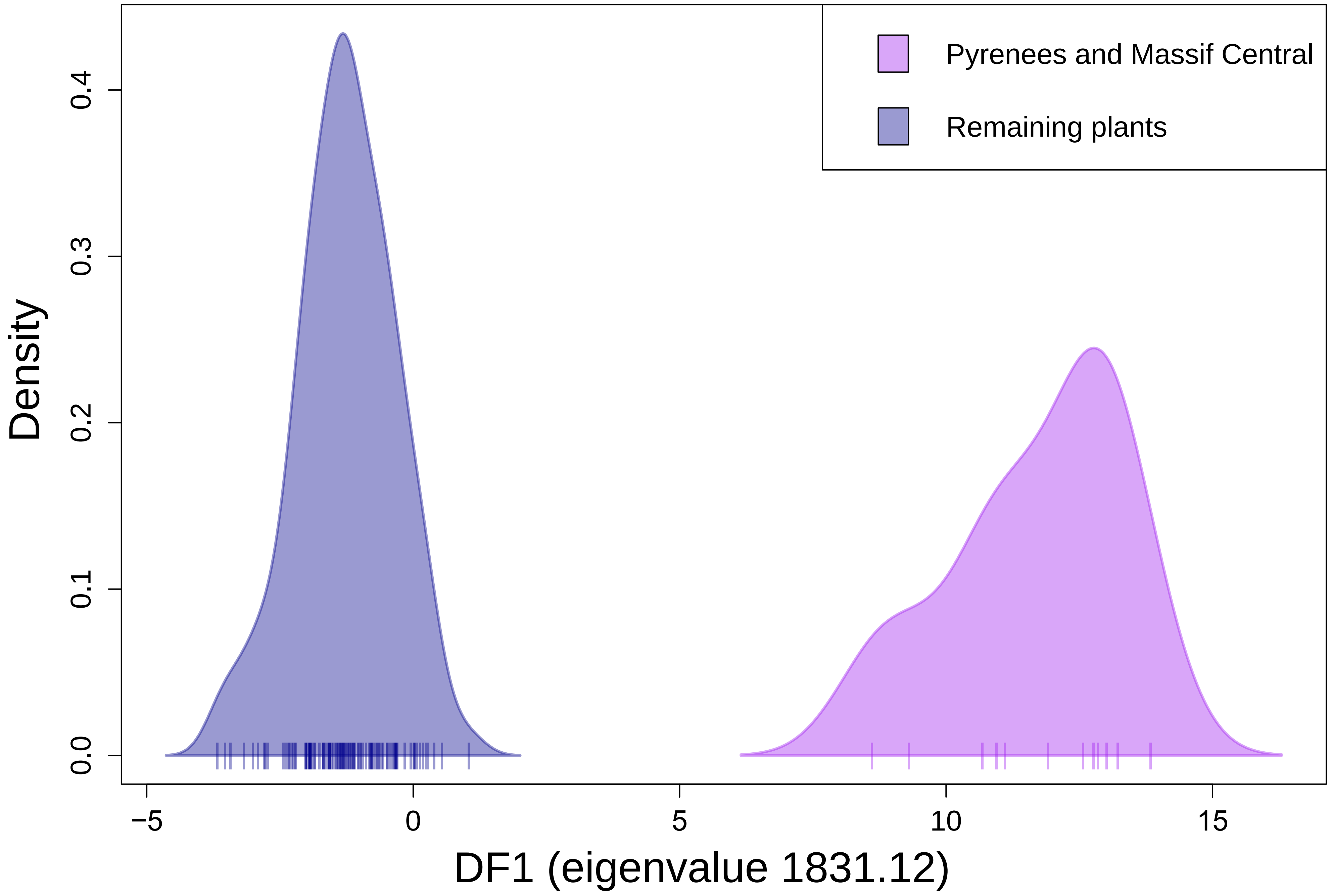
Pyrenees and Massif Central
Remaining plants
Density
0.0
0.1
0.2
0.3
0.4
−5
0
5
10
15
DF1 (eigenvalue 1831.12)

Fig. S10b

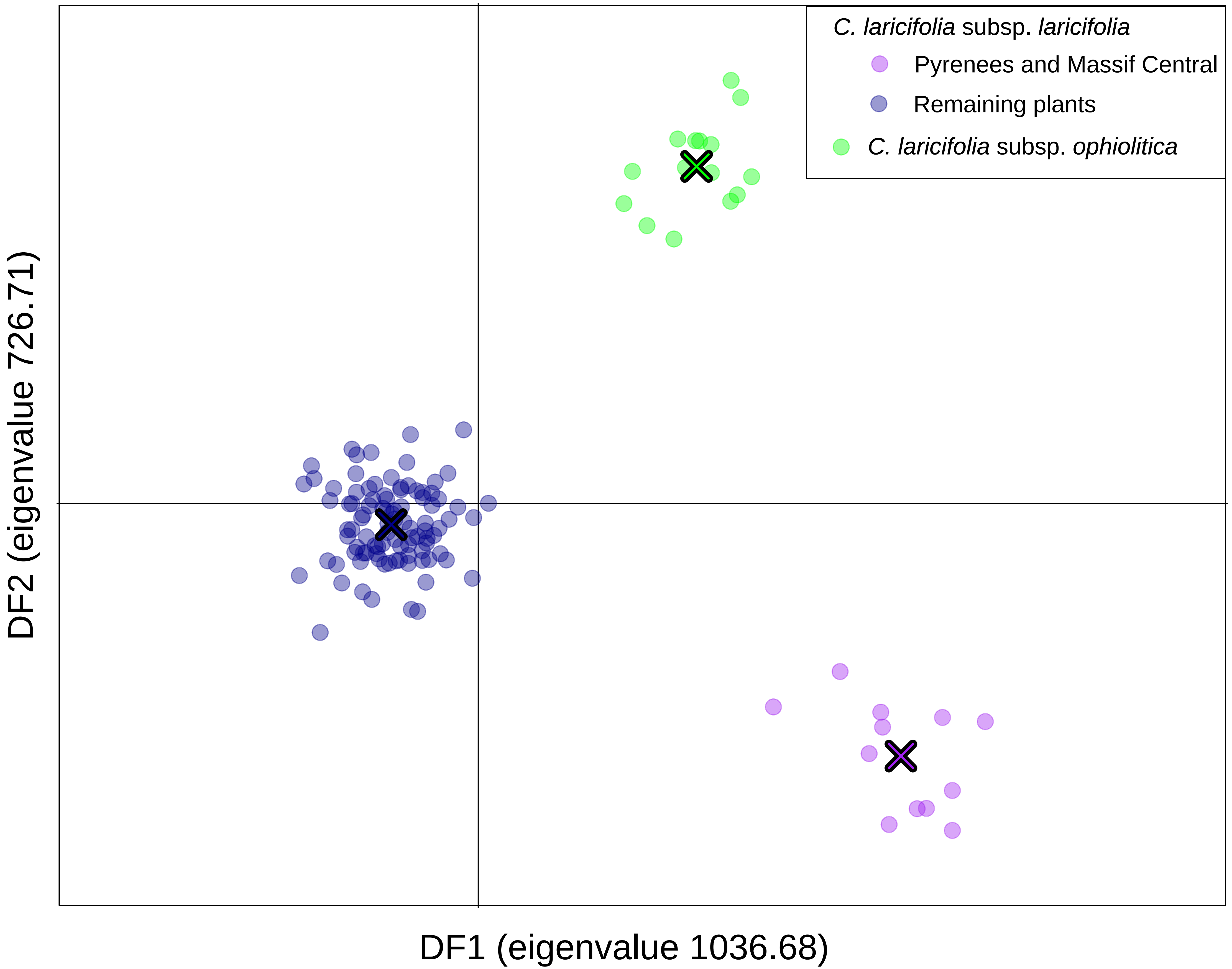
C. laricifolia subsp. laricifolia
Pyrenees and Massif Central
Remaining plants
C. laricifolia subsp. ophiolitica
DF2 (eigenvalue 726.71)
DF1 (eigenvalue 1036.68)

Fig. S11

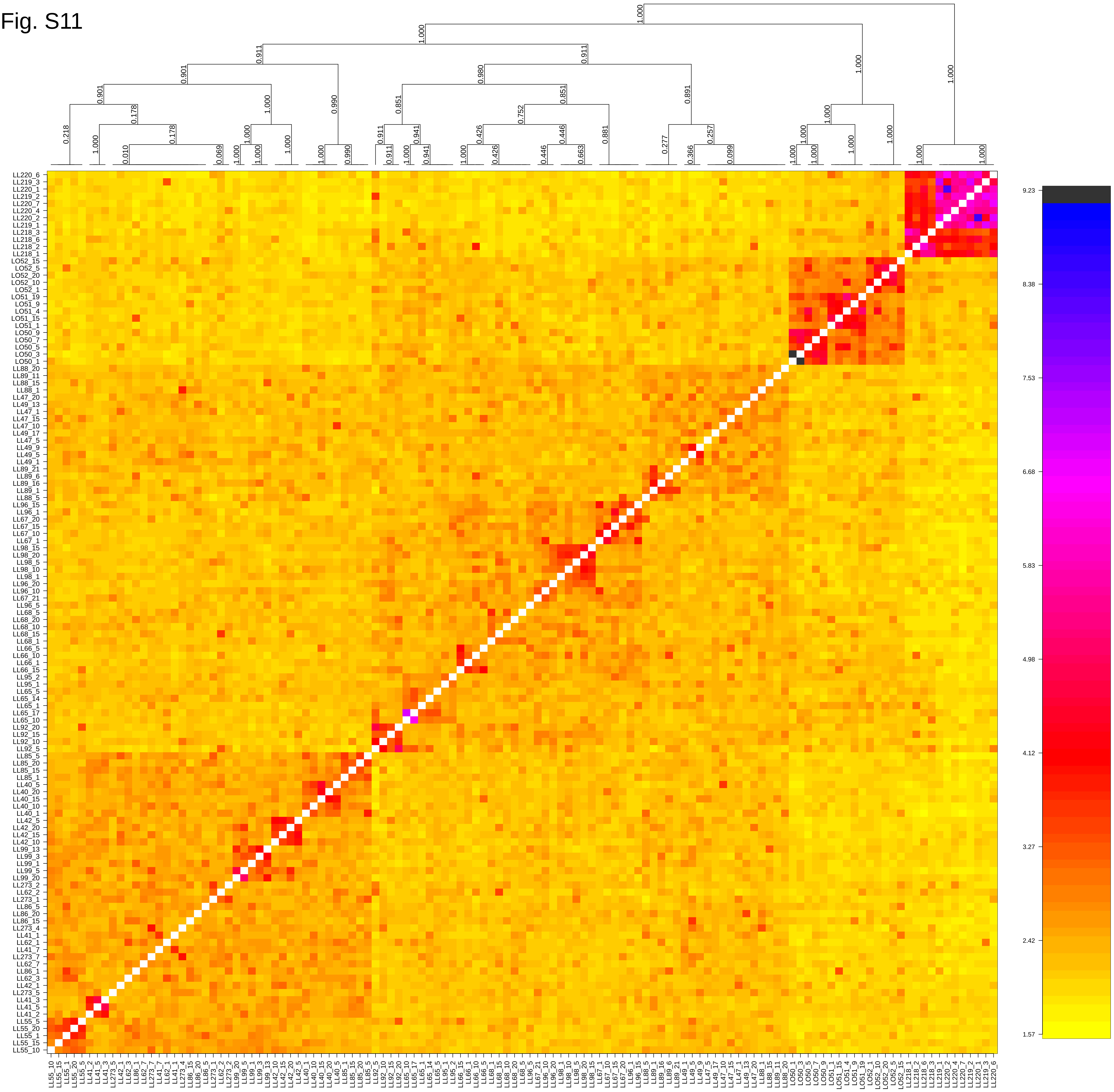
LL220_6
LL219_3
LL220_1
LL219_2
LL220_7
LL220_4
LL220_2
LL219_1
LL218_3
LL218_6
LL218_2
LL218_1
LO52_15
LO52_5
LO52_20
LO52_10
LO52_1
LO51_19
LO51_9
LO51_4
LO51_15
LO51_1
LO50_9
LO50_7
LO50_5
LO50_3
LO50_1
LL88_20
LL89_11
LL88_15
LL88_1
LL47_20
LL49_13
LL47_1
LL47_15
LL47_10
LL49_17
LL47_5
LL49_9
LL49_5
LL49_1
LL89_21
LL89_6
LL89_16
LL89_1
LL88_5
LL96_15
LL96_1
LL67_20
LL67_15
LL67_10
LL67_1
LL98_15
LL98_20
LL98_5
LL98_10
LL98_1
LL96_20
LL96_10
LL67_21
LL96_5
LL68_5
LL68_20
LL68_10
LL68_15
LL68_1
LL66_5
LL66_10
LL66_1
LL66_15
LL95_2
LL95_1
LL65_5
LL65_14
LL65_1
LL65_17
LL65_10
LL92_20
LL92_15
LL92_10
LL92_5
LL85_5
LL85_20
LL85_15
LL85_1
LL40_5
LL40_20
LL40_15
LL40_10
LL40_1
LL42_5
LL42_20
LL42_15
LL42_10
LL99_13
LL99_3
LL99_1
LL99_5
LL99_20
LL273_2
LL62_2
LL273_1
LL86_5
LL86_20
LL86_15
LL273_4
LL41_1
LL62_1
LL41_7
LL273_7
LL62_7
LL86_1
LL62_3
LL42_1
LL273_5
LL41_3
LL41_5
LL41_2
LL55_5
LL55_20
LL55_1
LL55_15
LL55_10
9.23
8.38
7.53
6.68
5.83
4.98
4.12
3.27
2.42
1.57

Fig. S12

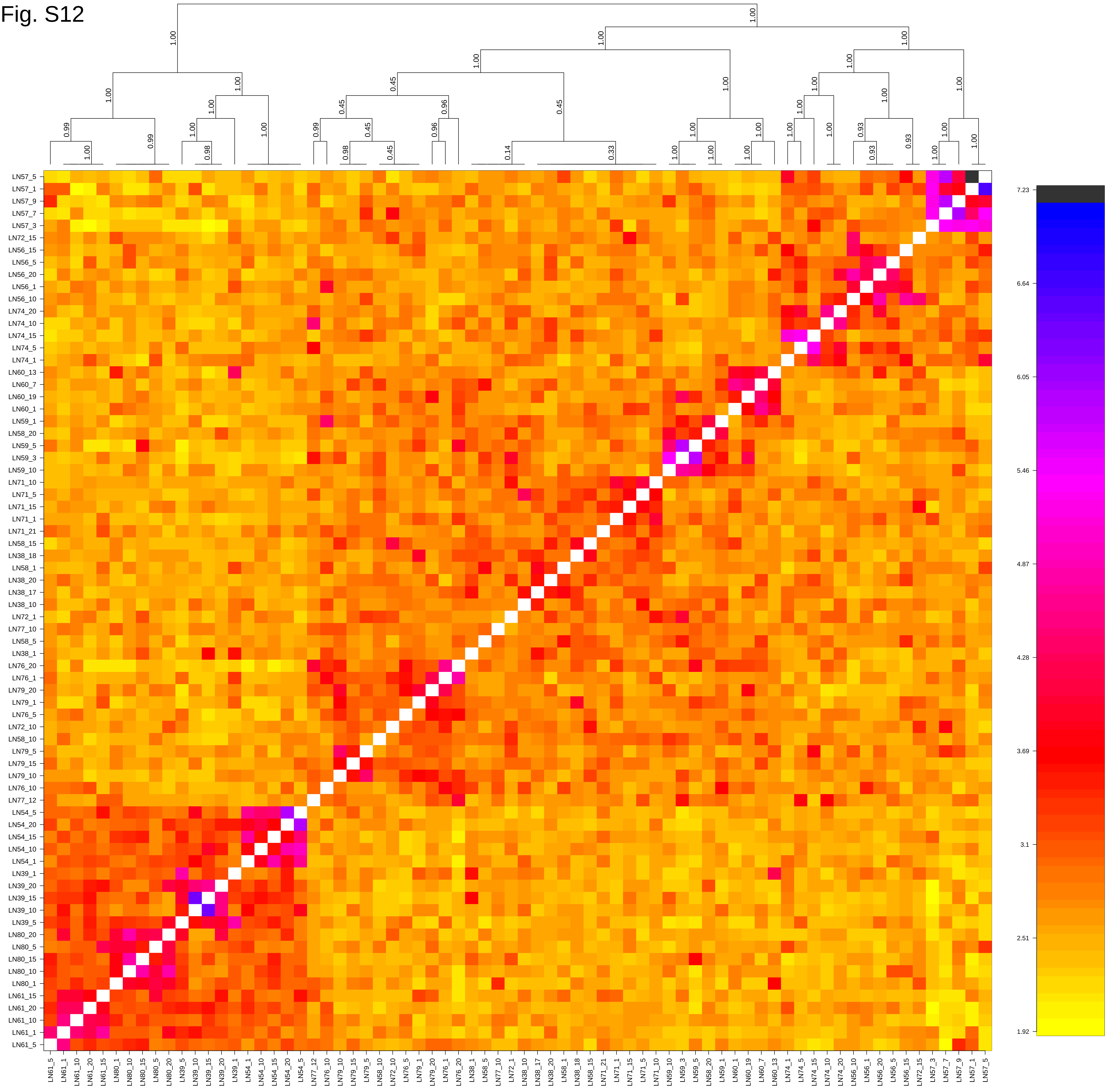

0.99 1.00 1.00 0.99 1.00 1.00 0.98 1.00 1.00 1.00 0.99 0.45 0.98 0.45 0.45 0.45 0.96 0.96 1.00 0.14 0.45 1.00 0.33 1.00 1.00 1.00 1.00 1.00 1.00 1.00 1.00 1.00 1.00 1.00 1.00 0.93 0.93 1.00 1.00 0.93 1.00 1.00 1.00 1.00
LN57_5
LN57_1
LN57_9
LN57_7
LN57_3
LN72_15
LN56_15
LN56_5
LN56_20
LN56_1
LN56_10
LN74_20
LN74_10
LN74_15
LN74_5
LN74_1
LN60_13
LN60_7
LN60_19
LN60_1
LN59_1
LN58_20
LN59_5
LN59_3
LN59_10
LN71_10
LN71_5
LN71_15
LN71_1
LN71_21
LN58_15
LN38_18
LN58_1
LN38_20
LN38_17
LN38_10
LN72_1
LN77_10
LN58_5
LN38_1
LN76_20
LN76_1
LN79_20
LN79_1
LN76_5
LN72_10
LN58_10
LN79_5
LN79_15
LN79_10
LN76_10
LN77_12
LN54_5
LN54_20
LN54_15
LN54_10
LN54_1
LN39_1
LN39_20
LN39_15
LN39_10
LN39_5
LN80_20
LN80_5
LN80_15
LN80_10
LN80_1
LN61_15
LN61_20
LN61_10
LN61_1
LN61_5
7.23
6.64
6.05
5.46
4.87
4.28
3.69
3.1
2.51
1.92
