# Supplementary material for: Range and niche expansion through multiple interspecific hybridization: a genotyping by sequencing analysis of Cherleria (Caryophyllaceae)
Source: BMC Ecol Evol. 2021 Mar 10;21:40. doi: 10.1186/s12862-020-01721-5 (PMC7945309; doi:10.1186/s12862-020-01721-5)

Fig. S13a

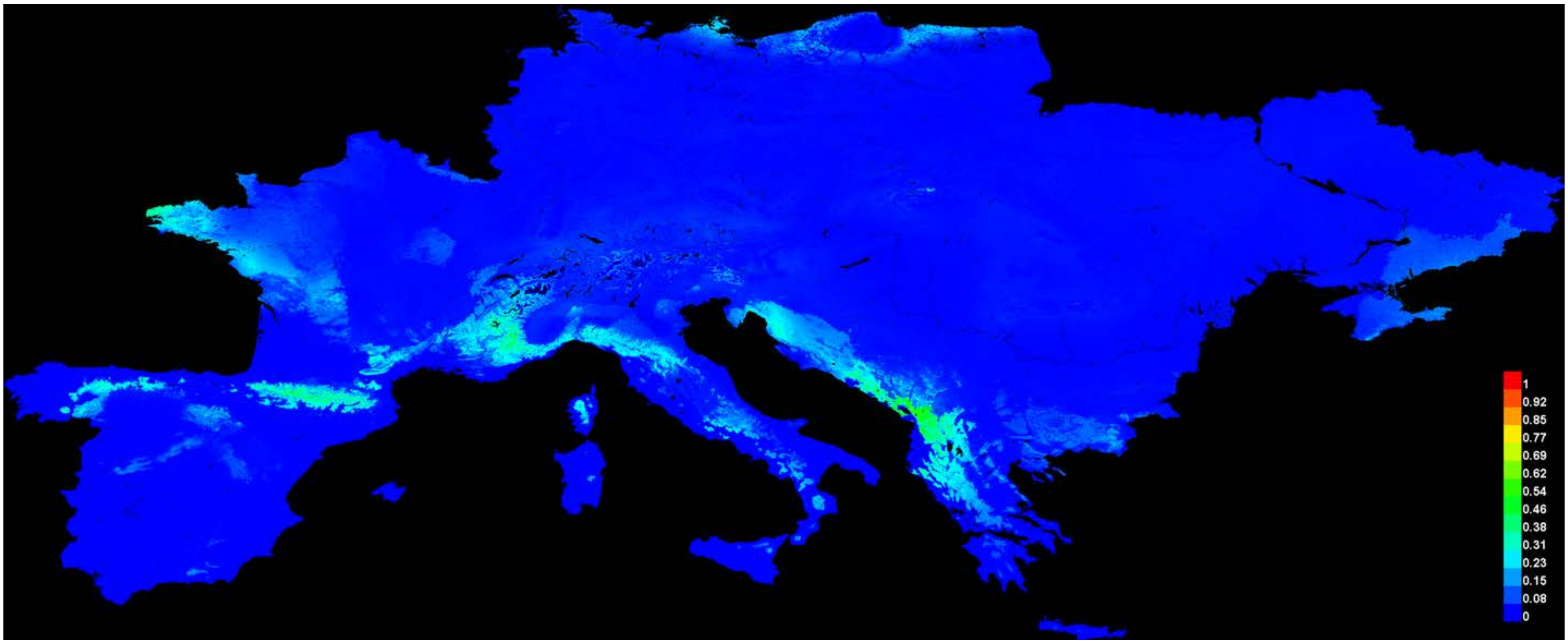

Fig. S13b

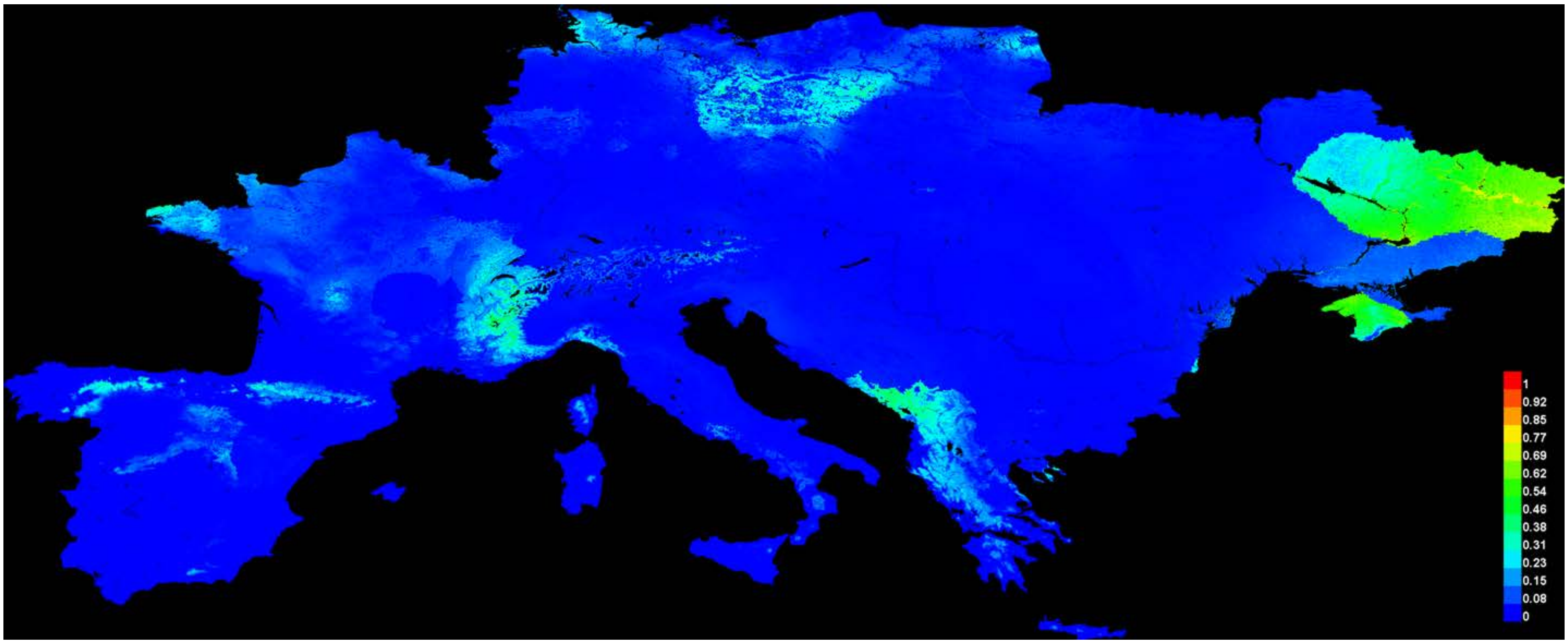

Fig. S13c

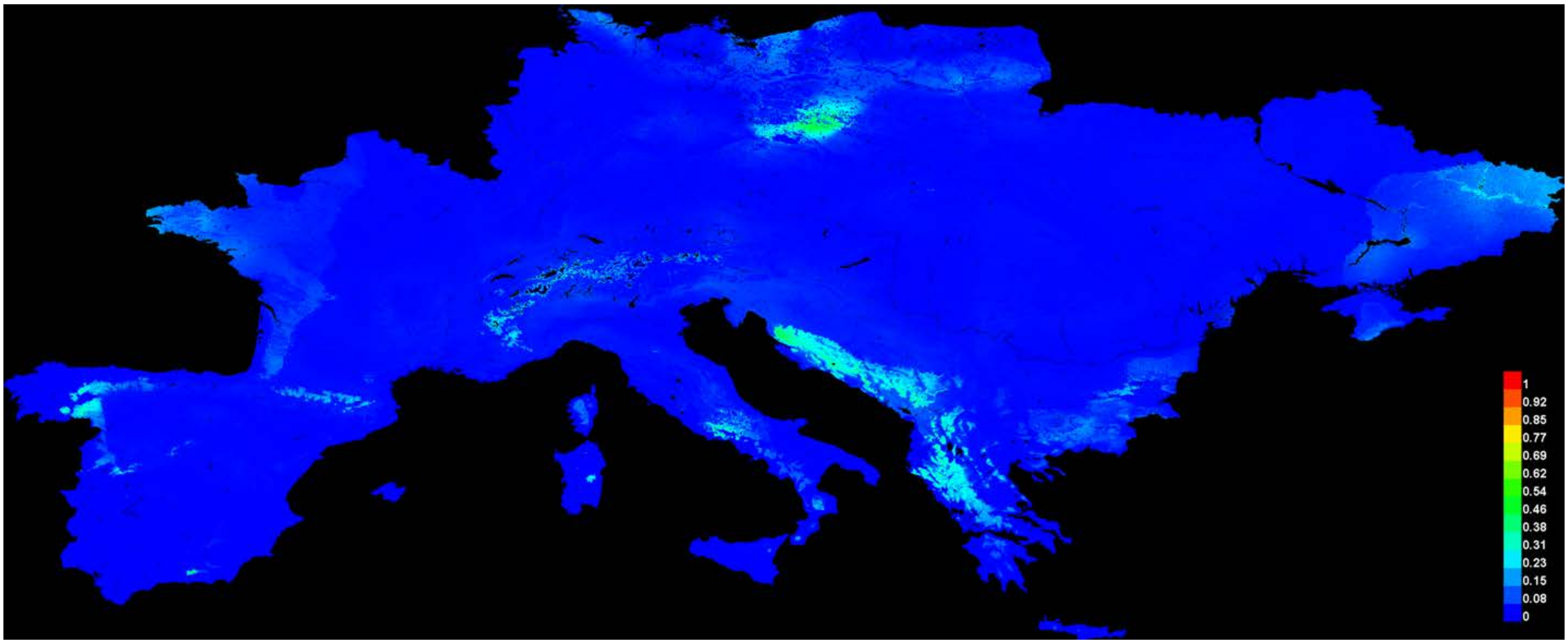

Fig. S13d

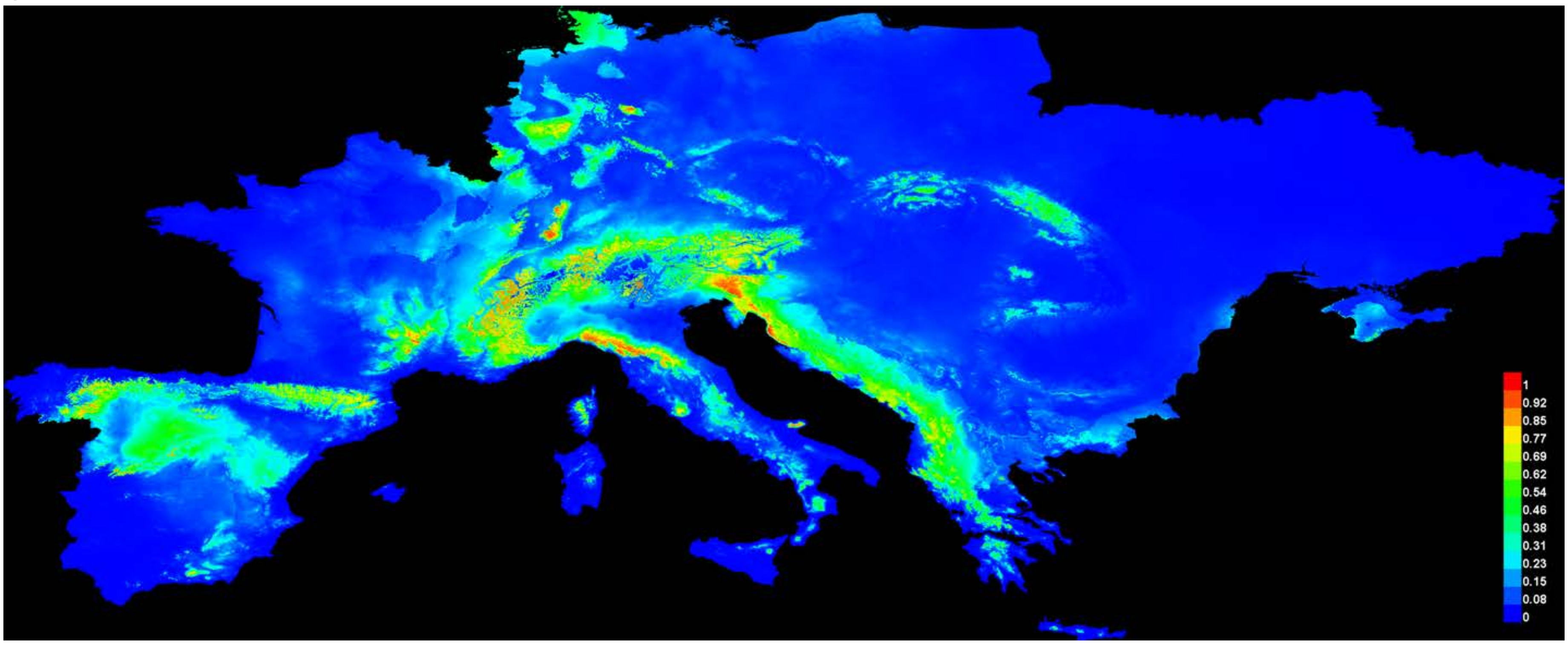

Fig. S13e

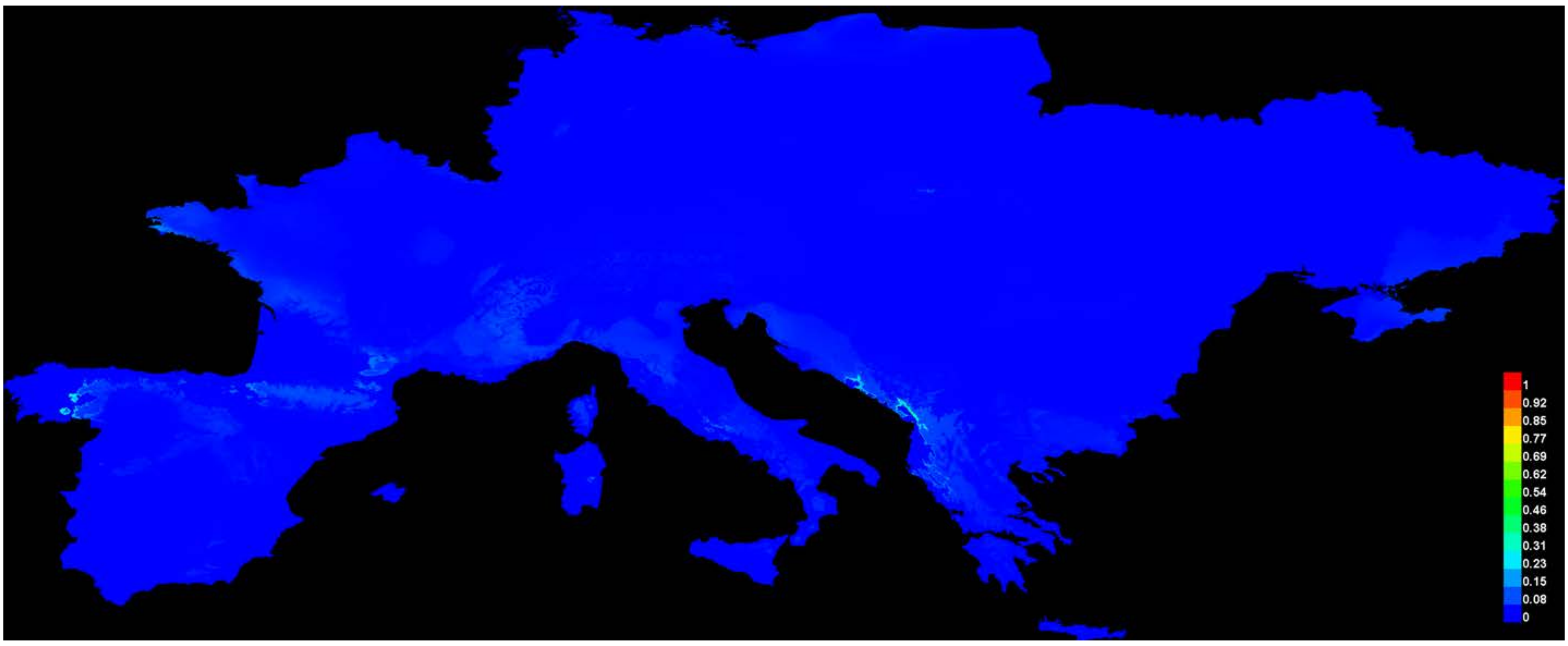

Fig. S13f

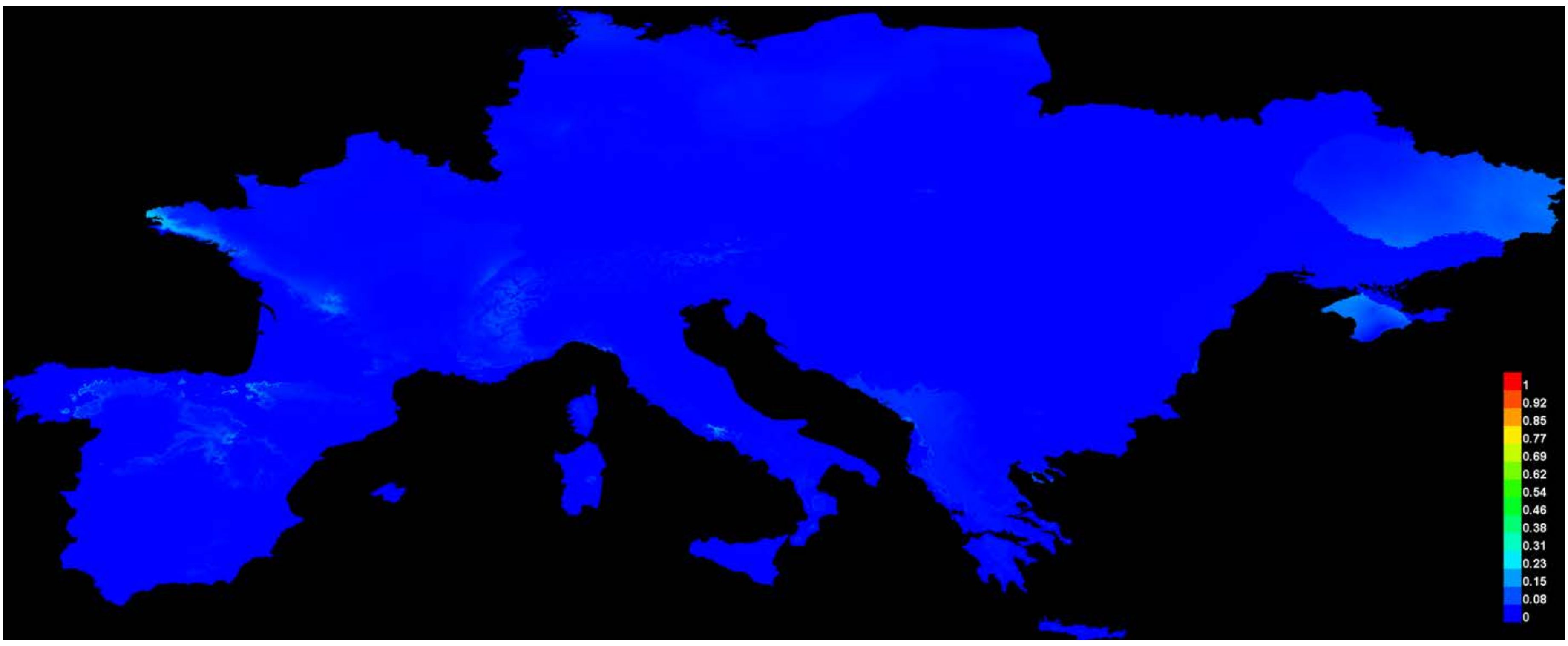

Fig. S13g

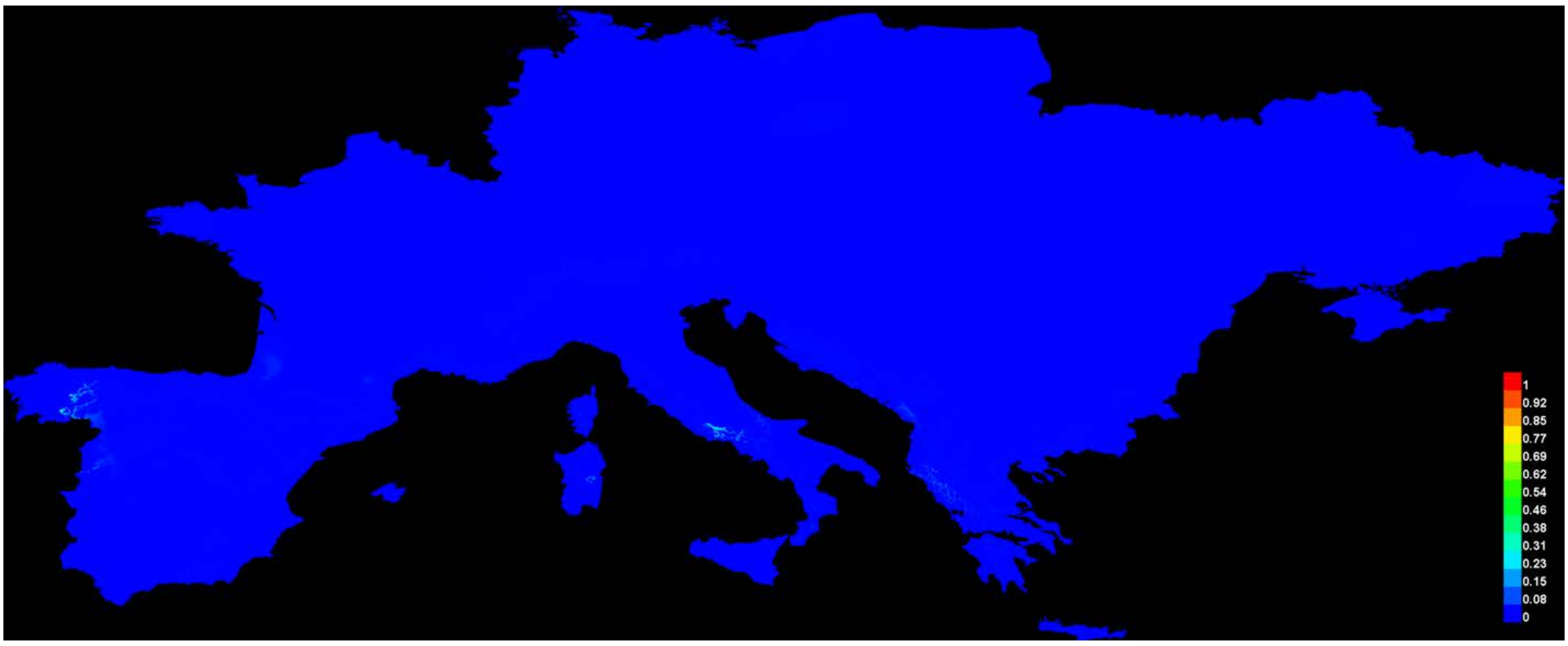

Fig. S14a

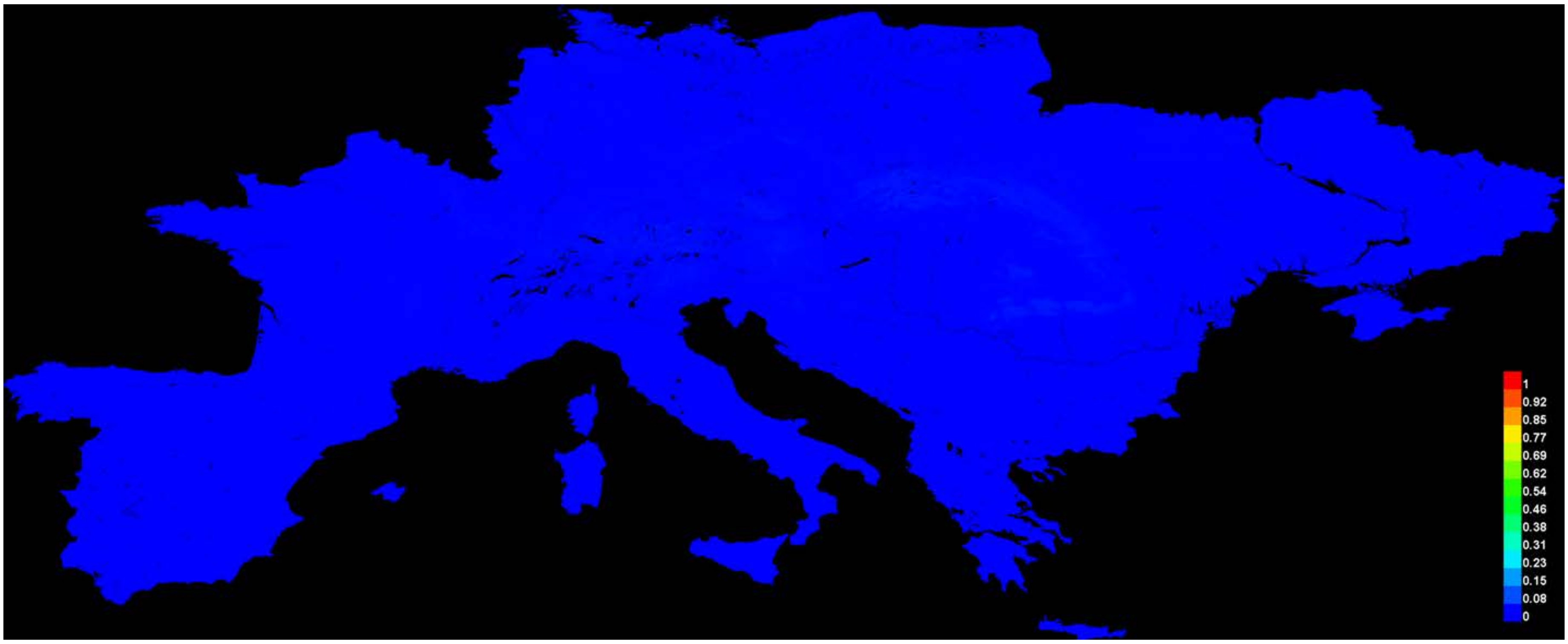

Fig. S14b

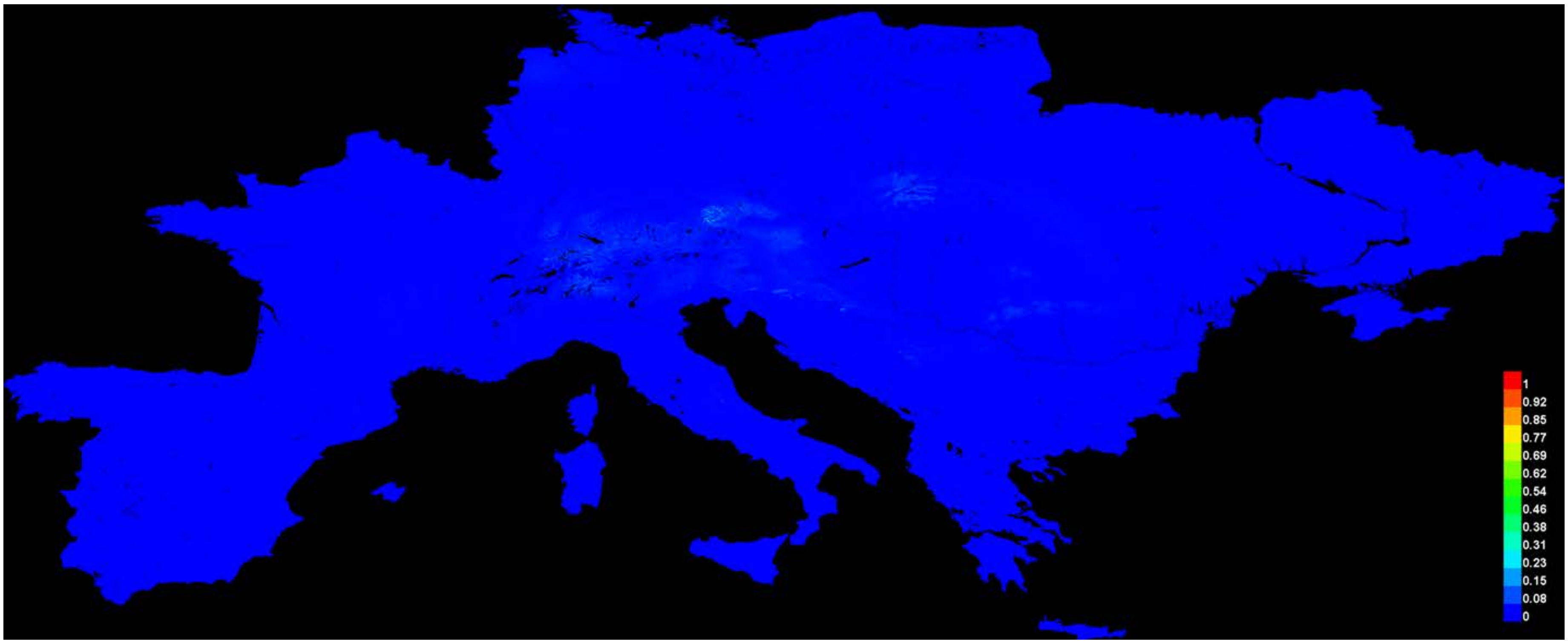

Fig. S14c

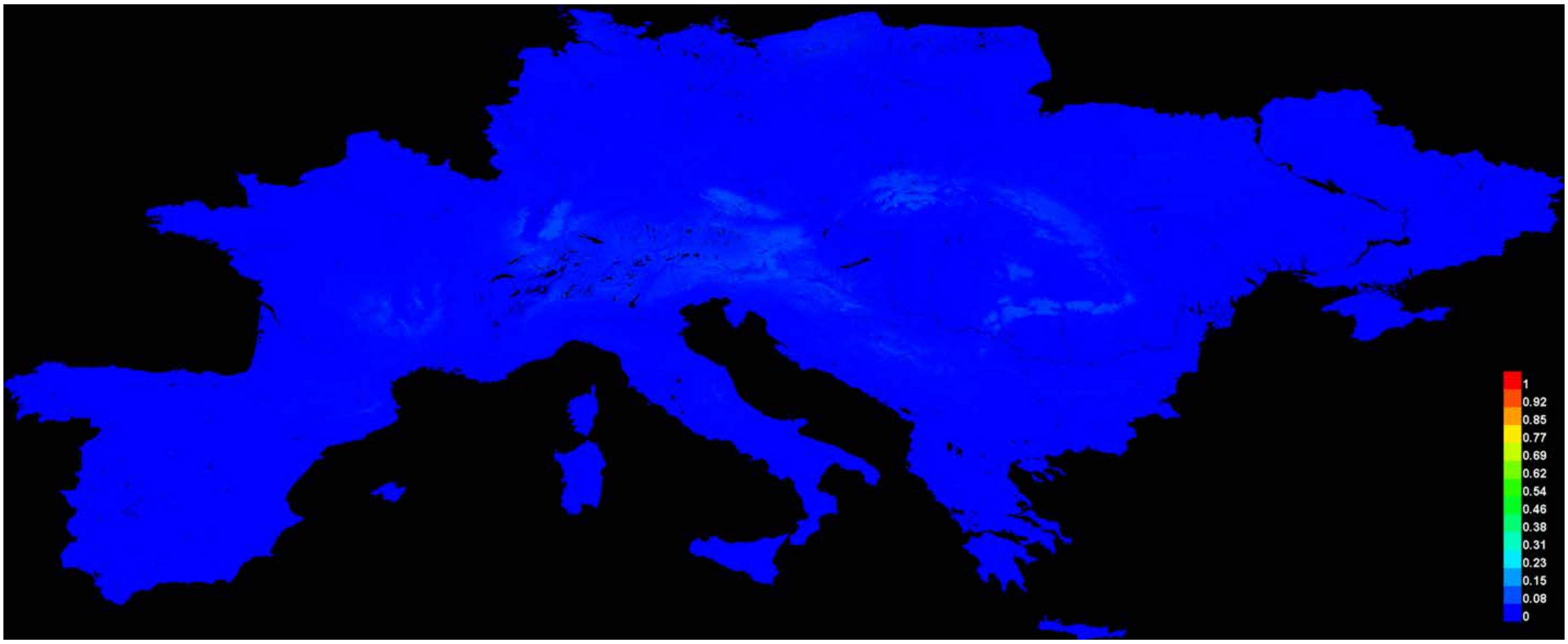

Fig. S14d

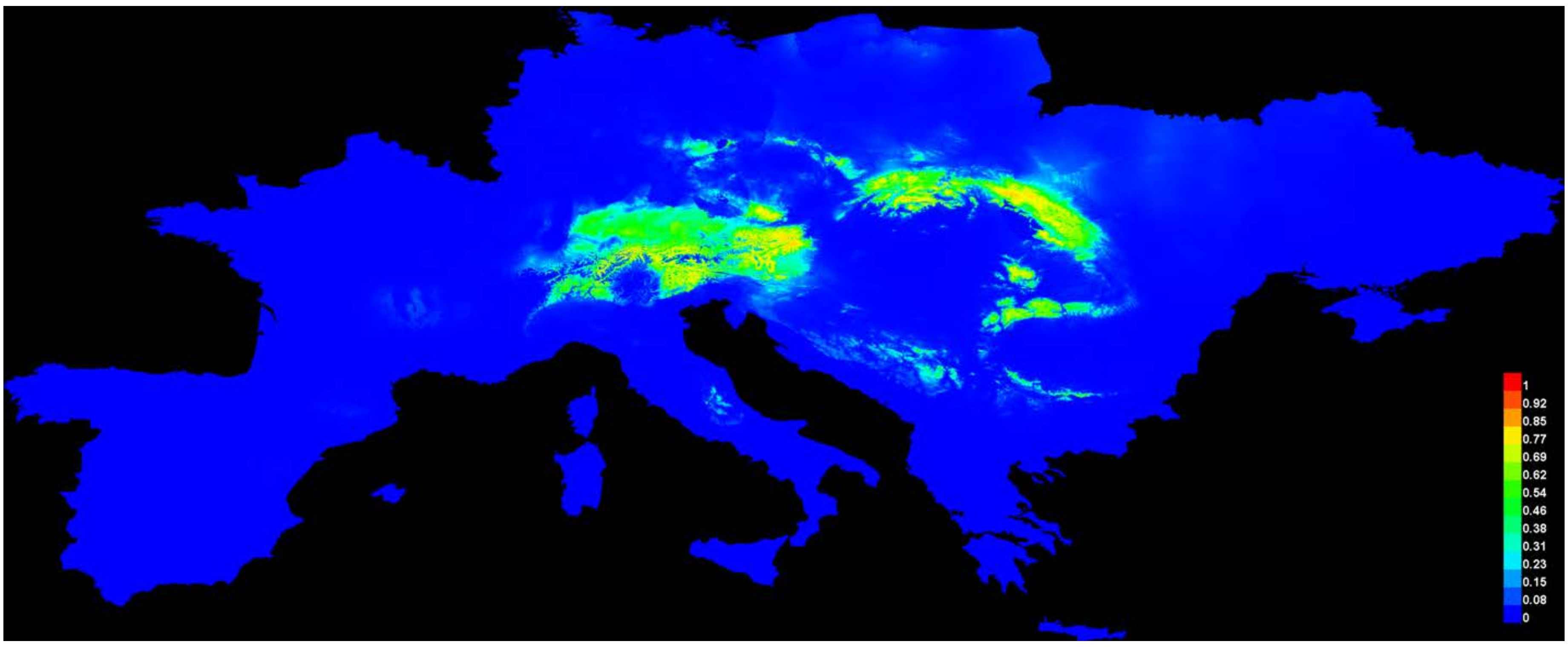

Fig. S14e

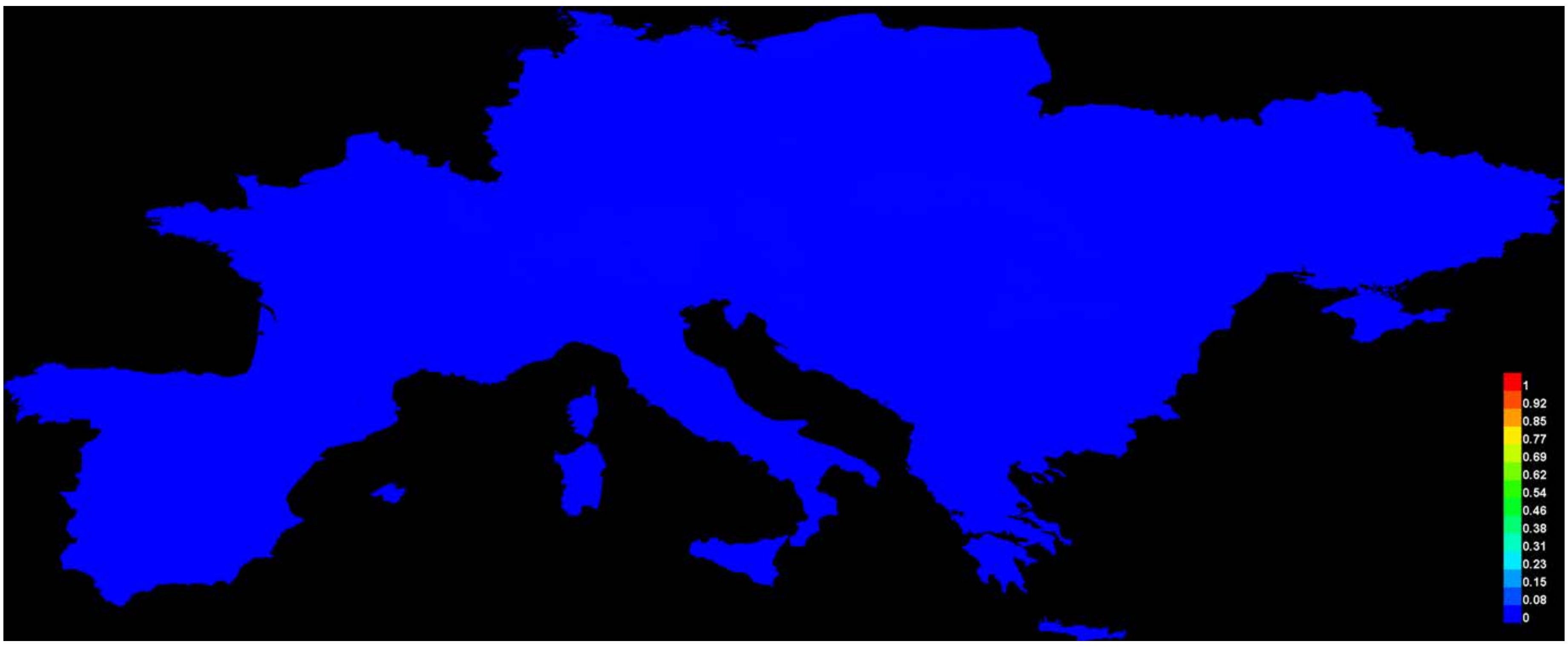

Fig. S14f

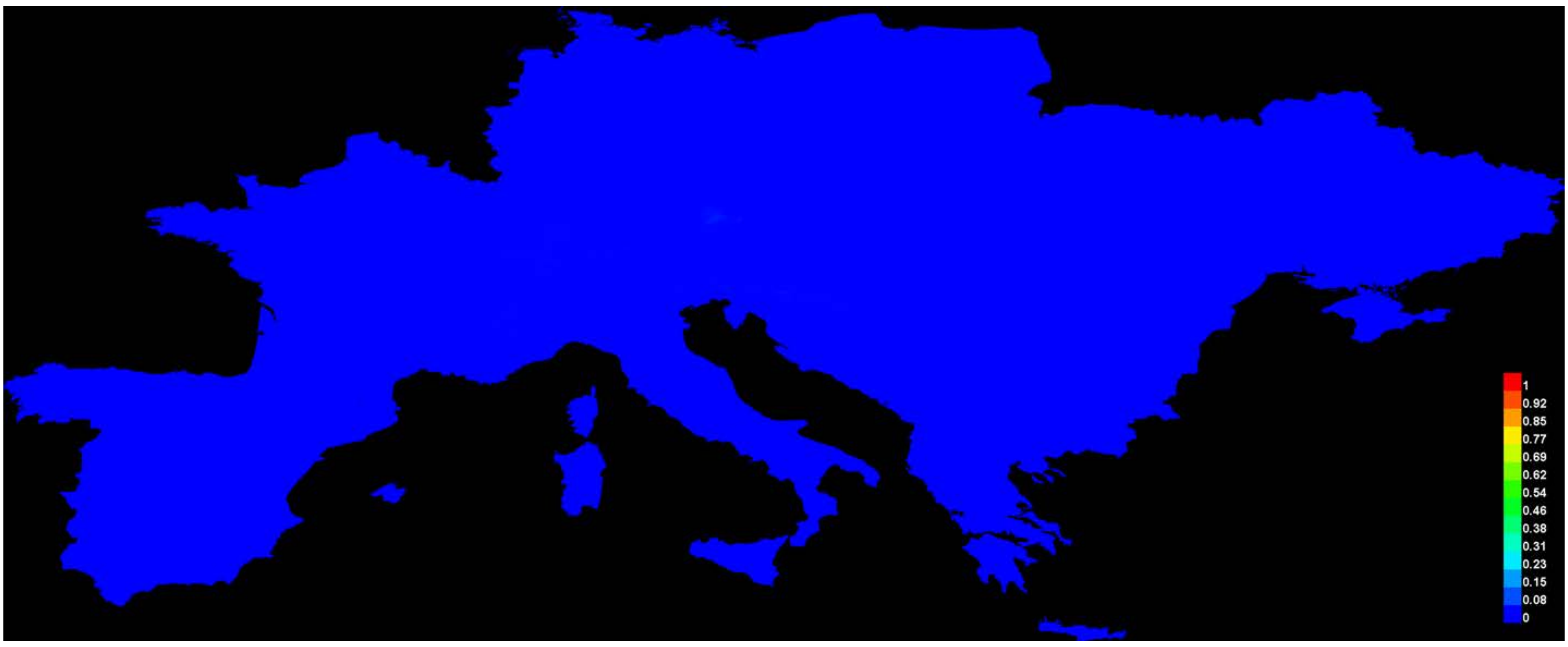

Fig. S14g

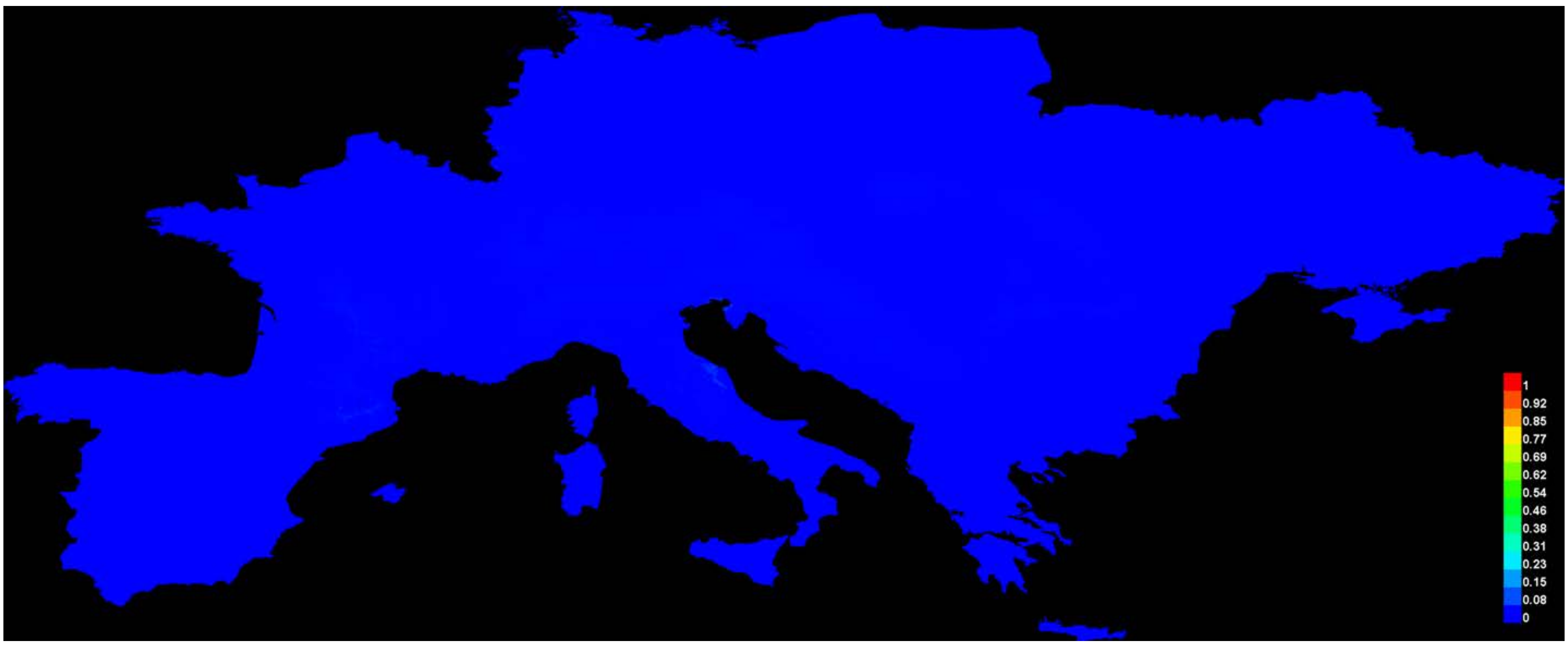

Fig. S15a

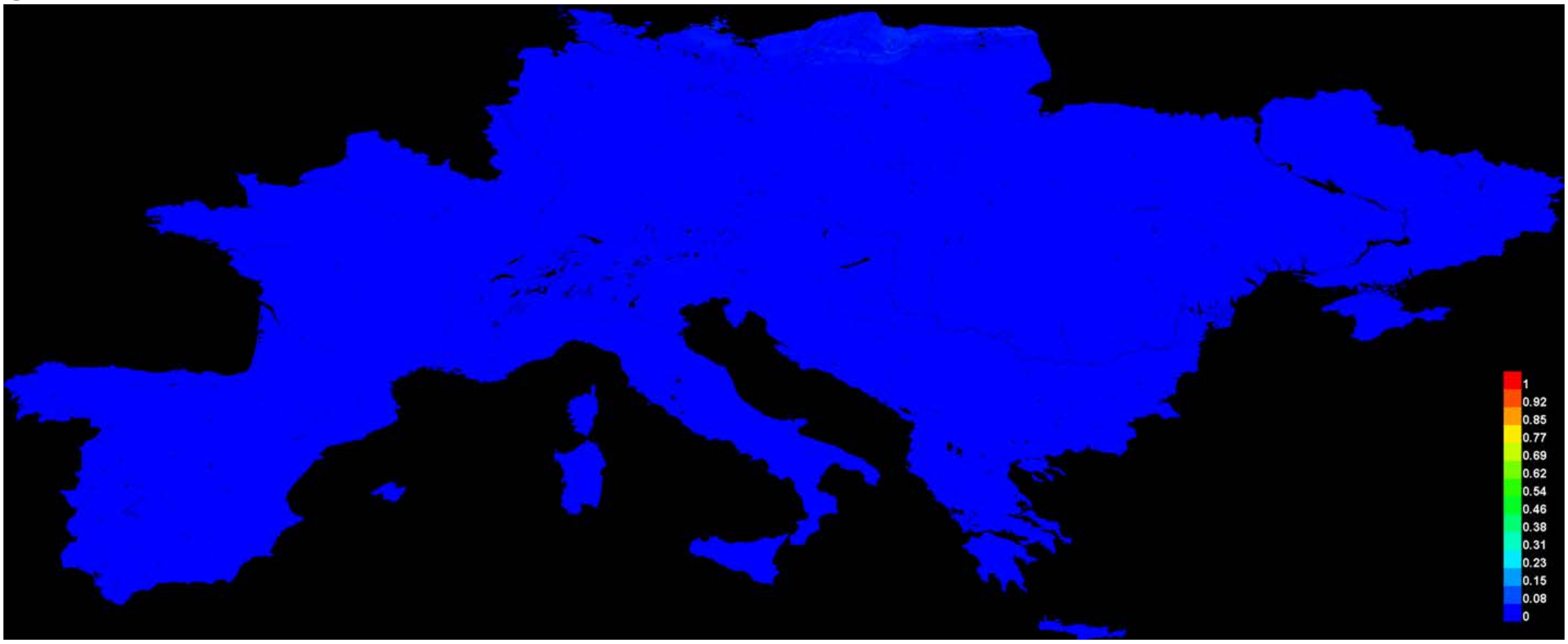

Fig. S15b

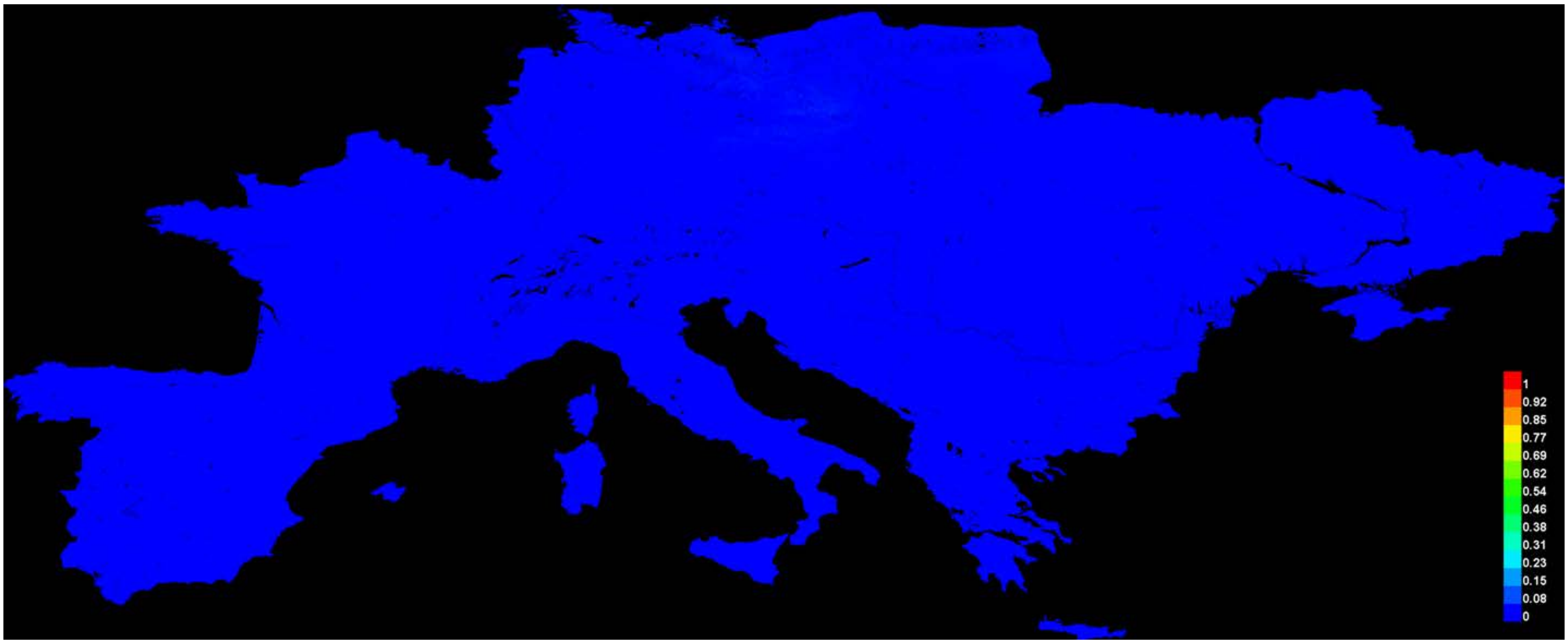

Fig. S15c

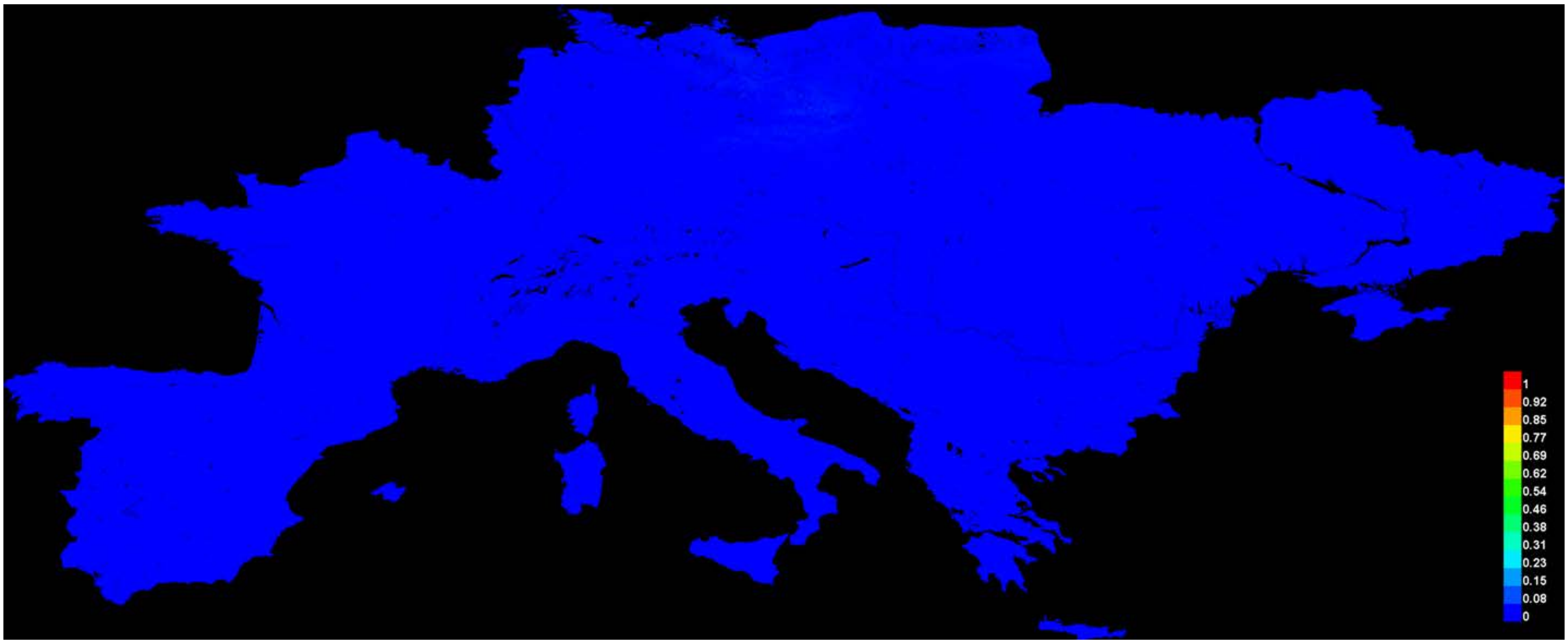

Fig. S15d

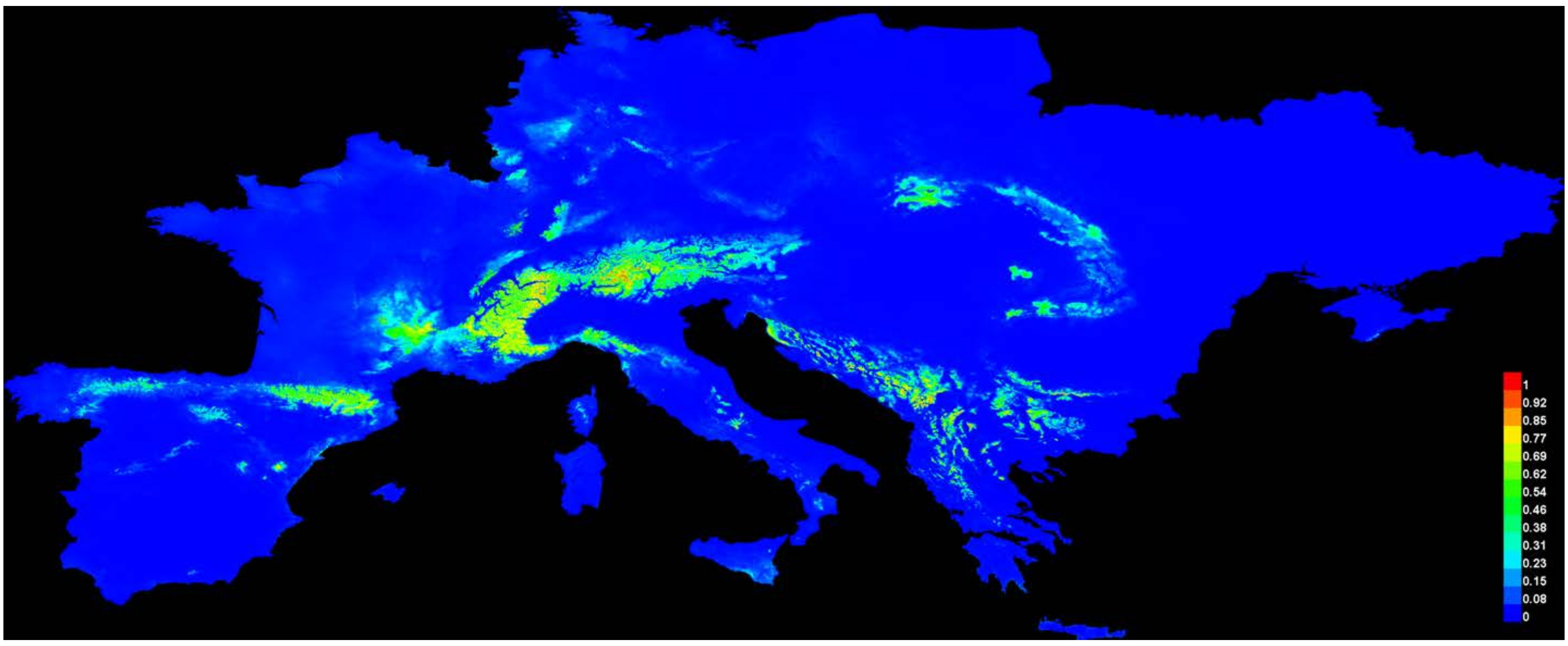

Fig. S15e

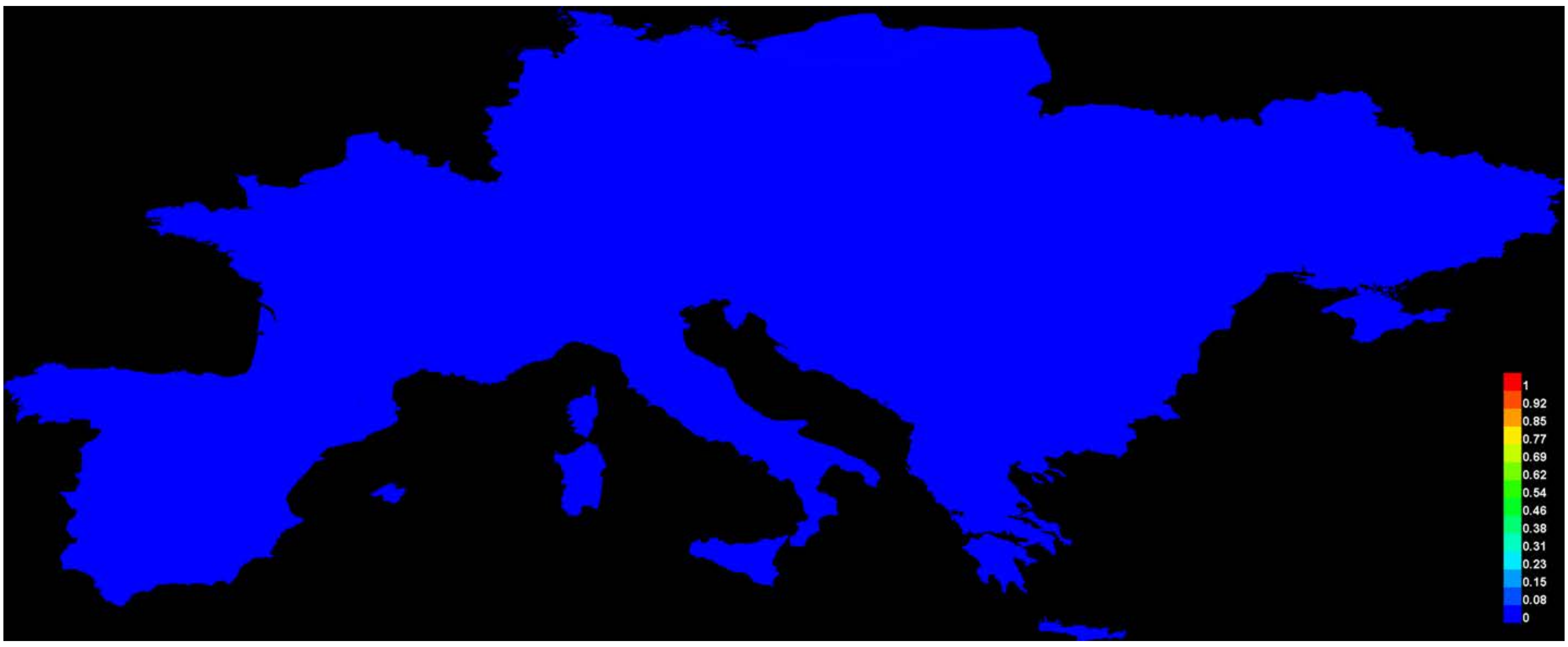

Fig. S15f

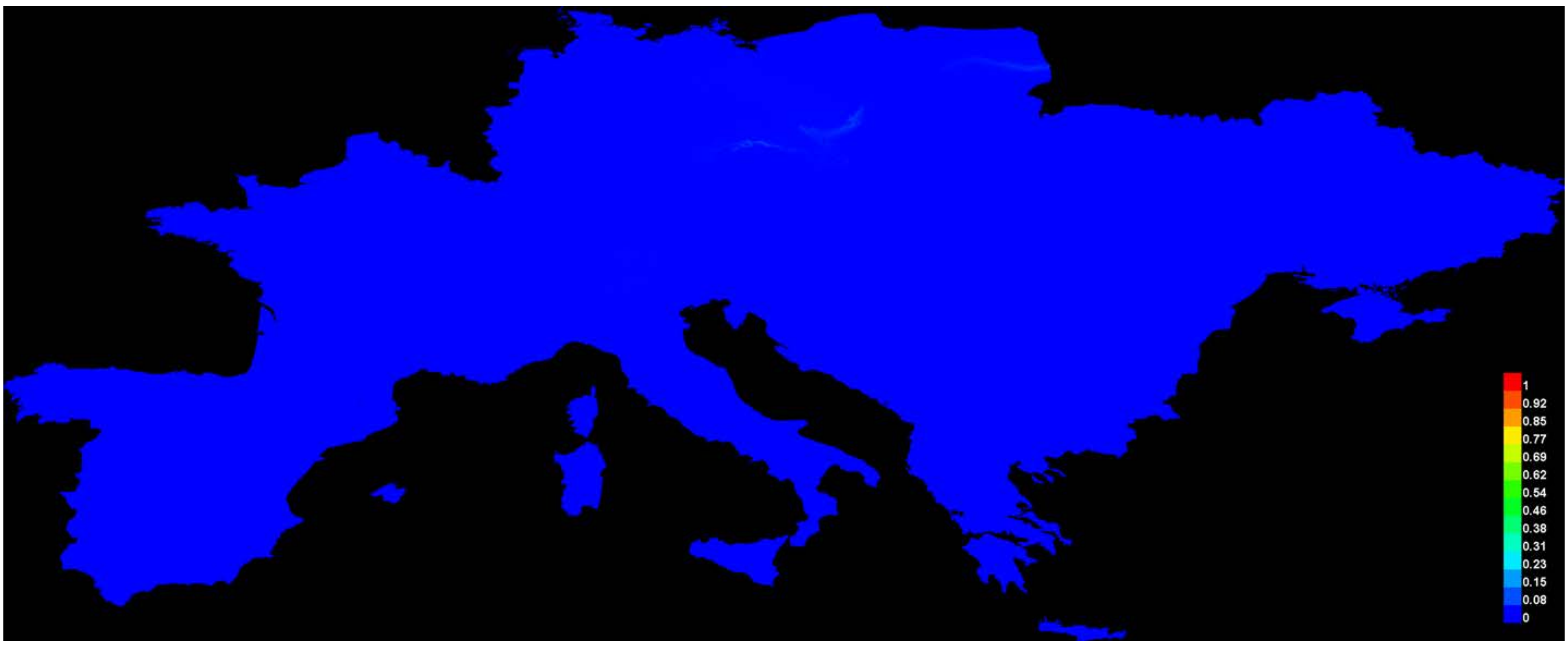

Fig. S15g

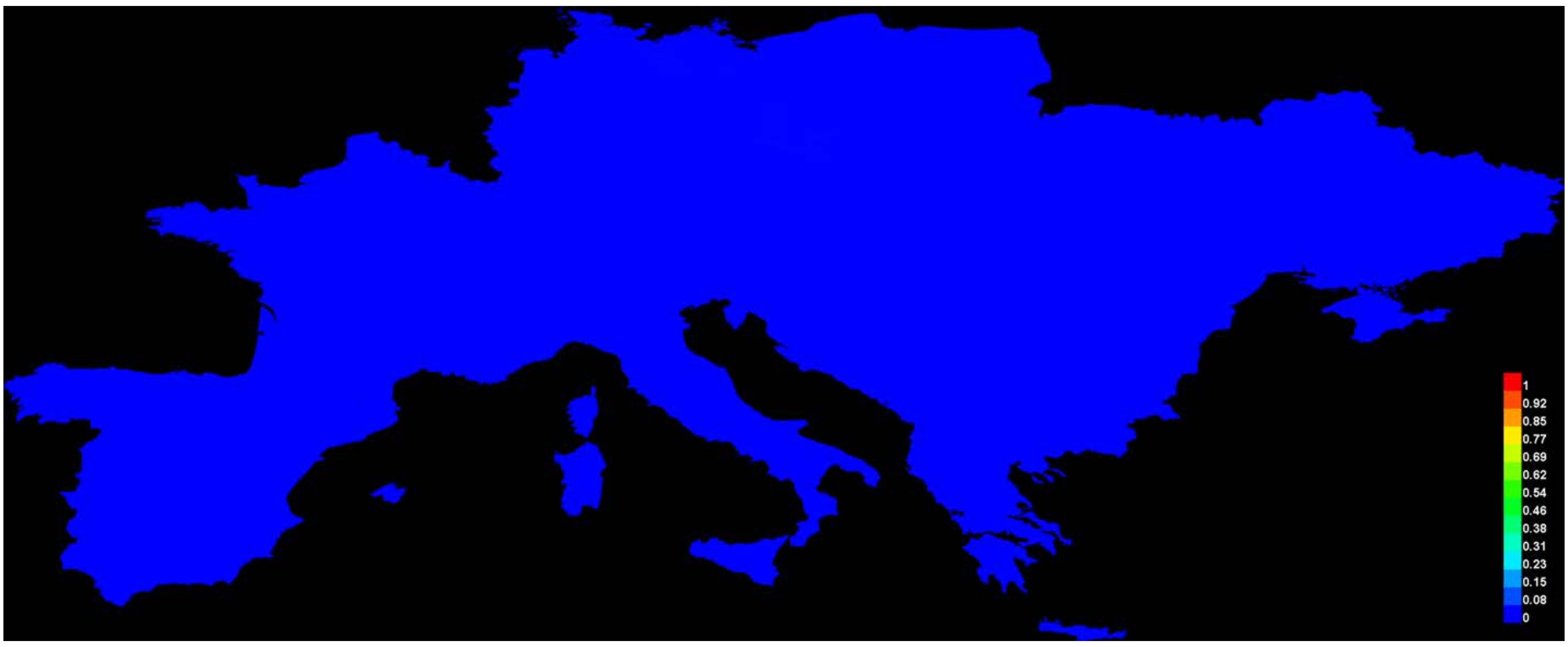

Supplement: Supplementary file 5 — Additional file 5: Fig. S13. Modeled species distributions for Cherleria capillacea. Models were from MaxEnt based on BioClim variables, with or without pH included. Models with pH are projected onto the LGM climate reconstructed with the CCSM4 (S13a), MIROC-ESM (S13b), and MPI-ESM-P (S13c) climate models. Models without pH are the current distribution (S13d) and its projection onto LGM climate reconstructed using the CCSM4 (S13e), MIROC-ESM (S13f) and MPI-ESM-P (S13g) climate models. Fig. S14. Modeled species distributions for Cherleria langii. Models were from MaxEnt based on BioClim variables, with or without pH included. Models with pH are projected onto the LGM climate reconstructed with the CCSM4 (S14a), MIROC-ESM (S14b), and MPI-ESM-P (S14c) climate models. Models without pH are the current distribution (S14d) and its projection onto LGM climate reconstructed using the CCSM4 (S14e), MIROC-ESM (S14f) and MPI-ESM-P (S14g) climate models. Fig. S15. Modeled species distributions for Cherleria laricifolia. Models were from MaxEnt based on BioClim variables, with or without pH included. Models with pH are projected onto the LGM climate reconstructed with the CCSM4 (S15a), MIROC-ESM (S15b) and MPI-ESM-P (S15c) climate models. Models without pH are the current distribution (S15d) and its projection onto LGM climate reconstructed using the CCSM4 (S15e), MIROC-ESM (S15f) and MPI-ESM-P (S15g) climate models. [file 12862_2020_1721_MOESM5_ESM.pdf]
